# Supplementary material for: Evolution of cortical geometry and its link to function, behaviour and ecology
Source: Nat Commun. 2023 Apr 20;14:2252. doi: 10.1038/s41467-023-37574-x (PMC10119184; doi:10.1038/s41467-023-37574-x)
Supplement: Supplementary file 1 — Supplementary Information [file 41467_2023_37574_MOESM1_ESM.pdf]

Supplementary Information for *Evolution of Cortical Geometry and its Link to Function, Behaviour and Ecology*

Ernst Schwartz[1], Karl-Heinz Nenning[1,2], Katja Heuer[3], Nathan Jefferey[4], Ornella C. Bertrand[5,7], Roberto Toro[3], Gregor Kasprian[1], Daniela Prayer[1], and Georg Langs[1,6]

[1]Department of Biomedical Imaging and Image-guided Therapy, Computational Imaging Research Lab, Medical University of Vienna, Vienna, Austria
[2]Center for Biomedical Imaging and Neuromodulation, Nathan Kline Institute, Orangeburg, NY, USA
[3]Institut Pasteur, Université Paris Cité, Unité de Neuroanatomie Appliquée et Théorique, F-75015 Paris, France
[4]Institute of Life Course and Medical Sciences, University of Liverpool, Liverpool, England
[5]Institut Català de Paleontologia Miquel Crusafont, Universitat Autònoma de Barcelona Edifici ICTA-ICP, c/ Columnes s/n, Campus de la UAB, 08193 Cerdanyola del Vallès. Barcelona, Spain
[6]Computer Science and Artificial Intelligence Lab, Massachusetts Institute of Technology, Cambridge, USA
[7]School of GeoSciences, University of Edinburgh, Grant Institute, Edinburgh, Scotland, EH9 3FE, United Kingdom

Supplementary Figures

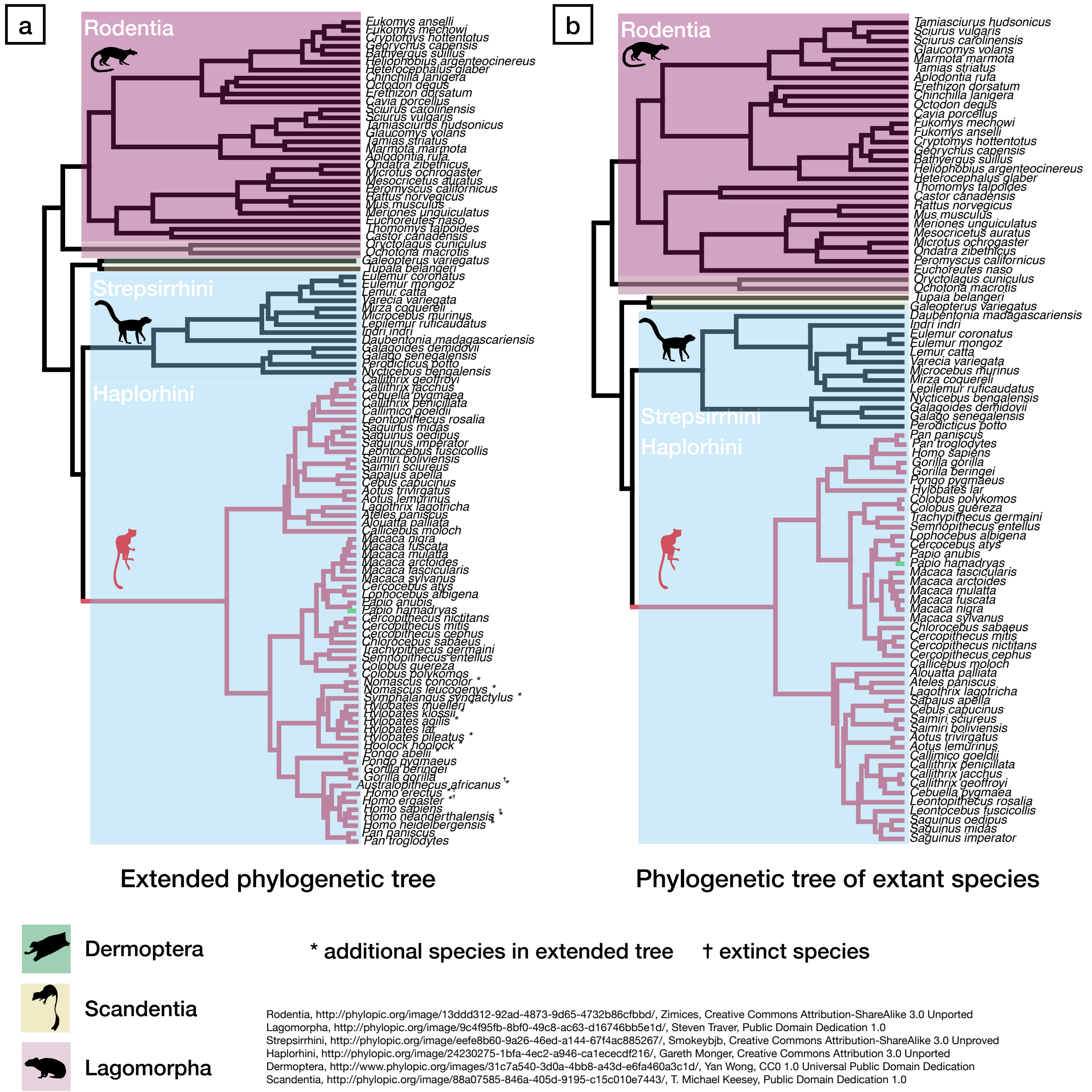

Supplementary Figure 1: Phylogenetic tree with shifts in evolutionary rate of global cortical shape changes. Ornstein-Uhlenbeck models with parameter shifts (coloured lines) were fitted to global shape parameters of cortical surface area and area of the convex hull of the cerebral hemisphere. (a) Measures from extinct hominin species were appended to the phylogenetic tree to perform fitting of evolutionary models. Parameter regimes are indicated as branch colours. (b) The phylogenetic tree of extant species used in this study.

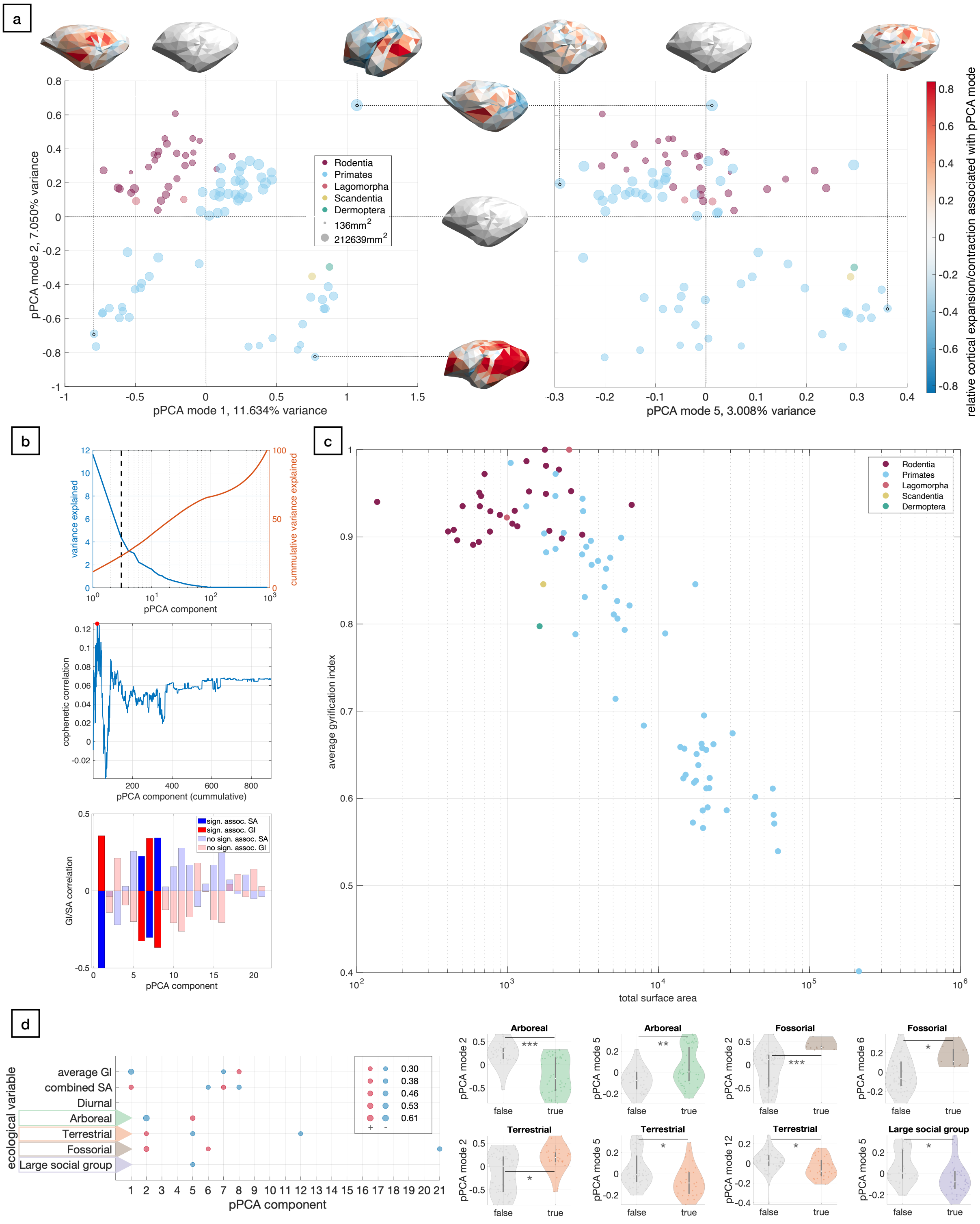

Supplementary Figure 2: (Caption on next page.)

Supplementary Figure 2: Scatter plot of total cortical surface area and gyrification index. Performing regularised phylogenetic pPCA on a down-sampled version of the studied dataset yielded 21 potentially informative shape dimensions. (a) Clade membership and overall brain size are discriminated in the first 2 pPCA dimensions, while pPCA dimension 5 is associated with preferred habitat and group size. (b) Fraction of the variance explained in each pPCA dimension is low. We retain the first 21 modes as the cophenetic correlation between shape and the underlying genetic phylogeny drops rapidly at higher dimensions. Individual dimensions are correlated with global shape measures, but of those related to ecological or behavioural variables only pPCA dimension 6 shows significant correlation with global parameters of cortical shape. (c) Overall, there exists a strong negative correlation between the surface area and the gyrification of the cortex ($\rho(90) = -0.82918$, 0.95 CI $[-0.884, -0.751]$, $p < 0.0001$). This relationship is conserved in primates ($r(58) = -0.92358$, 0.95 CI $[-0.959, -0.859]$, $p < 0.0001$), but not in rodents ($r(28) = 0.23403$, $[-0.176, 0.575]$, $p = 0.2307$, Supplementary Data 10a) (d) Violin plots of the values for pPCA components dimensions 2, 5, 6, 12 (21 not shown), together with median values as well as upper and lower quartile +/- 1.5 interquartile range. All are significantly ($n = 90$, * $q < 0.05$, ** $q < 0.01$, *** $q < 0.001$, FDR-corrected, Supplementary Data 10b) related to ecological variables encoding environmental niche and group size.

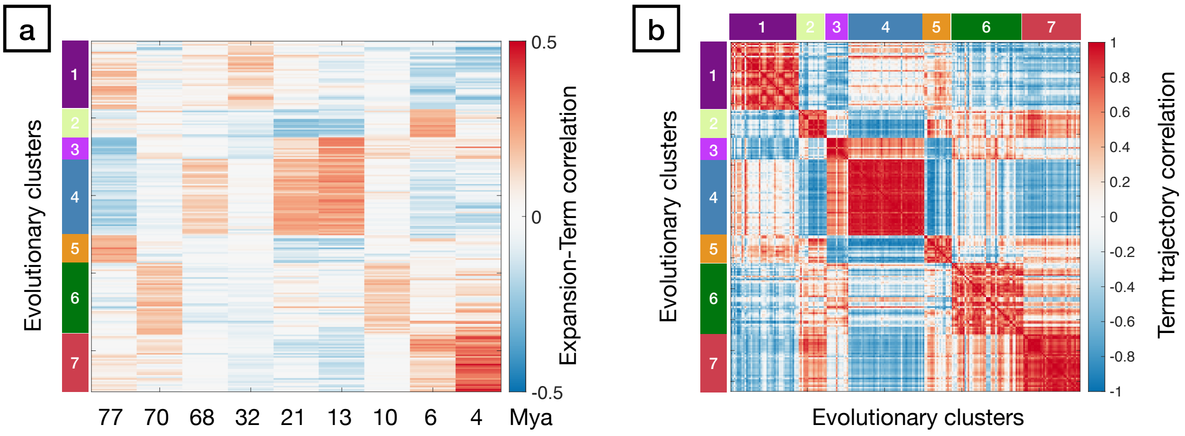

Supplementary Figure 3: (a) Time course of correlation between cortical expansion and term vocabulary. (b) pairwise correlation between time courses, sorted by cluster shows the validity of the proposed grouping (Figure 6).

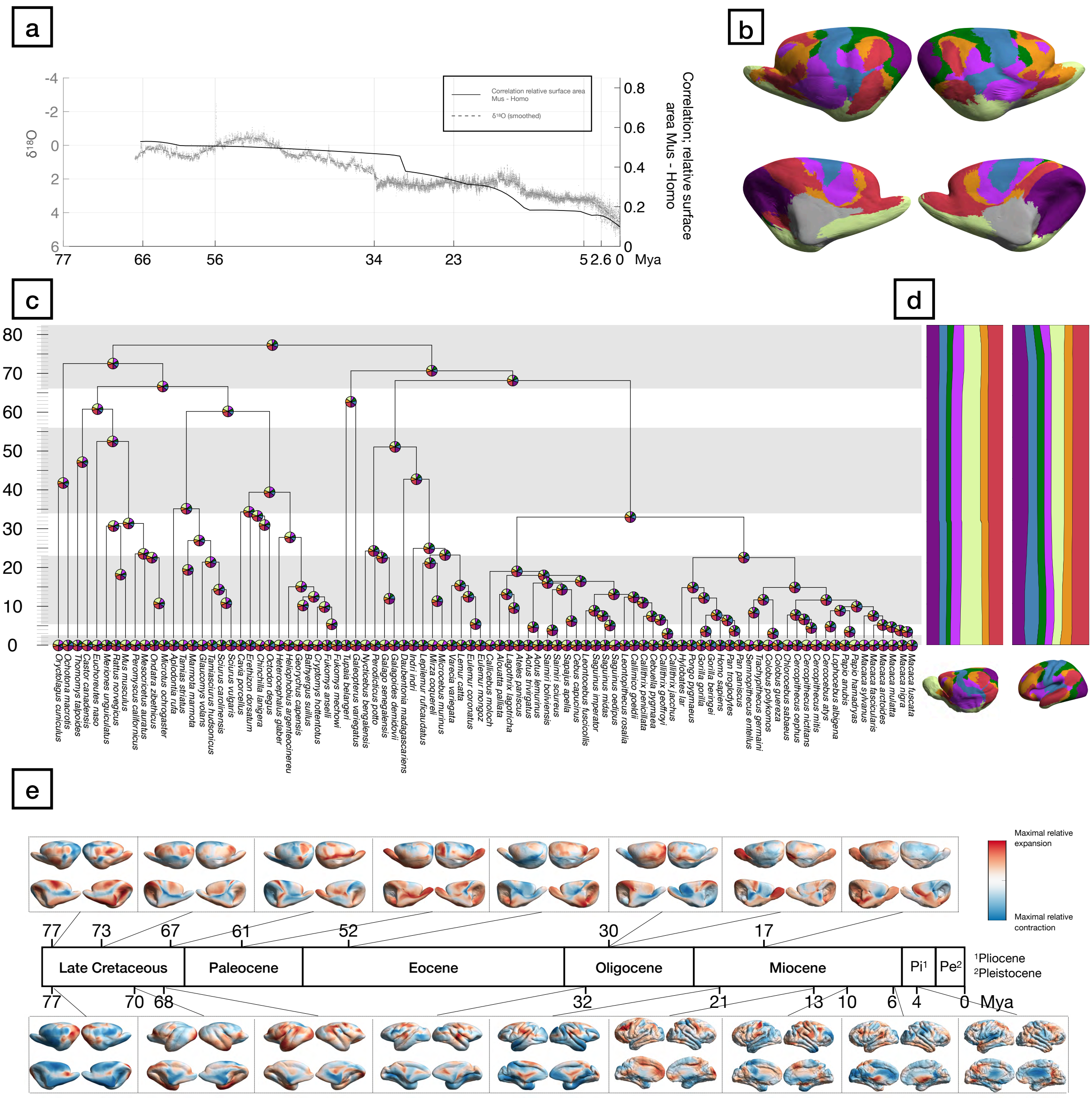

Supplementary Figure 4: (a) Estimated oxygen concentration in comparison with correlation between cortical surface area of mice and humans indicates influence of climatic changes to diverging evolutionary adaptation ($r = 0.915$, $p < 0.001$, 0.95 CI $[0.873, 0.944]$, Supplementary Data 4a). (b) Functional parcellation of the cerebral cortex obtained from human fMRI data plotted on the ancestral state reconstruction of the cortical surface model of the LCA of rodents and primates (c) Phylogenetic tree of the species of Euarchontoglires annotated with the relative extent of functional areas. (d) Comparison of the changes in relative area attributed to individual functionally defined regions of the cortex shows a sequence of potential evolutionary processes and highlights the distinctive reduction of the surface area attributed to limbic processes and the relative expansion of association areas in Homo. In Mus on the contrary, these regions dramatically expanded, mostly but not exclusively attributable to a dramatically expanded olfactory bulb. Corresponding statistical information is provided in Supplementary Data 4b. (e) Comparison between estimated relative local evolutionary cortical surface expansion in mice (top) and humans (bottom)

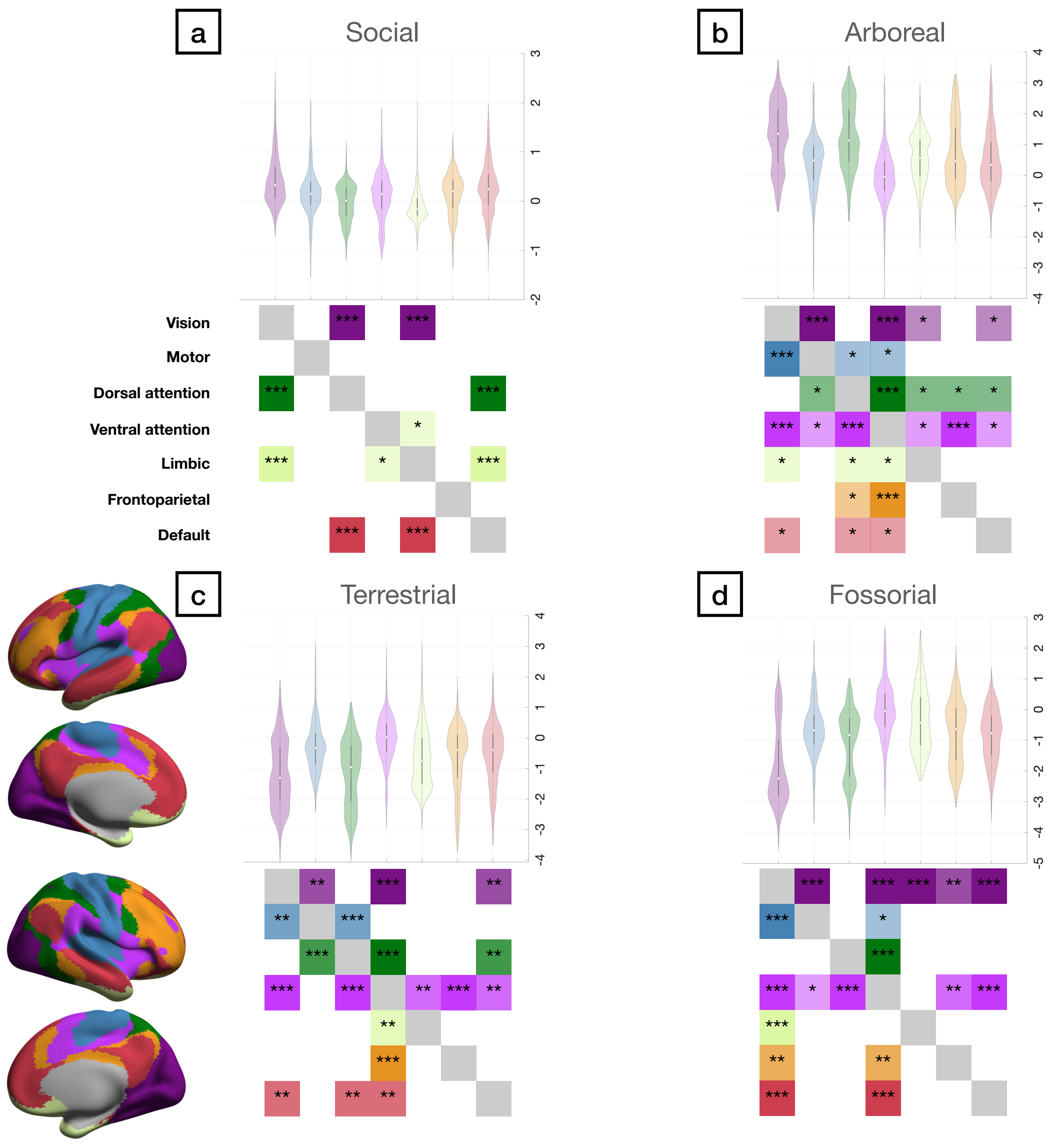

Supplementary Figure 5: Distribution of local expansion patterns in a functionally defined parcellation of the human cortex. Post-hoc analysis of the distribution of relative cortical expansion associated to (a) social group size, (b) arboreal, (c) terrestrial and (d) fossorial habitat demonstrates a mosaic-type nature of cortical shape changes. Boxplots represent the median with 1.5 times the interquartile range. Significant differences between expansion patterns are determined by post-hoc analysis using two-sided Kruskall-Wallis tests on a surrogate maps distribution and indicated by * $p < 0.05$, ** $p < 0.01$, *** $p < 0.001$ (Supplementary Data 5). The pattern of affected cortical areas in social group size is markedly different from the three variables related to habitat. The patterns of differential cortical expansion associated to arboreal, terrestrial and fossorial habitat shows strong topographic regularities, indicating a joint common factor.

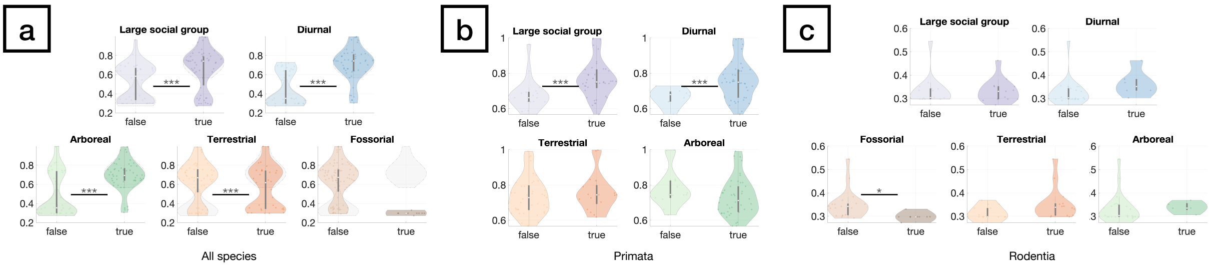

Supplementary Figure 6: Relationship of range parameters and ecological and behavioural variables. Spatial statistical models are fit to the relationship of modal specificity and geodesic distance on the cortex. The resulting range parameter yields a measure of relative distance below which there is a significant effect of spatial proximity on modal specificity. Results are reported for (a) all species ($n = 90$), (b) primates ($n = 58$), (c) rodents ($n = 28$). Significant effects as per two-sided t-test, FDR-corrected for multiple comparisons are denoted as *: < 0.05, ***: < 0.001. (Supplementary Data 6a). Boxplots represent the median and the lower/upper quartile +/- 1.5 interquartile range.

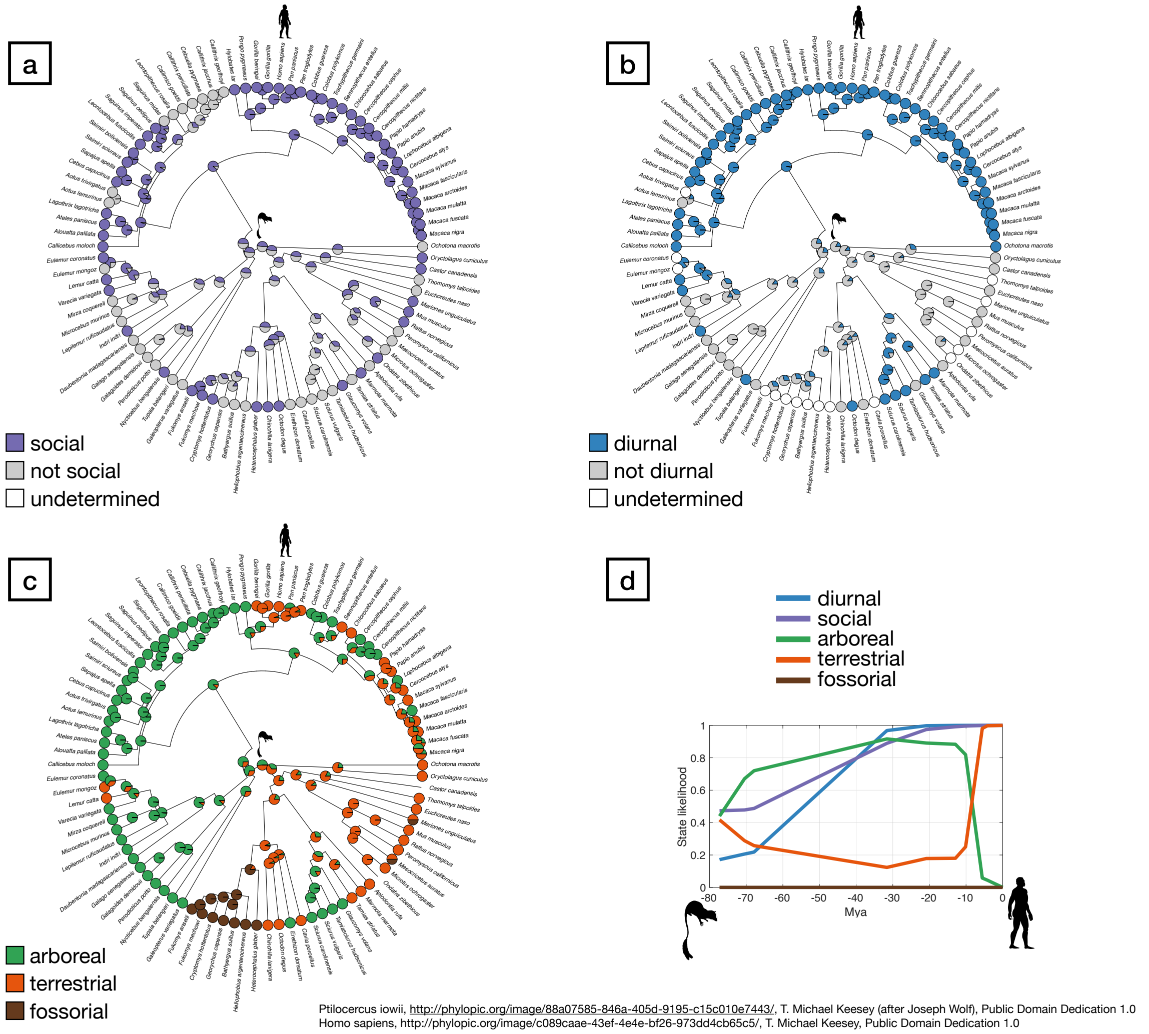

Supplementary Figure 7: Ancestral state reconstruction of binary encodings of ecological and behavioural variables. (a) large group size, (b) diurnality, (c) habitat. (d) progression of likelihoods of all parameters from LCA of rodents and primates to *Homo sapiens*.

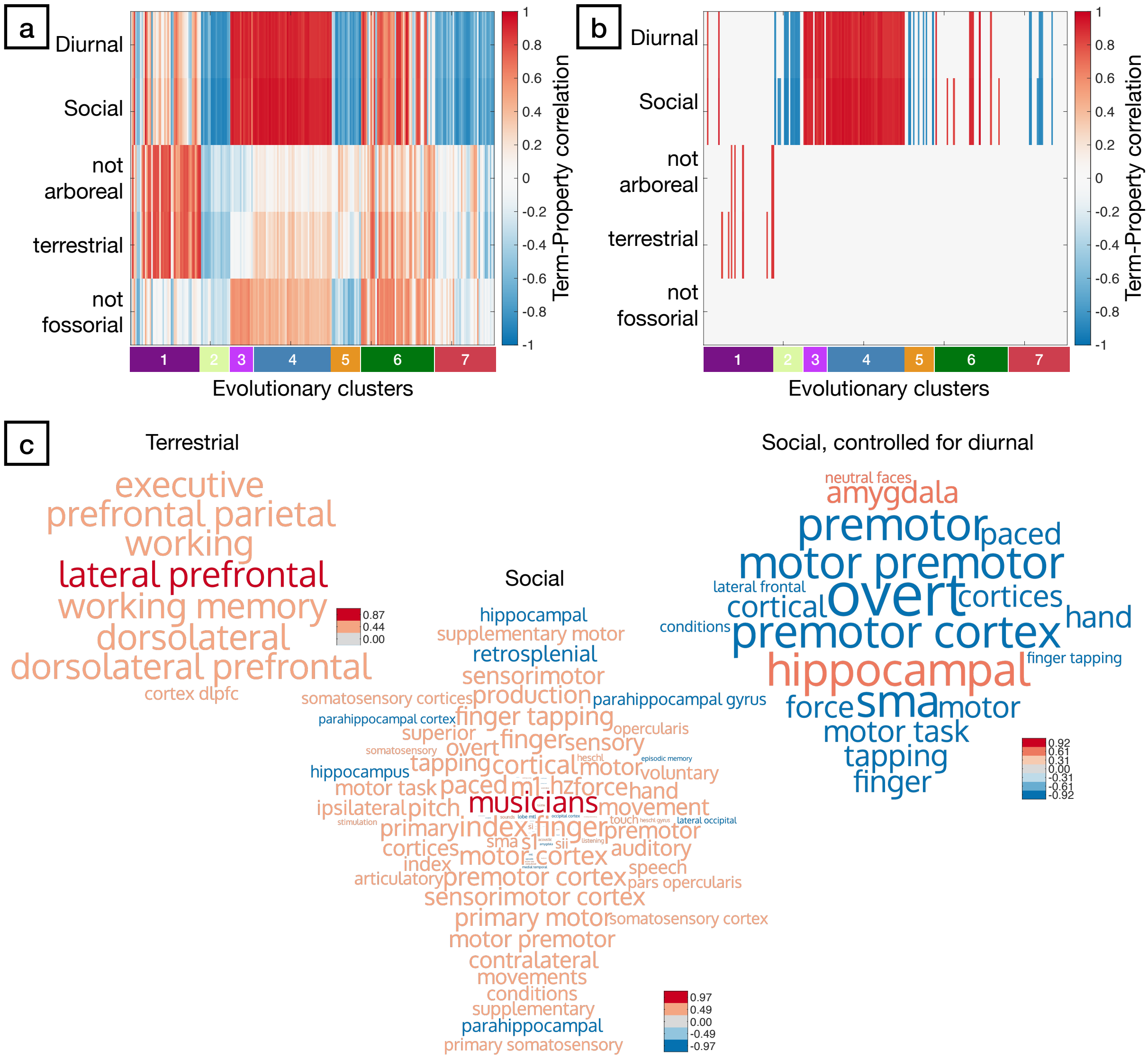

Supplementary Figure 8: Association between evolutionary progression of ecology and behaviour and cortical expansion of term-specific regions. (a) Showed the unthresholded, (b) the thresholded ($q <= 0.05$) correlations between the cumulative correlations between cortical expansion and statistical maps corresponding to neuro-scientific terms (Figure 6) and the progression of the likelihood of ancestral state reconstruction of ecological and behavioural variables (Supplementary Figure 1). (c) Terms showing significant correlation ($q <= 0.05$) or partial correlation ($q < 0.1$, 95% CI not covering 0) with the progression of the likelihood of terrestriality and sociallity in the deep ancestral human lineage (Supplementary Datas 9a, 9b).

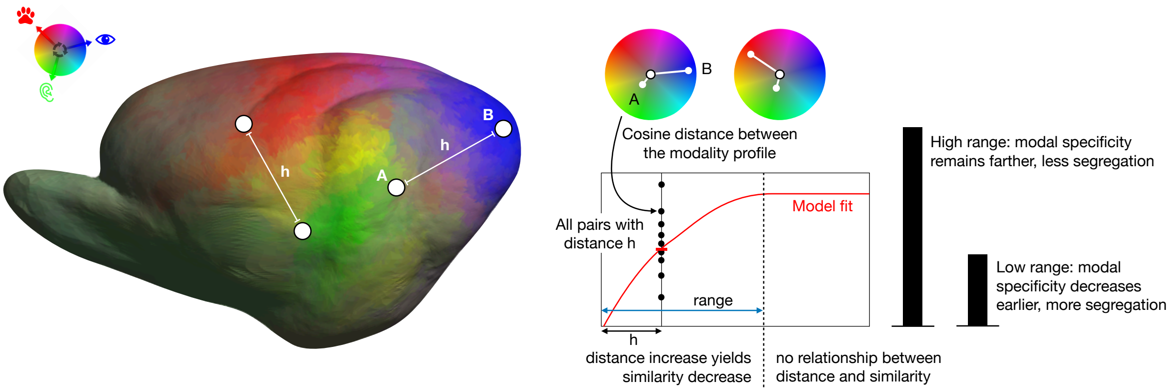

Supplementary Figure 9: Schematic of spatial statistical modelling of cortical modal specificity. The areas of the estimated cortical surface model for the common ancestor of Euarchontoglires are associated with a three-dimensional numerical representation of the estimate of their modal specificity for auditory, visual or somatosensory processing. A parametric model represents the relationship of the pairwise distances between these representations and the geodesic distances between the corresponding surface points. The range parameter of the fitted model encodes the distance at which spatial correlation of the profiles of modal specification vanishes, thereby yielding a measure of modal specificity for the geometric and topographic layout of the cortical surface.

Supplementary Methods

1 Phylogenetic modelling

The superorder Euarchontoglires groups together primates, rodents, lagomorphs, scandentians and dermopterans and as such represents roughly half of all extant mammals [1]. It is safe to assume that the evolutionary adaptive processes that enabled this wide variety of species to populate their individual socio-ecological niches a) had an impact on brain morphology and b) was neither random nor constant, precluding the utilisation of simple phylogenetic models for their analysis. In fact, recent work on both the shape of the overall brain [2] and its parts [3] demonstrated the existence of *rate shifts* in the neuroevolutionary processes in parts of the phylogenetic tree of primates.

1.1 Constructing the phylogenetic topology

The aim of this project is to analyse the phylogeny of the shape of the cerebral cortex in Euarchontoglires with minimal a-priori assumption on structural homologies between surfaces.

We base our analysis on a time-calibrated tip-dated Bayesian phylogeny obtained from a DNA supermatrix of 31 genes [4]. We draw 100 samples from the posterior distribution of the taxonomic subset containing the 90 species for which imaging data of the cerebrum was available. We then compute the strict consensus tree $\mathcal{T}$ of these 100 realisations on which we base further analysis. In order to account for the exceptional expansion of the cerebrum in hominins, we merge the resulting tree of extant species with an additional time-calibrated tree containing extinct 5 hominin species (Homo neanderthalensis, Homo heidelbergensis, Homo ergaster, Homo erectus, Australopithecus africanus) and 9 additional primates (Pongo abelii, Symphalangus syndactylus, Hylobates klossii, Hylobates muelleri, Hylobates agilis, Hylobates pileatus, Hoolock hoolock, Nomascus leucogenys, Nomascus concolor) using phylogenetic inference under matrix representation, resulting in a calibrated phylogeny $\mathcal{T}^+$ of 104 (99 extant and 5 extinct) species of Euarchontoglires. Note that for the 14 (9 extant and 5 extinct) species, only a simplified model of the brain endocasts was available.

In the tip-dated phylogeny, estimates of divergence times is obtained by statistical modelling of molecular processes that best explain the observed distribution of genetic data. However, a direct, uniform relationship between the time-frames of these molecular processes and cortical morphology is all but impossible. An ancestral state estimated directly using this molecular dating will therefore be incorrect.

Statistical estimation of the speed of morphological changes requires an a-priori definition of homologous landmarks. At the onset however, no correspondences exist between any of the 90 (104) cortical surface models. In fact, we purposefully avoid the definition of such landmarks to allow for uncertainties in cortical homologies, precluding the direct modelling of the process of the evolution of cortical shape.

In order to nonetheless be able to approximate scaling factors between the estimates of divergence time obtained from molecular data and morphological changes to the cerebral cortex, we make use of the fact that the cerebral cortex in mammals exhibits strong scaling rules relating overall cortical area and gyrification [5]. Phylogenetic modelling of these global shape parameters does not require any correspondences between the cortical surfaces. We assume that the evolutionary process that leads to changes in these global cortical shape parameters has a consistent relationship to the evolutionary process that affects local cortical morphology. This enables us to use the speed of evolutionary processes estimated from global shape features for estimating the interpolation factor between the cortical shapes of sister species in the phylogenetic tree before establishing correspondences between their surfaces.

1.2 Evolutionary model fitting

Evolutionary adaptation to changing environmental conditions and phenotyptical innovation in established niches result in constantly changing diversity of biological form. Mathematically, these dynamics can be modelled as stochastic processes, whose parameters can be estimated from the observation of extant phenotypes acting as boundary conditions. Definition of these models and estimation of their parameters is the goal of phylogenetic comparative methods (PCM) that enable the formulation and testing of evolutionary hypotheses.

1.2.1 Evolutionary models

Apart from trivial stationary models, the simplest evolutionary model is the Wiener process, which corresponds to the evolutionary hypothesis of completely random phenotypic change. Under this framework, each realisation of a phenotype is the result of both temporally and evolutionary independent sequential changes without bounds, thereby negating any form of evolutionary optimum for a phenotype. Due to this independence, continuous-valued phenotypes $\mathbf{x}$ at the tips of a phylogenetic tree that are assumed to have originated from a Wiener process can be modelled using a simple multivariate Gaussian distribution

$$x(t) \sim N(x(0), \sigma^2 \mathbf{C}) \tag{1}$$

where $x(0)$ corresponds to the ancestral state and σ to the evolutionary rate. $\mathbf{C}$ is the so-called phylogenetic variance-covariance matrix, which consists of the total edge lengths of the phylogenetic tree on the diagonals and the shared edge lengths for two taxa on the off-diagonals. Maximum likelihood estimates for the model parameters can then be obtained from observed $\mathbf{x}(1)$ at the tips of the phylogenetic tree with n tips as

$$\hat{x}(0) = (\mathbf{1}^\top \mathbf{C}^{-1} \mathbf{1})^{-1} (\mathbf{1}^{-1} \mathbf{C}^{-1} x(1)) \tag{2}$$

and

$$\hat{\sigma}^2_{ML} = \frac{(x(1) - \hat{x}(0)\mathbf{1})^\top \mathbf{C}^{-1} (x(1) - \hat{x}(0)\mathbf{1})}{n} \tag{3}$$

In the case where p correlated traits are being modelled, the variance of the multivariate Wiener process is given as

$$\mathbb{V}\text{ar}[\text{vec}(\mathbf{X})] = \mathbf{R} \otimes \mathbf{C} \tag{4}$$

where $\mathbf{X}$ is an n by p matrix of multivariate traits for each species in the phylogenetic tree, vec describes the stacking of its columns and $\mathbf{R}$ is the between-trait correlation matrix. In cases where the evolution of high-dimensional features is modelled, regularisation [2] can be used to estimate $\mathbf{R}$.

Due to adaptation to ecological niches, completely random evolutionary progression of phenotypes is unlikely. Rather, it has been shown that the distribution of a variety of notably neuroanatomical phenotypes are better explained by integrating a mechanism for mean reversion, eg. penalising a strong deviation from an adaptive optimum. This can be formulated using an Ornstein-Uhlenbeck (OU) process [6, 7] defined as

$$d\mathbf{X}(t) = \mathbf{A}(\boldsymbol{\beta} - \mathbf{X}(t))dt + \mathbf{R}d\mathbf{W}(t) \tag{5}$$

where $\mathbf{W}$ is again a Wiener process, $\boldsymbol{\beta}$ an *optimal* value for $\mathbf{X}$ and $\mathbf{A}$ encodes the selection strength of individual phenotypic variables and their interactions.

OU models have been used successfully in modelling the evolution of various aspects of neuroanatomy [8, 9]. However, notably in large phylogenies spanning long time-frames, optimal values for phenotypes might shift due to environmental changes.

1.2.2 Estimation of rate shifts

We are interested in modelling the evolutionary process that led to changes in the surface areas A of the cerebral cortices (S_{cort}) as well as their convex hulls (S_{hull}), which together allows us to deduce measures of gyrification ($G = \frac{A_{\text{hull}}}{A_{\text{cort}}}$). In order to estimate the location of shifts in the adaptive rate of the evolutionary process, we use the *scalar* OU (scOU) model proposed in [10]. We present only a brief overview of the model here, the reader is referred to the original publication for a detailed description of the model.

The scOU model is a simplified version of the multivariate OU of Equation 5, where $\mathbf{A} = \alpha\mathbf{I}$ is scalar. Under this model, the conditional distribution of a trait $\mathbf{X}^i$ at node i given its parent $\mathbf{X}^{\text{pa}(i)}$ is given as

$$\mathbf{X}^i \mid \mathbf{X}^{\text{pa}(i)} \sim N\bigg(e^{-\alpha l_i}\mathbf{X}^{\text{pa}(i)} + (1 - e^{-\alpha l_i})\boldsymbol{\beta}_i, \frac{1}{2\alpha}(1 - e^{\alpha l_i})\mathbf{R}\bigg) \tag{6}$$

where $\boldsymbol{\beta}_i = \boldsymbol{\beta}_{\text{pa}(i)} + \boldsymbol{\Delta}^i$ represents the optimal value of the process on the branch with length l_i going from pa(i) to i and $\boldsymbol{\Delta}$ is a matrix encoding the rate shifts.

This corresponds to the assumption that "all traits evolve at the same rate toward their respective optima" [10], while the correlation between the trait values is encoded in $\mathbf{R}$. In the present case, this implies a constant relationship between the surface areas of the cerebral cortex and that of its convex hull. Such strong scaling rules between cortical folding and surface area have in fact been shown to exist in mammals [5]. Note that we log-transform all surface area measurements before phylogenetic modelling. Another limitation of the scOU model is that it requires ultrametric trees, eg. the time-point for all tips of the underlying phylogenetic tree must be identical. Due to the inclusion of fossil taxa in the modelling, this is violated in our analysis. As the extinction times of the fossil taxa in our phylogeny are recent compared to the overall depth of the tree (Australopithecus africanus, approx 2.3Mya, relative to 78Mya), we simply extend the branches that terminate early to obtain an ultrametric tree $\mathcal{T}^+_{\text{um}}$.

Using the scalar simplifying assumption on the nature of $\mathbf{A}$, it is possible to formulate an Expectation–Maximisation (EM) algorithm for the iterative estimation of shift locations and model parameters [11]. During the Expectation step, the model parameters $\boldsymbol{\Delta}$ and $\mathbf{R}$ are fixed and used to estimate the ancestral states at the nodes of the phylogenetic tree. During the Maximisation Step, locations in the tree that exhibit large

differences for the trait values of the child and parent nodes are selected as potential shift locations. The number of shifts K as well as the value of α are determined by grid search. As shown in the supplementary material of [10], the scOU formulation leads to closed-form solution for the update of $\boldsymbol{\Delta}$ and $\mathbf{R}$. The EM algorithm terminates when no increase in likelihood can be obtained.

Fitting a scOU model to the two-dimensional vectors $[A_{\text{cort}}, A_{\text{hull}}]$ resulted in a model with 2 shifts, one located at the Haplorhini/Strepsirrhini divergence, the other species-specific to Papio hamadryas. We fix these shift locations and fit a full multivariate OU model [12] (without the scalar simplification used for estimating the shift locations) to the original combined tree $\mathcal{T}^+$.

2 Processing of cortical shapes

We base our analysis on cortical surface models obtained from volumetric imaging of the cerebra of ninety different species of Euarchontoglires. We obtain high-quality surface meshes with fixed topology for each cerebral hemisphere and extract salient features of their surface geometry.

2.1 Surface model construction

We obtain volumetric segmentations of the cerebral hemispheres from available atlases (7 species) as well as additional manual segmentations (83 species). As success of later surface matching is dependent on the quality of both the input meshes as well as their spherical parametrisation, we make use of numerous approaches of geometry optimisation to achieve regular surface sampling.

We iteratively optimise the triangulation of the cortical surface meshes as to avoid degenerate as well as unevenly sized surface triangles. We first obtain an initial surface mesh with spherical topology [13]. We then use instant field-aligned meshing (IFAM) [14] to optimise isotropy of the surface elements, and resolve any defects incurred by the resampling using the heuristics presented in [15]. We resample the resulting mesh again to a predetermined resolution (in the order of 10000 vertices per hemisphere for lissencephalic species, 40000 vertices for gyrencephalic species) using IFAM and resolve any remaining self-intersections by surface smoothing. We then ensure regularity of the surface sampling by constraining the range of areas of each element in the triangulation to lie between one half and twice the median surface area by either collapsing of subdividing triangles that fall outside this range.

Upon generation of a sufficiently regular surface sampling, we use robust fairing using Wilmore flow [16] to obtain a spherical parametrisation of the mesh. In some occasions, computation of the Wilmore flow fails due to the introduction of surface degeneracies. In this case, we use the more robust but less regular spherical parametrisation using spherical harmonics [17]. We then check if the spherical parametrisation of any surface triangle collapsed (at machine precision). The correpsonding vertices are then removed from the original surface and the spherical parametrisation is recomputed. We denote the final optimised spherical parametrisation as

$$f_{\text{sph}} : S_{\text{cort}} \to \Psi, \quad S_{\text{cort}} \in \mathbb{R}^3, \Psi \in \mathbb{S}^2 \tag{7}$$

In cases where segmentation of the gray matter was possible, both the white matter / gray matter boundary as well as the pial surface were computed as described. Pair-wise matching of surface points was then performed by simple orthogonal projection.

2.2 Cortical surface features

In order to constrain the matching of cortical surface models, we augment their geometric description (cf. section 3) by unequivocal anatomical landmarks and cortical surface features.

2.2.1 Anatomical features

One central goal of this work was to avoid the introduction of strong anatomical constraints to surface matching by limiting the use of anatomical landmarks for surface matching. However, Some structures show unequivocal homology between species. We therefore manually label the interhemispheric cleft, and segment the olfactory bulb (OB), the corpus callosum (CC) as well as for select species (XX) the thalamus, hypothalamus and septum. In XX post-mortem specimen, the OB has been removed during the preparation of the specimen and segmentation was therefore not possible. We interpolate the binary label values on the surface S_{cort} and compute the geodesic distance D_{geod} of each point $v_i \in S_{\text{cort}}$ to the closest point of each anatomical structure. From this, we compute a smooth indicator function I_{A} for the structure A as

$$I_{\text{A}}(v_i) = e^{-\frac{2D_{\text{geod}}(v_i)}{\max_i(D_{\text{geod}})}} \tag{8}$$

2.2.2 Geometrical features

In addition to the anatomical labels, we compute geometrical features F describing the cortical surface, especially the sulcation patterns. We approximate the sulcal depth (SD, F_{SD}) at each vertex as its (euclidean) distance to the convex hull of the cortical surface and compute its gradient $\boldsymbol{\nabla} F_{\text{SD}}$ on the mesh to add context on directionality of sulci.

In order to obtain additional information on location of sulci, we use a skeletonisation of the sulcal lines as described in [18]. Briefly, the method consists in first preforming a binary Graph-Cut segmentation of the mean curvature values of each vertex of the cortical surface to distinguish sulcal and gyral parts of the cortical topography. Similar to the definition of the anatomical labels above, a distance function D_{gyr} is computed as the geodesic distance of each vertex v_i of S_{cort}. The geodesic flux F_{flux} is then defined as

$$F_{\text{flux}}(v_i) = \frac{1}{r}\sum_{j=1}^{r} < \frac{v_j - v_i}{\|v_j - v_i\|}, \boldsymbol{\nabla} D_{\text{gyr}}(v_i) >, v_j \in \mathcal{N}(v_i), r = |\mathcal{N}(v_i)| \tag{9}$$

where $\mathcal{N}$ is the 1-ring neighbourhood of v_i in S_{cort}. The geodesic flux F_{flux} indicates locations of sulci as shocks in front-propagation on the cortex based on surface curvature.

At each vertex v_i, the anatomical and geometric information are then stored in vector form as $I_{\text{A}}(v_i)$ for every available structure A and

$$F(v_i) = [F_{\text{SD}}(v_i), \boldsymbol{\nabla} F_{\text{SD}}(v_i), F_{\text{flux}}(v_i)]$$

respectively.

3 Cortical surface matching

Pairwise surface matching is at the core of the results presented in this work. In the following, we describe the basic building blocks of our approach. To perform matching between the cortical surfaces of two sister species, we employ methods based on spectral shape representations, which are adapted to allow for non-rigid deformations. A-priori spherical embedding of the cortical surface meshes enables the computation of dense, continuous correspondences. Working in the space of smooth shells, these correspondences allow for the computation of an interim shape by interpolation. By approximating the global shape properties of the ancestral shape estimated from the a priori fitted evolutionary model, we can iterate pairwise matching and construction of a common topological space for all cortical surface in the available phylogeny of Euarchonotoglires.

3.1 Pairwise alignment

Morphological similarity is influenced by phylogeny. As such, evolutionary closer species exhibit greater shape similarities than more distantly related ones. Instead of aiming for direct group-wise alignment of cortical shapes, we therefore proceed locally in the phylogenetic tree, by performing iterative pair-wise alignments of sister species.

3.1.1 Spectral shape representation

We exploit spectral representations of the cortical surfaces of sister species S^0_{cort} and S^1_{cort} in $\mathcal{T}$ to establish pair-wise correspondences between their cortical surfaces. While correspondence between lissencephalic brain surfaces can be established in Euclidean space using standard methods, such direct matching of folded cortical surfaces can easily fail due to the complex geometry of the shapes. We therefore first computed a representation of each cortical shape in a high-dimensional spectral embedding space in which folding is not detrimental to matching performance.

The spectral shape representation of the surface mesh S_{cort} consisting of vertices v and triangles t is obtained by solving the following eigenproblem

$$A\phi = \lambda B\phi \tag{10}$$

for eigenvalues λ and eigenvectors V, with a mass matrix

$$B = \begin{cases} \frac{1}{12}(|t_1| + |t_2| & \text{if } j \text{ adjacent to } i \\ \frac{1}{6}(\sum_{k\in\mathcal{N}(i)} |t_k|) & \text{when } i = j \end{cases} \tag{11}$$

where $|t_1|$ and $|t_2|$ are the triangles that share an edge (i, j) and $|t_k|$ is the area of the triangle t_k. The stiffness matrix A is defined as

$$A = \begin{cases} w_{ij} = \frac{1}{2}(\cot\alpha_{ij}\cot\beta_{ij}) & \text{if } j \text{ adjacent to } i \\ -\sum_{j\in\mathcal{N}(i)} w_{ij} & \text{when } i = j \\ 0 & \text{otherwise} \end{cases} \tag{12}$$

with α_{ij} and β_{ij} are the angles in t_1 and t_2 respectively at the two vertices that are different from v_i and v_j.

The eigenvectors ϕ are invariant to isometric deformations of the corresponding mesh (v, t), and the locality or "frequency" [19] of the geometric information encoded in the the eigenvectors ϕ increases with the associated eigenvalues λ [20]. For matching two shapes S^0_{cort} and S^1_{cort} using their spectral representations (λ^0, ϕ^0) and (λ^1, ϕ^1), we thus sort the eigenvectors in ascending order and retain the eigenvectors corresponding to the 16 smallest eigenvalues.

3.1.2 Nonrigid matching

The spectral representations of two isometric shapes can be matched by standard methods for point-cloud alignment [21]. However, correctness of this matching relies on the existance of an isomorphism between the two shapes. As this assumption is violated for surfaces of different species, we employ a series of extensions to the standard spectral matching procedure to remove this limitation.

Appending extrinsic shape information The eigenvectors ϕ only encode intrinsic information about the shape - they for instance do not contain information about the direction of a fold on the surface, eg. cannot distinguish sulci and gyri. We therefore append the extrinsic shape features F and the anatomical indicator functions I (see 2.2) to the spectral representation of each cortical surface to be matched. The resulting combined shape encoding is then defined at each vertex on the surface and denoted $S_{\text{spec}}(v_i) = [\phi(v_i), F(v_i), I(v_i)] \in \mathbb{R}^d$, where the exact dimension $d >= 19$ of the feature vector depends on the availability of specific anatomical labels for a given pair of sister species.

Probabilistic spectral matching Exact correspondence between spectral representations is only guaranteed if an isometry exists between the two original shapes. This however is clearly not the case for the cerebral cortices of two different species that where subject to millions of years of separate evolution. In order to overcome this limitation, we use the probabilistic approach proposed in [22], where establishing of correspondences in the embedding space is formulated as a point cloud matching problem that is solved by Coherent Point Drift (CPD).

CPD reformulates the problem of aligning two n-dimensional point clouds (in the present case, the spectral representations $S^0_{\text{spec}} = \{v_m\}, m \in [1 \dots M]$ and $S^1_{\text{spec}} = \{u_n\}, n \in [1 \dots N]$) as fitting of a Gaussian Mixture Model (GMM) with centers $\{v_m\}$ that best explain the samples $\{u_n\}$. Fitting of the GMM can then be performed using an EM algorithm that iterates between computing the posterior probability distribution

$$\mathbf{P}_{mn} = \frac{\exp^{-\frac{1}{2}\left\| \frac{u_n - (v_m + \mathbf{G}(m,\cdot)\mathbf{W})}{\sigma^{\text{old}}} \right\|^2}}{\sum_{k=1}^{M} \exp^{-\frac{1}{2}\left\| \frac{u_n - (v_m + \mathbf{G}(k,\cdot)\mathbf{W})}{\sigma^{\text{old}}} \right\|^2} + c} \tag{13}$$

where $c = (2\pi\sigma^2)^{D/2} \frac{w}{1-w} \frac{M}{N}$ corresponds to a uniform distribution with weight w accounting for potential outliers in the data and $\mathbf{G}_{ij} = \exp^{-\frac{1}{2\beta^2}\|v_i - v_j\|^2}$ is an exponential kernel on the points v, and consequently solving for the matrix $\mathbf{W}$ in

$$(\mathbf{G} + \lambda\sigma^2 \text{diag}(\mathbf{P1})^{-1})\mathbf{W} = \text{diag}(\mathbf{P1})^{-1}\mathbf{PX} - \mathbf{Y} \tag{14}$$

which via the Gaussian kernel $\mathbf{G}$ describes a smooth deformation between v and u [22]. We denote the thus deformed shape encoding (spectral coordinates augmented by extrinsic features) $\hat{S}^0_{\text{spec}} = \{v_m + \mathbf{G}(m, \cdot)\mathbf{W}\}$.

The non-rigidly transformed shape encoding $\hat{S}^0_{\text{spec}}$ is aligned to S^1_{spec}. Points of S^1_{spec} that are aligned to $\{\hat{v}_m\} \in \hat{S}^0_{\text{spec}}$ can thus be determined as $\text{argmin}_n \|\hat{v}_m - u_n\|^2$, points of S^0_{spec} that are aligned to $u_n \in S^1_{\text{spec}}$ as $\text{argmin}_m \|u_n - \hat{v}_m\|^2$. In practice, point-correspondences can be encoded efficiently as permutation matrices $\text{argmin}_{\mathbf{T}^{12}} \|\mathbf{T}^{12}v - u\|^2$ and $\text{argmin}_{\mathbf{T}^{12}} \|\mathbf{T}^{21}u - v\|^2$ respectively, where $\mathbf{T}^{12}$ and $\mathbf{T}^{21}$ are (not necessarily square) permutation matrices.

While this alignment captures large-scale correspondences of regions of the shape quite well, there are some limitations that require further considerations. On the one hand, the way by which the localised extrinsic information provided by cortical sulcation features F affects the optimised spectral representation is difficult to assess - ideally, we would want to obtain a spectral representation for both shapes that adheres both to the intrinsic geometry of the shape as well as its sulcal geometry in a manner that is consistent between the two shapes. On the other hand, while alignment using CPD in the embedding space is able to enforce topological

consistency for the deformed spectral representation, this does not directly translate to the original Euclidean space, where topological inconsistencies can appear in the matched surface. Also, due to the discrete nature of the shape representation as triangular meshes, the correspondences do not commute.

Functional map refinement using iterative closest points The correspondences between extended shape representations S^1_{spec} and S^2_{spec} are based on both the spectral embedding ϕ and the extrinsic shape features F obtained from each shape. The extrinsic shape features are on purpose localised to the locations of the sulci in the case of geometric features and to the vicinity of anatomical structures for the label features. On the other hand, the spectral shape features ϕ encode the overall shape of the cortex, and vary smoothly over the whole surface. Ideally, we would want to combine these properties into one concise representation, eg. use the information provided by the extrinsic features to modify the spectral representation of the deformed shape $\hat{S}^1_{\text{spec}}$. To this aim, we employ the framework of functional maps [23, 24]. A functional map $\mathbf{M}$ is a linear map between the spectral representations ϕ^1 and ϕ^2 of the two shapes S^1_{spec} and S^2_{spec} thus the solution to

$$\operatorname*{argmin}_{\mathbf{M}} \|\mathbf{T}^{12}\phi^1\mathbf{M} - \phi^2\|^2 \tag{15}$$

after matching of the spectra via $\mathbf{T}^{12}$. The resulting modified shape spectrum $\hat{\phi}^1 = \phi^1\mathbf{M}$ then reflects the fact that the alignment $\mathbf{T}^{12}$ between S^1_{spec} and S^2_{spec} is obtained after inclusion of the extrinsic shape features, but represents a continuous, global map between the two shapes instead of point-wise correspondences.

Having computed an optimal $\mathbf{M}$ for $\mathbf{T}^{12}$, an updated correspondence matrix is estimated as

$$\operatorname*{argmin}_{\hat{\mathbf{T}}^{12}} \|\hat{\mathbf{T}}^{12}\phi^0\mathbf{M} - \phi^1\|^2 \tag{16}$$

Correspondence regularisation The steps taken so far to optimise the surface alignment operate purely in the spectral domain, and there is no guarantee that topological consistency in the spectral domain is preserved in the original Euclidean space. To account for this possible inconsistency, we employ a method of smoothing correspondences in Euclidean space proposed in [25]. Briefly, after obtaining a correspondence between ϕ^0_i and ϕ^1_j in the spectral domain, we obtain a smoothed spectral representation $\tilde{\phi}^1_j$ of ϕ^1_j as the weighted average of the spectral embedding $\phi^1_{\mathcal{N}_j}$ of the neighbours $u_k \in \mathcal{N}_j$ of the corresponding vertex u_j of S^1_{cort}, where weights are dependent on the distances $\|u_k - u_j\|^2$. We then determine the nearest neighbour of $\tilde{\phi}^1_j$ and ϕ^0_i as regularised correspondence. Applying the same procedure to both $\hat{\mathbf{T}}^0$ and $\mathbf{T}^{21}$ yields regularised correspondence matrices $\tilde{\mathbf{T}}^0$ and $\tilde{\mathbf{T}}^1$.

Joint Laplacian embedding The spectral representations $\phi^0\mathbf{M}$ and ϕ^1 are obtained from S^0_{cort} and S^1_{cort} individually. Therefore, despite the alignment using the functional map $\mathbf{M}$, they don't represent a common reference frame for both shapes. Such a common reference frame can be computed from the previously obtained point-wise correspondences $\tilde{\mathbf{T}}^0$ and $\tilde{\mathbf{T}}^1$ by means of a *joint Laplacian embedding.*

The joint Laplacian of the two surfaces S^0_{cort} and S^1_{cort} is obtained from the combined inter- and intra-shape neighbourhood matrix

$$\mathbf{J} = \begin{bmatrix} \mathbf{W^0} & \tilde{\mathbf{T}}^0 \\ \tilde{\mathbf{T}}^1 & \mathbf{W^1} \end{bmatrix} \tag{17}$$

where $\mathbf{A}^0_w$ and $\mathbf{A}^1_w$ are adjacency matrices weighted by the euclidean distance between vertices obtained from the mesh topologies of S^0_{cort} and S^1_{cort} respectively. By performing the spectral decomposition of the graph Laplacian

$$\mathbf{D}^{-1}(\mathbf{D} - \mathbf{J}) = \mathbf{U}\mathbf{\Lambda}\mathbf{U}^{-1}, \quad \mathbf{D}_{ij} = \sum_m \mathbf{J}_{im} \text{ for } i = j, \text{ 0 otherwise} \tag{18}$$

we obtain a joint spectral representation $\phi_J = [\phi^0_J; \phi^1_J]$ for both shapes S^0_{cort} and S^1_{cort}, where the first M rows correspond to the spectral embedding ϕ^0_J of S^0_{cort} and the remaining rows to the spectral embedding ϕ^1_J of S^1_{cort}. This set of embeddings is obtained by considering information from both the individual surface topologies and the previously computed correspondences between them. Thus, the nearest neighbours between ϕ^0_J and ϕ^1_J are regularised so as to penalise non-diffeomorphic mappings between the surfaces [26]. As before, we obtain updated point-wise correspondence maps from the joint spectral representations that we note as $\mathbf{T}^{12}_J$ and $\mathbf{T}^{21}_J$.

3.1.3 Harmonic consistent map

Reversibility is an essential property of a mapping between two shapes. Without it, important information might be lost when resampling one shape to the topology of the other for further processing. By their construction, $\mathbf{T}_J^{12}$ and $\mathbf{T}_J^{21}$ are not necessary inverses of each other. The last step of the pairwise matching procedure therefore consists in optimising $\mathbf{T}_J^{12}$ and $\mathbf{T}_J^{21}$ so that $\mathbf{T}_J^{12}\mathbf{T}_J^{21} \approx \mathbf{I}$. In practice, reversibility of the map between S^1_{cort} and S^2_{cort} is achieved by minimising the energy functional

$$E(\mathbf{T}^{12}, \mathbf{T}^{21}) = \alpha \sum_{\substack{i,j \in \{1,2\} \\ i \neq j}} \frac{1}{s_j} E_D(\mathbf{T}^{ij}) + (1-\alpha) E_R(\mathbf{T}^{12}, \mathbf{T}^{21}) \tag{19}$$

with

$$E_D(\mathbf{T}^{12}) = \sum_{(u,v) \in \mathcal{E}_1} w_{uv} d^2_{M_2}(\mathbf{T}^{12}u, \mathbf{T}^{12}v) \tag{20}$$

and

$$E_R(\mathbf{T}^{12}, \mathbf{T}^{21}) = \sum_{i,j \in \{1,2\}} \frac{1}{s_i^2} \sum_{p_i \in \mathcal{V}} d^2_{M_i}(\mathbf{T}^{ji}\mathbf{T}^{ij} p_i, p_i) A_i(p_i) \tag{21}$$

where s_i is the total area of a mesh M_i and w_{uv} the cotangent weight of the edge $(u.v)$.

Here, the term E_D is discrete Dirichlet energy that measures the smoothness of the mapping, while E_R is the reversibility energy, accounting for the deviation from identity in $\mathbf{T}^{12}\mathbf{T}^{21}$. Starting from the previously estimated correspondences $\mathbf{T}^{12}$ and $\mathbf{T}^{21}$, the energy (19) is solved by block coordinate descent, where we refer the reader to [27] for details on the optimisation procedure. At convergence, the final correspondences $\mathbf{T}^{12}_{\text{cons}}$ and $\mathbf{T}^{21}_{\text{cons}}$ exhibit both increased smoothness and reversibility.

3.1.4 Matching Primate and Rodent ancestor

We performed pair-wise matching of sister species throughut the Primate and Rodent clades using the spectral method outlined above. However, it proved inappropriate for aligning the estimated weighted average cortical shape of Rodentia, Dermoptera and Scandentia to the estimated weighted average of all Primates. The reason for this mismatch lies mostly in the exceptional expansion of the temporal cortex in primates compared to rodents. Due to this evolutionary event, the inherent preference of isomorphic alignment in the spectral method proved too strong, despite the remedying steps outline above.

However, both the weighted average of all cortical surfaces in rodents as well as in primates contains sufficient extrinsic information in terms of label maps (see Section 2.2.1 that provide anatomical coverage of the large parts of the neocortex to be used for feature-based alignment. At this point in the alignment procedure, we therefore use Spherical Demons [28] to establish correspondences between the two cortical shapes representative of rodents and primates.

3.2 Shape interpolation

After establishment of pairwise correspondences between the cortical surfaces of two sister species, iterative alignment of the cortices of all species in the phylogeny requires the definition of a shape that accurately approximates the ancestral state. Two possibilities exist for the calculation of an ancestral state: either, a deterministic generating process is known and can be formulated in an algorithmic fashion, or a large enough sample of generated shapes is available from which a statistical process can be estimated.

The phylogenetic comparative framework used in this paper falls into the second category, but requires comparative samples over which to perform statistical analysis. However, at the point where only individual pairs or subgroups of species have been aligned, computation of statistics is ill-posed, notably for high-dimensional objects such as shapes. We therefore approximate ancestral shapes by weighted averages of the daughter species, where the weights are derived from the a priori estimated global shape properties of surface area and gyrification. By working in the biomechanically inspired space of smooth shells [29, 30], we are able to generate approximations of the ancestral state that enables the recursive traversal of the phylogenic tree of Euarchontoglires. ´

3.2.1 Topology-consistent resampling

The result of surface matching is a pair of sparse matrices $\mathbf{T}^{12}$ and $\mathbf{T}^{21}$ that encode the correspondences between the cortical surface models S^1_{cort} and S^2_{cort} in the spectral domain such that $\mathbf{T}^{12}\hat{\phi}^1 \approx \phi^2$, where $\hat{\phi}^1$ and $\hat{\phi}^2$ are the spectral representations of the cortical shapes.

This correspondence in the spectral domain entails correspondences in Euclidean space, such that $\mathbf{T}^{12}S^1_{\text{cort}} \approx S^2_{\text{cort}}$. For each cortical surface, there exists a spherical parametrisation $f_{\text{sph}} : S_{\text{cort}} \to \Psi$, such that

$$\hat{S}^1_{\text{cort}} = f^{-1}_{\text{sph}}(\mathbf{T}^{12}\Psi^1) \tag{22}$$

corresponds to a resampled version of S^1_{cort} with the same topology as S^2_{cort} and matched vertices. The two shapes $\hat{S}^1_{\text{cort}}$ and S^2_{cort} are thus compatible in the sense that they have the same number of vertices whose locations correspond in the sense of both their geometry as encoded by their spectral representation as well as the extrinsic shape and anatomical features.

3.2.2 Estimation of interpolation factors between sister species

The goal of computing $\hat{S}^1_{\text{cort}}$ to be compatible with S^2_{cort} is to generate an intermediate shape that serves as an approximation to the shape of the cortical surface of their common ancestor $S^{\text{anc}}_{\text{cort}}$. However, a full ancestral state reconstruction cannot be performed from only two compatible shapes. Therefore, we instead approximate the ancestral state by a weighted average of the cortical models, where the weights are computed from the a priori computed ancestral state reconstruction $A^{\text{anc}} = [A^{\text{anc}}_{\text{cort}}, A^{\text{anc}}_{\text{hull}}]$ (see Section 1.2.2).

Formally, this corresponds to the weighting scheme

$$w^{12} = \frac{1}{2} \sum_{f \in \text{cort,hull}} \frac{A^{\text{anc}}_f - A^1_f}{A^2_f - A^1_f} \tag{23}$$

such that $A^{\text{anc}}_f \approx w^{12}A^1_f + (1 - w^{12})A^2_f$ which we use for construction of weighted averages of $\hat{S}^1_{\text{cort}}$ and S^2_{cort}.

3.2.3 Shell space representation

Direct computation of the weighted average of two cortical shapes in $\mathbb{R}^3$ can lead to biologically implausible results such as sharp edges or self-intersection. Instead, we perform computation of $S^{\text{avg}} \approx S^{\text{anc}}$ in the space of thin shells [29], thereby introducing elasticity constraints with respect to the shape of the surfaces of the daughter shapes when estimating an approximate ancestral state. While the induced linear elastic behaviour can only serve as a very rough approximation of the evolution of the biomechanical properties of the neocortex [31], interpolation in this geometry can be performed very efficiently [30]. Full biomechanical modelling at each node in the phylogenetic tree on the other hand would require knowledge of the evolutionary changes of the cellular constitution of the cerebral cortex and would furthermore be computationally prohibitive.

As described in [30], interpolation between two shapes in the space of discrete shells can be formulated as an energy minimisation problem. Instead of interpolating the vertex positions directly, elastic constraints are incorporated in the interpolation procedure by instead interpolating values for edge lengths, triangle areas and overall volume and consecutively reconstructing the vertex positions that correspond to these geometric parameters.

Specifically, the desired shape S^{avg} corresponds to the minimiser of the energy function

$$E = \lambda E_s + \mu E_b + \nu E_v \tag{24}$$

with

$$\begin{aligned} E_s &= \frac{1}{2}\sum_{e\in\mathcal{E}}(l_e - l^*_e)^2 \frac{1}{L^2_e} \\ E_b &= \frac{1}{2}\sum_{e\in\mathcal{E}}(\theta_e - \theta^*_e)^2 \frac{L^2_e}{A_e} \\ E_v &= \frac{1}{2}(\nu - \nu^*)\frac{1}{V^2} \end{aligned}$$

where

$$l^*_e = L^1_e + w^{12}(L^2_e - L^1_e) \tag{25}$$

are the desired edge lengths,

$$\theta_e^* = \Theta_e^1 + w^{12}(\Theta_e^2 - \Theta_e^1) \tag{26}$$

the desired dihedral angles, and

$$\nu^* = V^1 + w^{12}(V^2 - V^1) \tag{27}$$

the desired volume of S^{avg}, and λ, μ and ν weights for the stretching, bending and volume preservation terms. The surface that minimises the least squares error with respect to these desired geometric properties can be obtained by standard Gauss-Newton minimisation. Details of the solution to this optimisation problem are provided in [30].

It should be noted that vertex-wise weighting of these components is possible. As sulci exhibit considerable stability during evolution [32, 33], we decrease the contribution of bending at their location to penalise stretching. In practice, we also solve the inverse interpolation defined as

$$\tilde{l}_e^* = L_e^2 + (1 - w^{12})(L_e^1 - L_e^2) \tag{28}$$

$$\tilde{\theta}_e^* = \Theta_e^2 + (1 - w^{12})(\Theta_e^1 - \Theta_e^2) \tag{29}$$

$$\tilde{\nu}^* = V^2 + (1 - w^{12})(V^1 - V^2) \tag{30}$$

$$\tag{31}$$

and average the two resulting shapes in $\mathbb{R}^3$.

3.3 Label propagation

Estimation of the weighted average shape S^{avg} is based on both intrinsic and extrinsic shape properties of the two sister species S^1_{cort} and S^2_{cort} as well as label information encoded via geodesic distance maps on the cortical surfaces (see 2.2.1). While shape properties can be computed directly from the estimated S^{avg}, anatomical information has to be mapped into the new shape from S^1_{cort} and S^2_{cort}. As we map the topology of I^2_A of S^2_{cort} is kept fixed, while the anatomical information I^1_A is resampled using the previously estimated correspondences as in Equation 22 to yield $\hat{I}^1_A = f^{-1}_{\text{sph}}(\mathbf{T}^{12} I^1_A)$. Inadvertently, this leads to inconsistencies, as anatomical and geometric information are generally not in perfect agreement, eg generally $\hat{I}^1_A(v_i) \neq I^2_A(u_i)$.

A simple solution to the problem of reconciling mismatches between the anatomical features mapped from sister species in their average space would be to use some form of average such as the arithmetic or geometric means. However, this leads to blurring of the anatomical information and therefore a loss of precision in the definition of these areas. To counter these effects, we instead compute the average of $\hat{I}^1_A(v_i)$ $I^2_A(u_i)$ using Wasserstein distances on the surface S^{avg}.

Briefly, Wasserstein distance measures the amount of *work* that is required to *transport* the mass contained in one probability distribution onto another. Minimisers of Wasserstein distances are therefore also called optimal transport solutions. Formally, the 2-Wasserstein distance between the two probabilities $\hat{I}^1_A$ and I^2_A we seek to minimise is defined as

$$\mathcal{W}_2(\hat{I}^1_A, I^2_A) = \left[\inf_{\pi \in \Pi(\hat{I}^1_A, I^2_A)} \int\int_{M \times M} d(x,y)^2 \mathrm{d}\pi(x,y) \right]^{\frac{1}{2}} \tag{32}$$

where M is a compact, connected Riemannian manifold whose discretisation if S^{avg}, $d(x,y)$ is the shortest distance between locations x and y in M and

$$\Pi(\hat{I}^1_A, I^2_A) = \left\{ \pi \in \text{Prob}(M \times M), \quad \pi(\cdot, M) = \hat{I}^1_A, \pi(M, \cdot) = I^2_A \right\} \tag{33}$$

is the set of possible transports or joint probability measures on the product space of M.

This formulation can be used to define a *Wasserstein barycenters* as solution to

$$\min_{I^{\text{anc}}} \frac{1}{2} \Big(\mathcal{W}_2^2(I^{\text{anc}}, \hat{I}^1_A) + \mathcal{W}_2^2(I^{\text{anc}}, I^2_A) \Big) \tag{34}$$

An efficient approach to solving for I^{anc} are convolutional Wasserstein distances [34], which computes Wasserstein distances using iterative kernel convolutions and can be discretised on triangulated surfaces. We refer the reader to the original publication for a detailed description of the method.

4 Estimating the modal topography of the cerebral cortex

The cerebral cortex of mammals exhibits a strongly preserved layout of regions that perform processing of specific modal input. We used spatial statistical modelling to quantify the evolutionary and ethological effects onto this topograhy.

4.1 Quantifying information on sensory modality

While brain regions never fulfil only individual sensory processes, the distinction between primary and associative cortical areas is well-established. We quantified this modal specificity using the mathematical framework of spatial statistics.

4.1.1 Estimating modal specificity of cortical areas

We obtain estimates for the modal specificity of brain regions from a previously published parcellation of the human cortex [35] consisting of 180 areas per cortical hemisphere. The colorisation is based on modelling the contribution of modality-specific regions to each area. For this, the mean Blood Oxygen Level Dependent (BOLD) signal over a population of 1200 resting state acquisitions was computed and averaged for each region in the parcellation. Consequently, the signals of core somatosensory, auditory and visual regions were extracted and used (together with signals from task positive and task negative regions) for multiple regression of the signals in all 180 parcels, resulting in a 180 by 5 partial beta matrix. From this, we only retain the components corresponding to information on the modal specificity. After appropriate scaling, $\mathbf{m} = [m_{\text{motor}} m_{\text{audio}} m_{\text{visual}}]$, $\|\mathbf{m}\|^2 < 1$ reflects the modal specificity of each region in the parcellation.

4.1.2 Propagation of modal information to the common reference space

The modal specificity $\mathbf{m}$ is constant in every area of the parcellation of the human cortex [35]. The number of cortical areas however increased with evolution [36] by various topological changes [37]. Thus, direct utilisation of the discretised map $\mathbf{m}$ is bound to introduce bias in the analysis of the evolution of the functional topography of the cerebral cortex. On the other hand, numerous parcellations of individual species of Euarchontoglires have been published [38–42].

While not a perfect fit, we work under the assumption that the cytoarchitectural boundaries reflect a certain amount of functional segregation of the cerebral cortex. We propose to leverage the structural information contained in the parcellations of individual species in order to generate a smoothed estimate of the *potential* functional specificity of regions of the cerebral cortex. For this, we first retrieve the value of $\mathbf{m}$ at every location x of the human cortical surface model. Having mapped the cortical surfaces to a common reference topology. we aggregate the values $\mathbf{m}$ in each area of each species-specific parcellation. As cytoarchitectonic parcellations of the whole cortex are available for five species (Rat, Mouse, Marmoset, Macaque and Humans), we obtain $\mathbf{m}^s(x), s \in [1 \ldots 5]$. We consequently perform ancestral state reconstruction of the values of $\mathbf{m}^s(x)$ at the root node of the phylogeny $\mathcal{T}_{\mathbf{cyto}} \subset \mathcal{T}$, the subtree containing only the species for which cytoarchitectonic atlases were available. The result $\mathbf{m}_{\mathbf{anc}}$ of this procedure represents a smoothing of the original estimates obtained from data recorded in humans, constrained by both the cytoarchitecture of the five species as well as their phylogenetic relationship.

4.2 Spatial statistics

Specificity to processing of a certain input modality is an evolutionary conserved structuring principle of the topography of the cerebral cortex [43]. We quantified both the distribution of this topography in extant species of Euarchontoglires as well as its evolution using spatial statistics. By analysing the relationship between the distance between locations on the cortical surface and their difference in modal specificity, we are able to demonstrate a relationship of this modal topography with the species ethology as well as its evolution.

4.2.1 Empirical variogram of modal specificity

We compute the empirical variogram of modal specificity by relating geodesic distances $d_{\mathbf{geod}}(x, y)$ between two vertices x and y of a surface $\S_{\text{cort}}$ with the cosine distance between their modal specificity values as

$$d_{\mathbf{moda}}(x, y) = 1 - \frac{\mathbf{m}(x) \cdot \mathbf{m}(y)}{\|\mathbf{m}(x)\| \|\mathbf{m}(y)\|} \tag{35}$$

where we downsample the surface $\S_{\text{cort}}$ to 2562 vertices to make computation of the full distance matrices more efficient.

The empirical semivariogram of these measurements is defined as

$$\hat{\gamma}(h \pm \delta) = \frac{1}{2|N(h \pm \delta)|} \sum_{(x,y) \in N(h \pm \delta)} d_{\mathbf{moda}}(x, y) \tag{36}$$

which corresponds to a quantisation of the resulting geodesic distances into bins of centers h and equal width 2δ and the computation of the average modality difference for the corresponding vertices. We used 20 bins for discretisation, where we aggregated the values for both cerebral hemispheres. As we are interested in effects of relative cortical expansion, we scaled the resulting distribution of pairwise geodesic distances in all cases to 1. And as the measurements of modal specificity are identical for all cases, we scaled those to a maximum of 1 in each case as well - small deviations from 1 can otherwise be introduced due to distortions in the modal specificity measures from resampling. The resulting function $\hat{\gamma}(h)$ summarises the structure of the spatial autocorrelation of modal specificity for an individual cortical surface.

We computed these empirical semi-variograms $\hat{\gamma}$ for each cortical surface model in the phylogeny, as well as on the ancestral shape reconstructions at the internal nodes.

4.2.2 Parametric models of spatial autocorrelation

The spatial autocorrelation structure measured by the semi-variogram can be summarised by three factors, known in the field as *nugget*, *sill* and *range*.

The *nugget* of a semivariogram is the value at $\hat{\gamma}(0)$, eg. the minimal observed difference between the measurements. In the present case of modal topography of the cerebral cortex, the nugget is always 0 due to strong local autocorrelation in the underlying BOLD signal and the smoothing effect induced in the computation of $\mathbf{m}$ (cf. section 4.1.2). As we normalised both the maximal geodesic distance as well as the maximal difference in modal specificity to 1, the sill also contains no information. Instead, we are interested in comparing the value of the range between species, as it indicates the distance after which the modal specificity of two regions show no spatial dependence.

Different parametric models can be used to model the empirical semi-variogram. We fit 100 randomly initialised bilinear, spherical and exponential models to the semi-variogram of each species using a Nelder-Mead simplex method [44]. We then retain the model that yields the lowest average squared residual error over all runs and thereby obtain estimates for the range parameter of the semi-variogram.

References

1. Fostowicz-Frelik, L., Ge, D. & Ruf, I. Recent Advances in the Evolution of Euarchontoglires. *Frontiers in Genetics,* 1994.
2. Clavel, J., Aristide, L. & Morlon, H. A Penalized Likelihood Framework for High-Dimensional Phylogenetic Comparative Methods and an Application to New-World Monkeys Brain Evolution. en. *Syst. Biol.* **68,** 93–116 (Jan. 2019).
3. Schilder, B. M., Petry, H. M. & Hof, P. R. Evolutionary shifts dramatically reorganized the human hippocampal complex. en. *J. Comp. Neurol.* **528,** 3143–3170 (Dec. 2020).
4. Upham, N. S., Esselstyn, J. A. & Jetz, W. Inferring the mammal tree: Species-level sets of phylogenies for questions in ecology, evolution, and conservation. en. *PLoS Biol.* **17,** e3000494 (Dec. 2019).
5. Mota, B. & Herculano-Houzel, S. Cortical folding scales universally with surface area and thickness, not number of neurons. en. *Science* **349,** 74–77 (July 2015).
6. Butler, M. A. & King, A. A. Phylogenetic comparative analysis: a modeling approach for adaptive evolution. *The American Naturalist* **164,** 683–695 (2004).
7. Hansen, T. F. Stabilizing selection and the comparative analysis of adaptation. *Evolution* **51,** 1341–1351 (1997).
8. Ksepka, D. T. *et al.* Tempo and pattern of avian brain size evolution. *Current Biology* **30,** 2026–2036 (2020).
9. Smaers, J. B., Gómez-Robles, A., Parks, A. N. & Sherwood, C. C. Exceptional evolutionary expansion of prefrontal cortex in great apes and humans. *Current Biology* **27,** 714–720 (2017).
10. Bastide, P., Ané, C., Robin, S. & Mariadassou, M. Inference of Adaptive Shifts for Multivariate Correlated Traits. en. *Syst. Biol.* **67,** 662–680 (July 2018).
11. Bastide, P., Mariadassou, M. & Robin, S. *Detection of adaptive shifts on phylogenies by using shifted stochastic processes on a tree* 2017.

12. Clavel, J., Escarguel, G. & Merceron, G. Mv morph : An r package for fitting multivariate evolutionary models to morphometric data. en. *Methods Ecol. Evol.* **6,** 1311–1319 (Nov. 2015).
13. Yotter, R. A., Dahnke, R. & Gaser, C. *Topological Correction of Brain Surface Meshes Using Spherical Harmonics* in *Medical Image Computing and Computer-Assisted Intervention – MICCAI 2009* (Springer Berlin Heidelberg, 2009), 125–132.
14. Jakob, W., Tarini, M., Panozzo, D. & Sorkine-Hornung, O. Instant field-aligned meshes. *ACM Trans. Graph.* (2015).
15. Attene, M. A lightweight approach to repairing digitized polygon meshes. *The visual computer* **26,** 1393–1406 (2010).
16. Crane, K., Pinkall, U. & Schröder, P. Robust fairing via conformal curvature flow. *ACM Trans. Graph.* **32,** 1–10 (July 2013).
17. Choi, P. T., Lam, K. C. & Lui, L. M. FLASH: Fast landmark aligned spherical harmonic parameterization for genus-0 closed brain surfaces. *SIAM Journal on Imaging Sciences* **8,** 67–94 (2015).
18. Shi, Y., Thompson, P. M., Dinov, I. & Toga, A. W. *Hamilton–Jacobi Skeleton on Cortical Surfaces* 2008.
19. Reuter, M., Wolter, F.-E., Shenton, M. & Niethammer, M. *Laplace–Beltrami eigenvalues and topological features of eigenfunctions for statistical shape analysis* 2009.
20. Reuter, M., Wolter, F.-E. & Peinecke, N. Laplace–Beltrami spectra as 'Shape-DNA'of surfaces and solids. *Computer-Aided Design* **38,** 342–366 (2006).
21. Besl, P. J. & McKay, N. D. *Method for registration of 3-D shapes* in *Sensor fusion IV: control paradigms and data structures* **1611** (1992), 586–606.
22. Lombaert, H., Grady, L., Polimeni, J. R. & Cheriet, F. FOCUSR: feature oriented correspondence using spectral regularization–a method for precise surface matching. *IEEE transactions on pattern analysis and machine intelligence* **35,** 2143–2160 (2012).
23. Lombaert, H., Arcaro, M. & Ayache, N. *Brain Transfer: Spectral Analysis of Cortical Surfaces and Functional Maps* 2015.
24. Ovsjanikov, M., Ben-Chen, M., Solomon, J., Butscher, A. & Guibas, L. Functional maps: a flexible representation of maps between shapes. *ACM Trans. Graph.* **31,** 1–11 (July 2012).
25. Wright, R. *et al. Construction of a fetal spatio-temporal cortical surface atlas from in utero MRI: Application of spectral surface matching* 2015.
26. Lombaert, H., Sporring, J. & Siddiqi, K. Diffeomorphic spectral matching of cortical surfaces. en. *Inf. Process. Med. Imaging* **23,** 376–389 (2013).
27. Ezuz, D., Solomon, J. & Ben-Chen, M. *Reversible Harmonic Maps between Discrete Surfaces* 2019.
28. Yeo, B. T. *et al.* Spherical demons: fast diffeomorphic landmark-free surface registration. *IEEE transactions on medical imaging* **29,** 650–668 (2009).
29. Heeren, B., Rumpf, M., Schröder, P., Wardetzky, M. & Wirth, B. *Exploring the Geometry of the Space of Shells* 2014.
30. Fröhlich, S. & Botsch, M. *Example-Driven Deformations Based on Discrete Shells* 2011.
31. Budday, S. *et al.* Mechanical properties of gray and white matter brain tissue by indentation. *Journal of the mechanical behavior of biomedical materials* **46,** 318–330 (2015).
32. Amiez, C. *et al.* Sulcal organization in the medial frontal cortex provides insights into primate brain evolution. *Nature communications* **10,** 1–14 (2019).
33. Friedrich, P. *et al.* Imaging evolution of the primate brain: the next frontier? en. *Neuroimage* **228,** 117685 (Mar. 2021).
34. Solomon, J. *et al. Convolutional wasserstein distances* 2015.
35. Glasser, M. F. *et al.* A multi-modal parcellation of human cerebral cortex. *Nature* **536,** 171–178 (2016).
36. Kaas, J. H. The evolution of brains from early mammals to humans. en. *Wiley Interdiscip. Rev. Cogn. Sci.* **4,** 33–45 (Jan. 2013).
37. Krubitzer, L. A. & Prescott, T. J. The Combinatorial Creature: Cortical Phenotypes within and across Lifetimes. en. *Trends Neurosci.* **41,** 744–762 (Oct. 2018).
38. Paxinos, G. & Watson, C. *The Rat Brain in Stereotaxic Coordinates: Hard Cover Edition* en (Elsevier, Nov. 2006).

39. Paxinos, G. & Franklin, K. B. J. *Paxinos and Franklin's the Mouse Brain in Stereotaxic Coordinates* en (Academic Press, Apr. 2019).

40. Paxinos, G., Petrides, M. & Evrard, H. C. *The Rhesus Monkey Brain in Stereotaxic Coordinates* en (Elsevier Science, Nov. 2021).

41. Paxinos, G., Watson, C., Petrides, M., Rosa, M. & Tokuno, H. *The Marmoset Brain in Stereotaxic Coordinates* en (Elsevier Science, Oct. 2011).

42. Amunts, K., Mohlberg, H., Bludau, S. & Zilles, K. Julich-Brain: A 3D probabilistic atlas of the human brain's cytoarchitecture. *Science* **369,** 988–992 (2020).

43. Striedter, G. F., Bullock, T. H., Preuss, T. M., Rubenstein, J. & Krubitzer, L. A. *Evolution of Nervous Systems* en (Elsevier Science, Dec. 2006).

44. Nelder, J. A. & Mead, R. A simplex method for function minimization. *The computer journal* **7,** 308–313 (1965).
