# Supplementary material for: Evolution of cortical geometry and its link to function, behaviour and ecology
Source: Nat Commun. 2023 Apr 20;14:2252. doi: 10.1038/s41467-023-37574-x (PMC10119184; doi:10.1038/s41467-023-37574-x)
Supplement: Supplementary file 3 — Description of Additional Supplementary Files [file 41467_2023_37574_MOESM3_ESM.pdf]

Description of Additional Supplementary Files

File Name: Supplementary Data 1
Description: a: Provenience and references of used data. b: Provenience and references of reference endocasts (Figure 1)

File Name: Supplementary Data 2
Description: a: Behavioral and Ecological variables and their discrete encodings. b: References for behavioral and ecological variables

File Name: Supplementary Data 3
Description: Correlation values in meta-analytic decoding of relative expansion maps associated with sociobehavioral variables

File Name: Supplementary Data 4
Description: a: Statistical Information Figure 4a. b: Statistical Information for Figure 4c. c: Statistical Information for Figure 4c

File Name: Supplementary Data 5
Description: Statistical Information for Extended Data Figure 5

File Name: Supplementary Data 6
Description: a: Statistical Information for Figure 6c, Extended Data Figure 6. b: Statistical Information for Figure 6b

File Name: Supplementary Data 7
Description: a: Meta-analytic decoding of estimated cortical expansion in the human lineage. b: Repeated Measurements ANOVA of correlations between statistical maps of neuroscientific terms and cortical expansion in the lineage of Homo. c: Statistical Information Figure 7. d: Licensing information for artwork used in Figure 7

File Name: Supplementary Data 8
Description: a: Statistical Information for Supplementary Figure 4a. b: Statistical Information for Supplementary Figure 4d

File Name: Supplementary Data 9
Description: a: Correlation between progression of meta-analytical term decodings of evolutionary cortical surface expansion and estimates of ancestral likelihoods of socioecological factors. b: Partial correlation values between evolutionary cortical expansion maps decoded into metaanalytical terms and estimated likelihood of social life-style, controlled for diurnality

File Name: Supplementary Data 10
Description: a: Statistical Information for Supplementary Figure 2c. b: Statistical Information for Supplementary Figure 2d

File Name: Supplementary Data 11
Description: Overview of all image data and corresponding cortical surfaces used in the study.
