# Supplementary material for: Evolution of cortical geometry and its link to function, behaviour and ecology
Source: Nat Commun. 2023 Apr 20;14:2252. doi: 10.1038/s41467-023-37574-x (PMC10119184; doi:10.1038/s41467-023-37574-x)
Supplement: Supplementary file 14 — Supplementary Data 11 [file 41467_2023_37574_MOESM14_ESM.pdf]

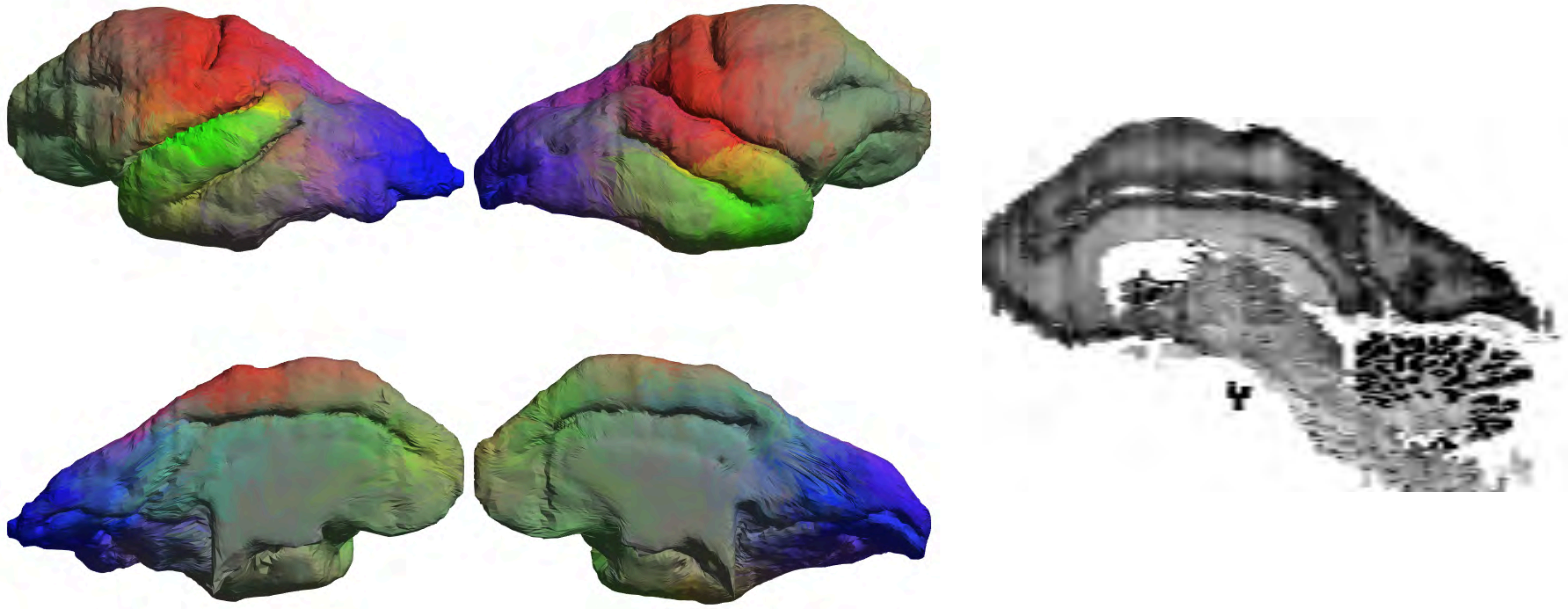

Alouatta palliata

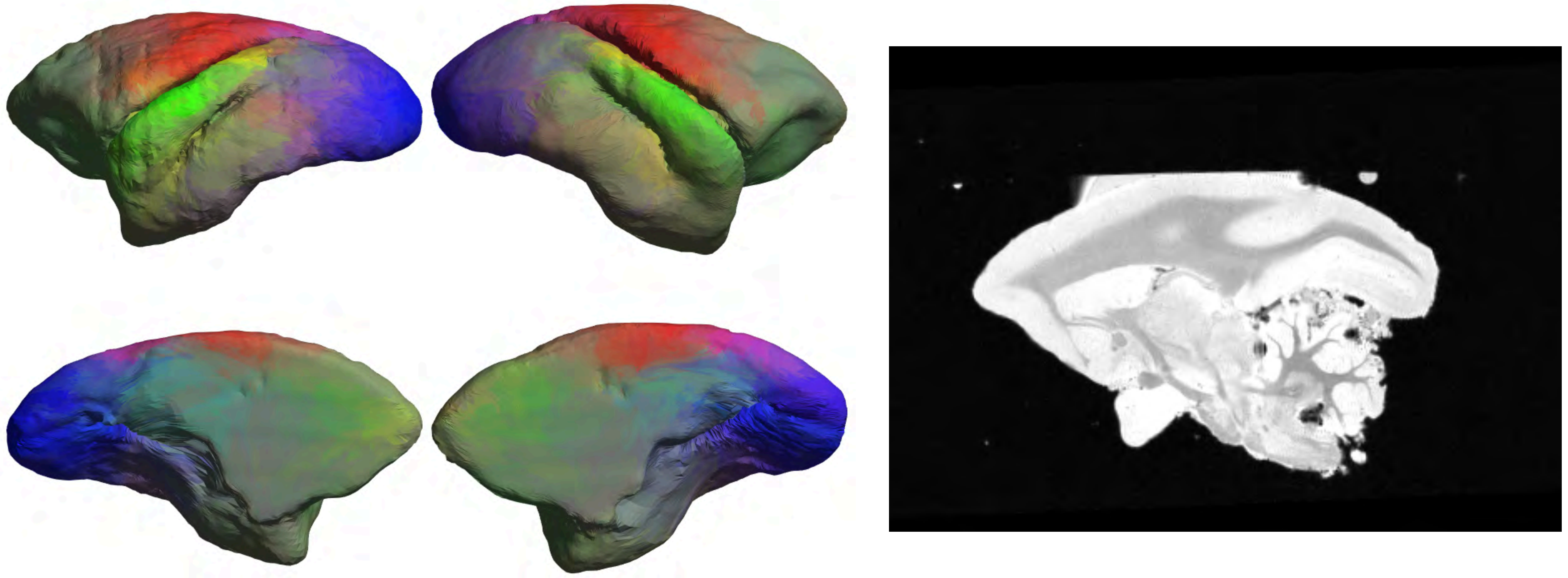

Aotus lemurinus

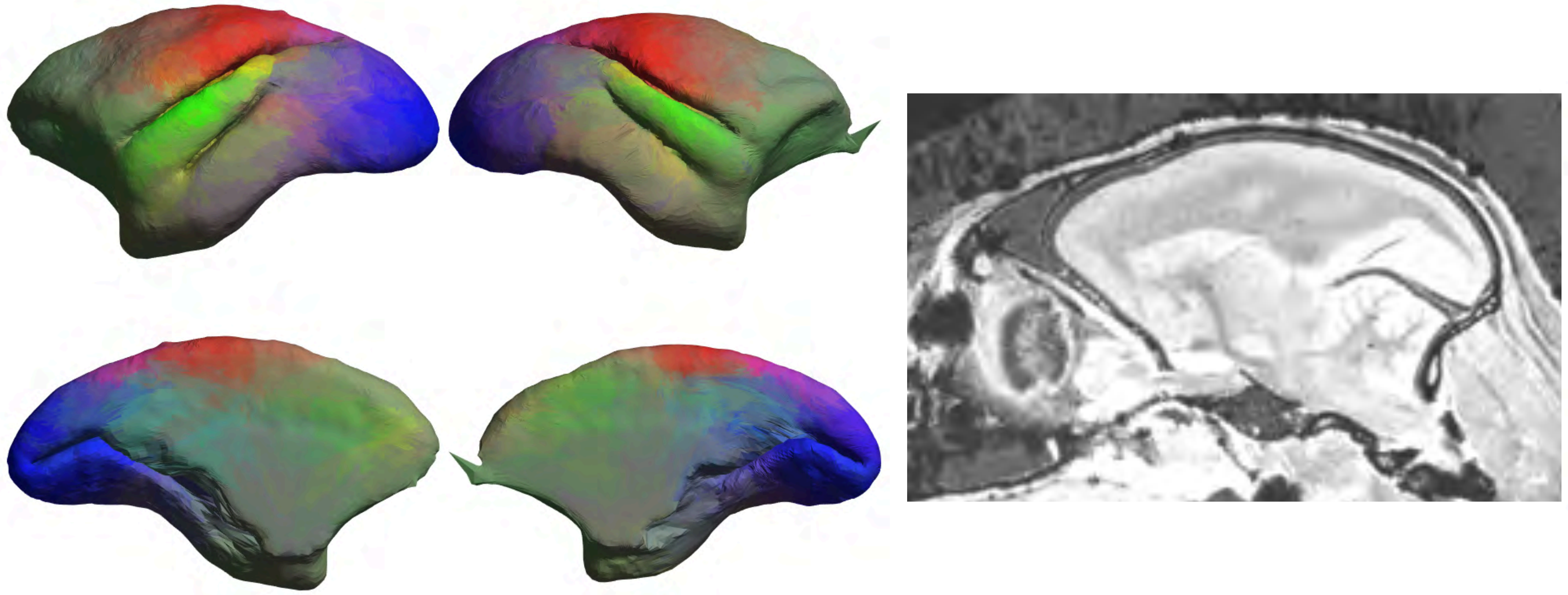

Aotus trivirgatus

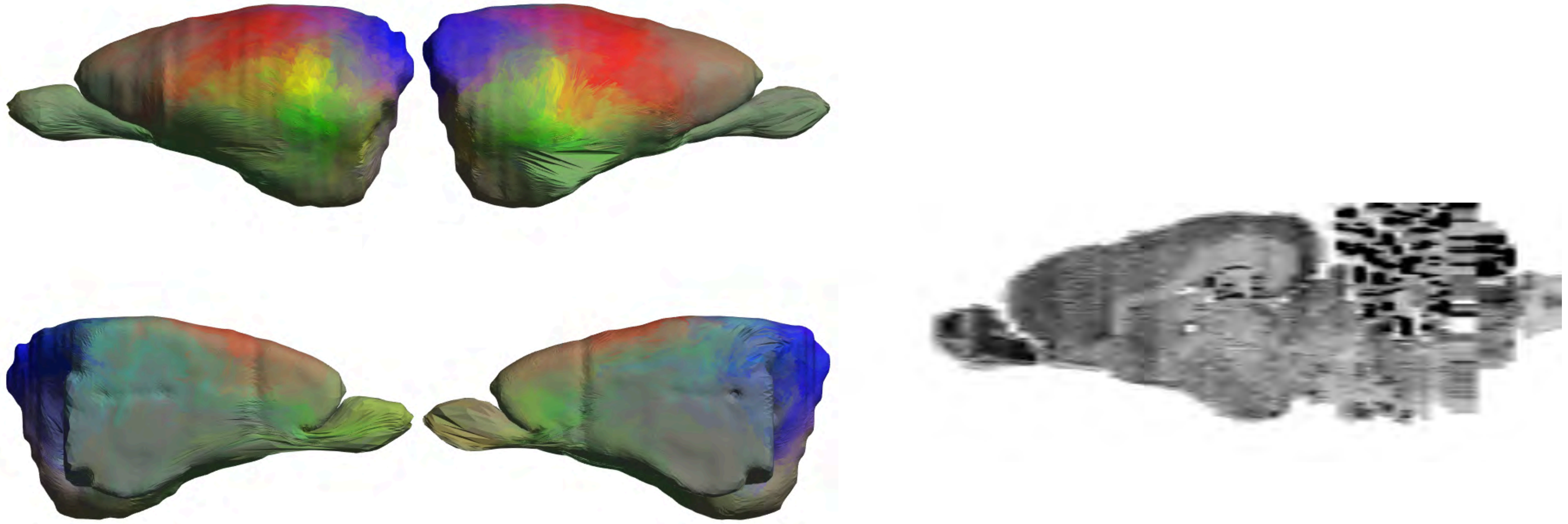

Aplodontia rufa

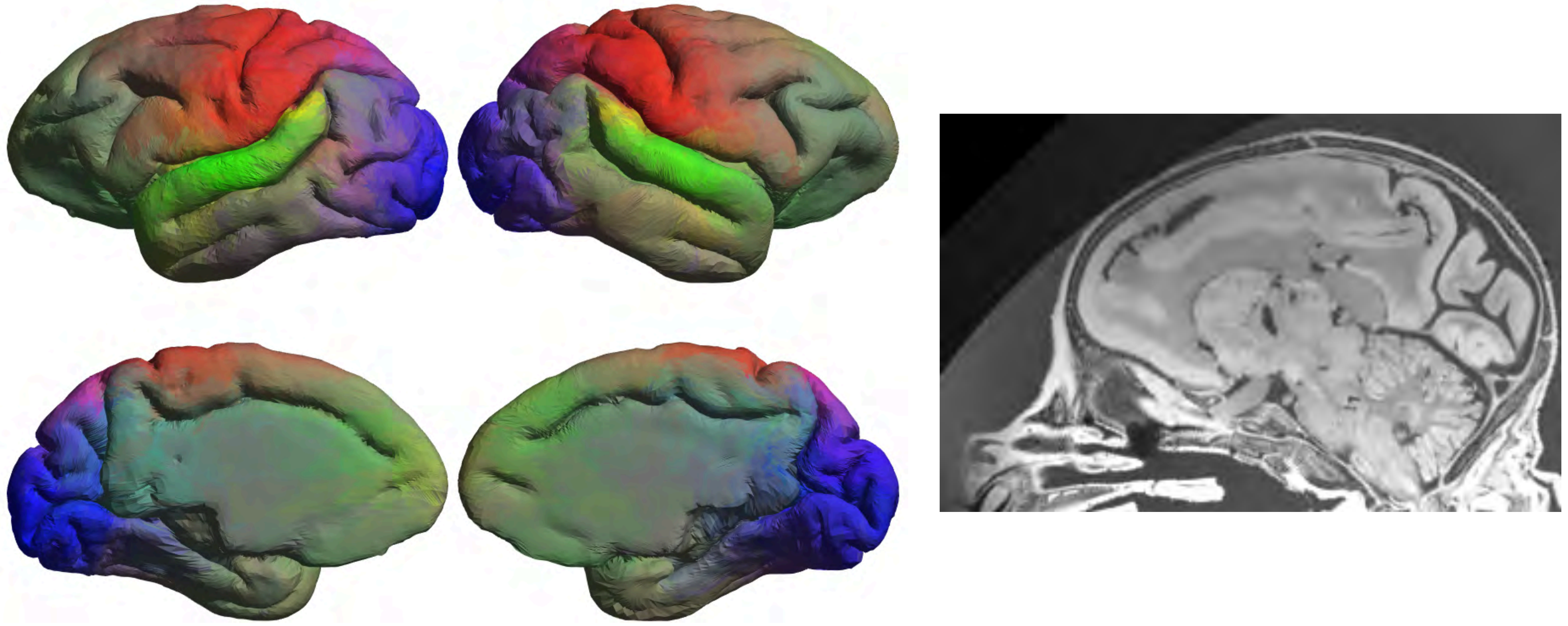

Ateles paniscus

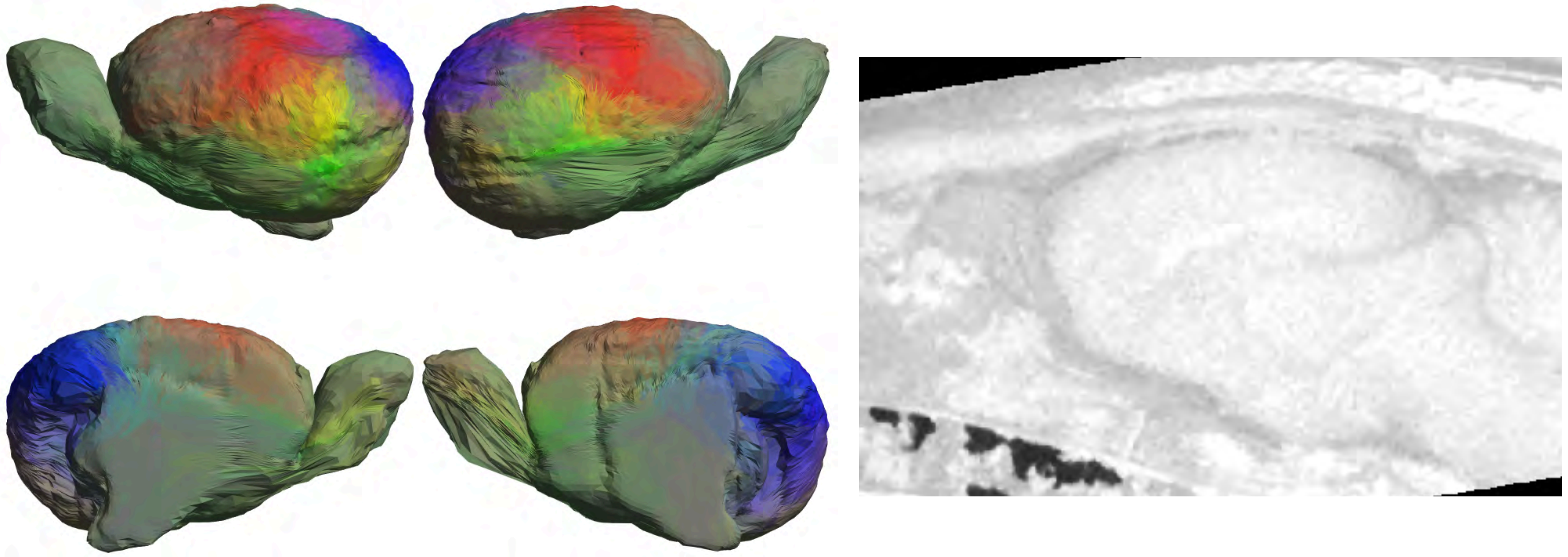

Bathyergus suillus

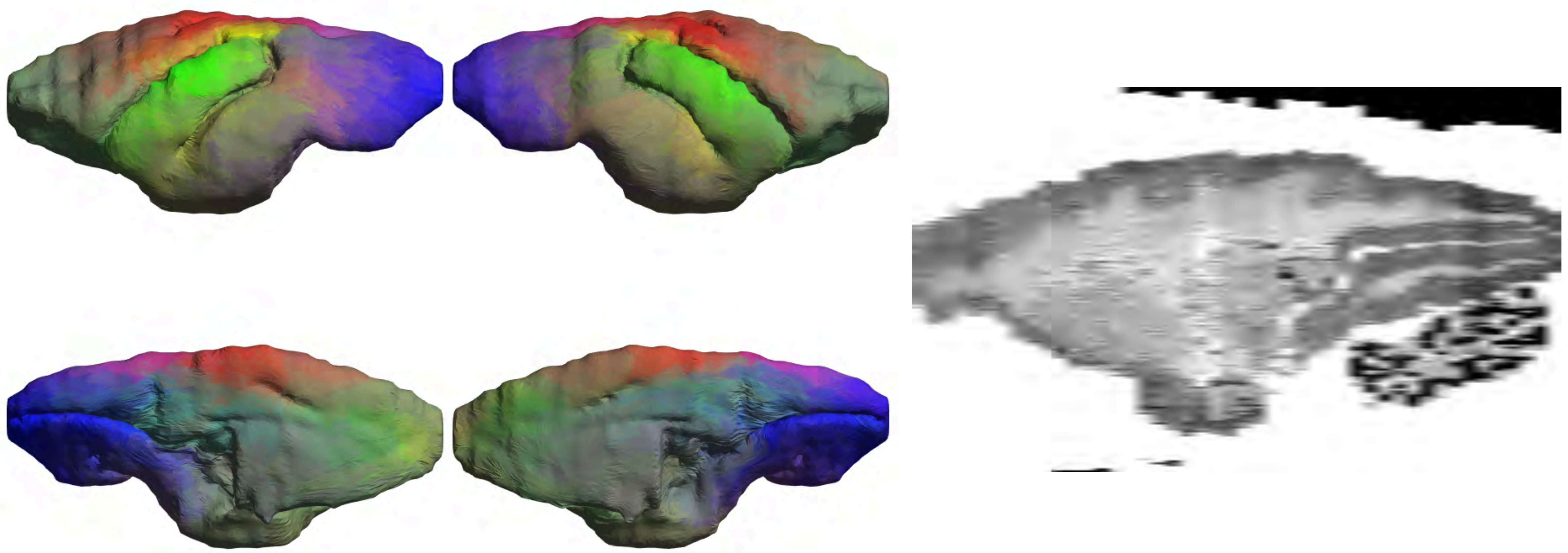

Callicebus moloch

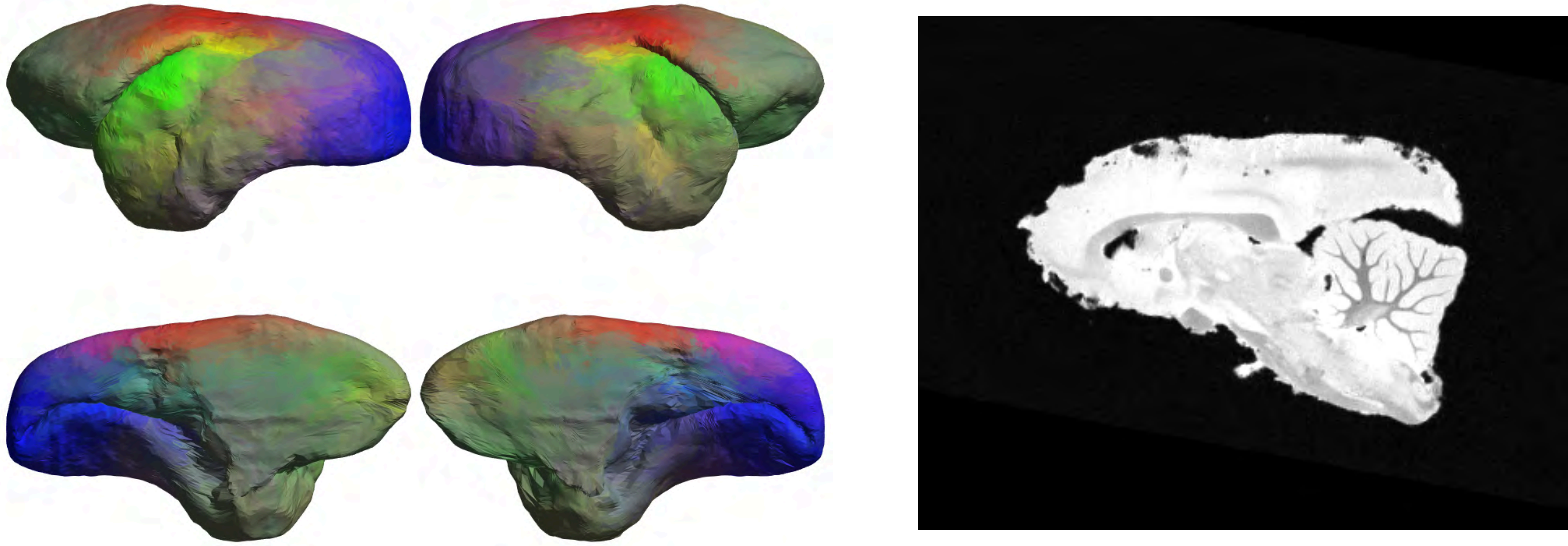

Callimico goeldii

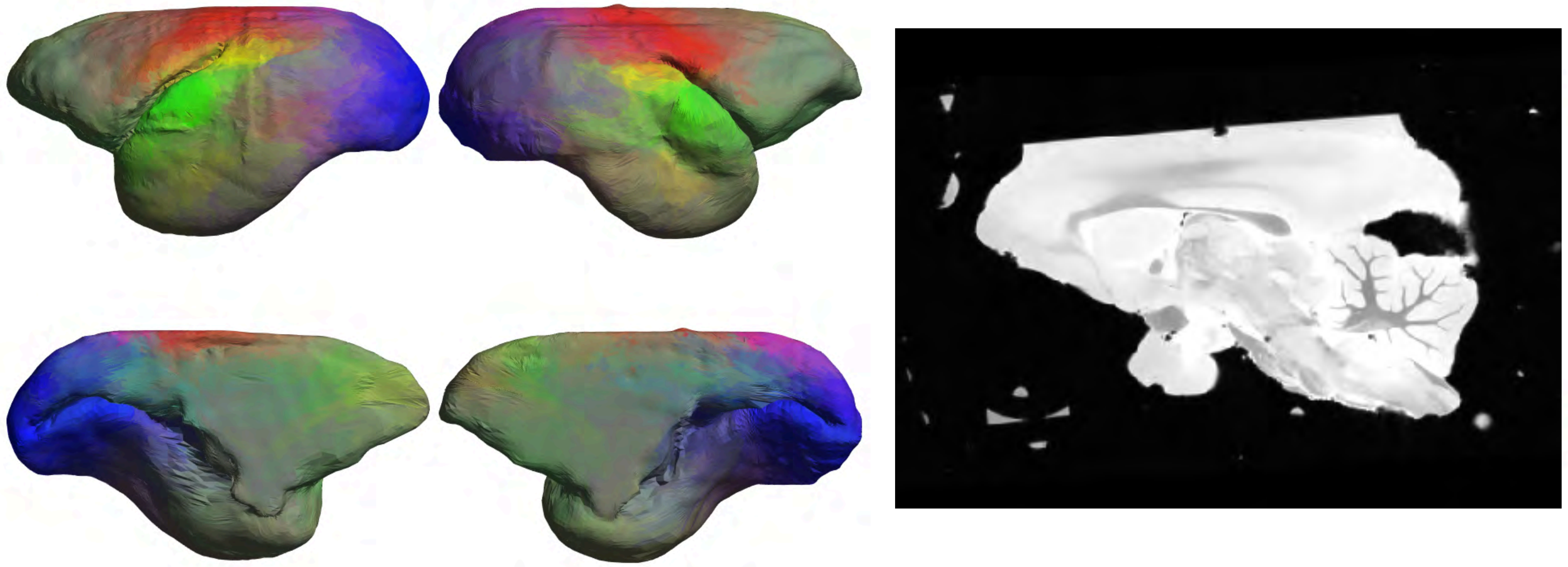

Callithrix geoffroyi

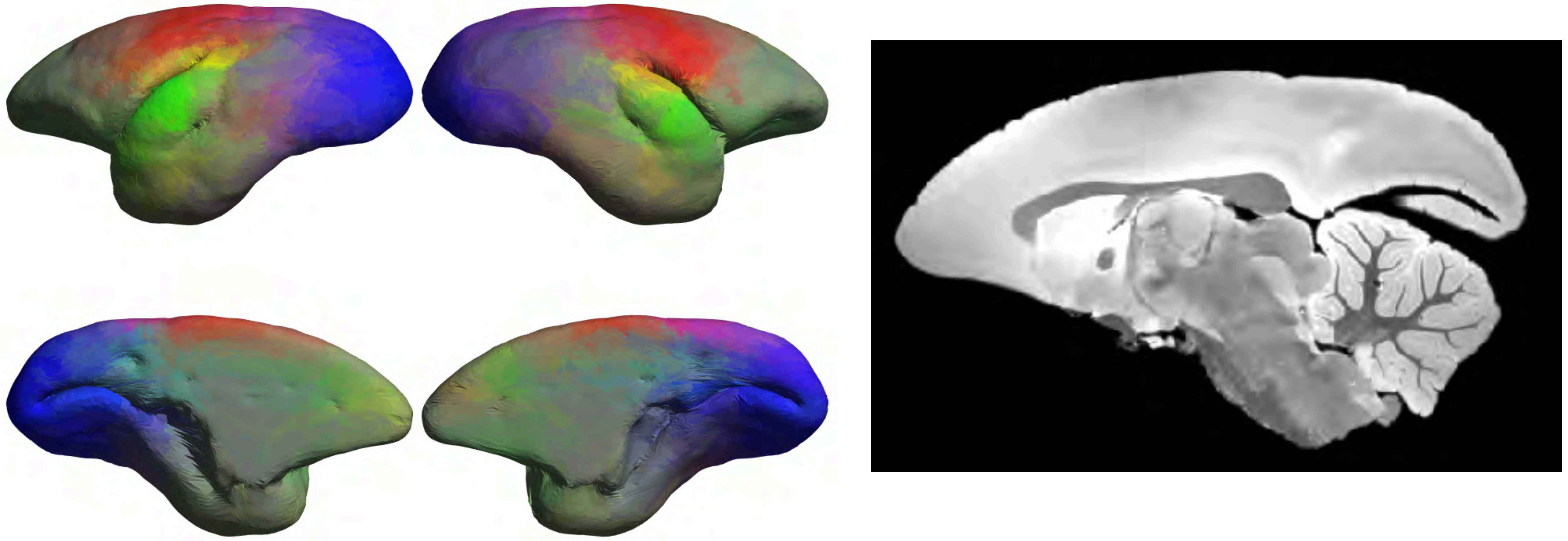

Callithrix jacchus

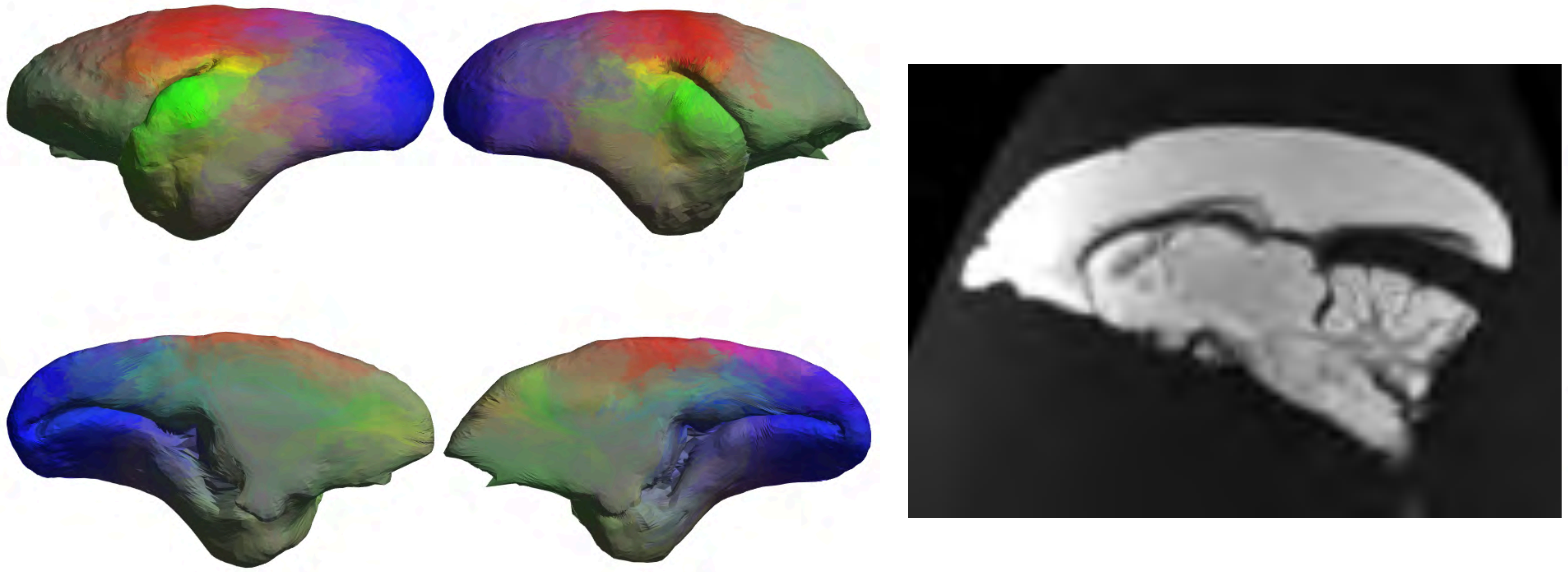

Callithrix penicillata

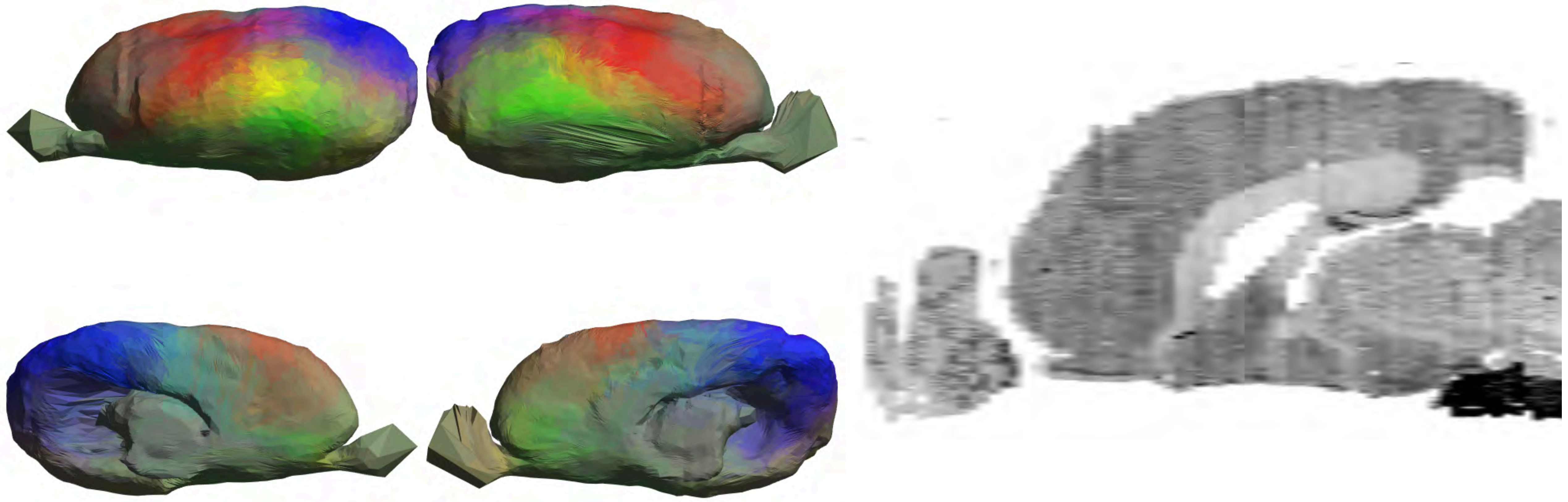

Castor canadensis

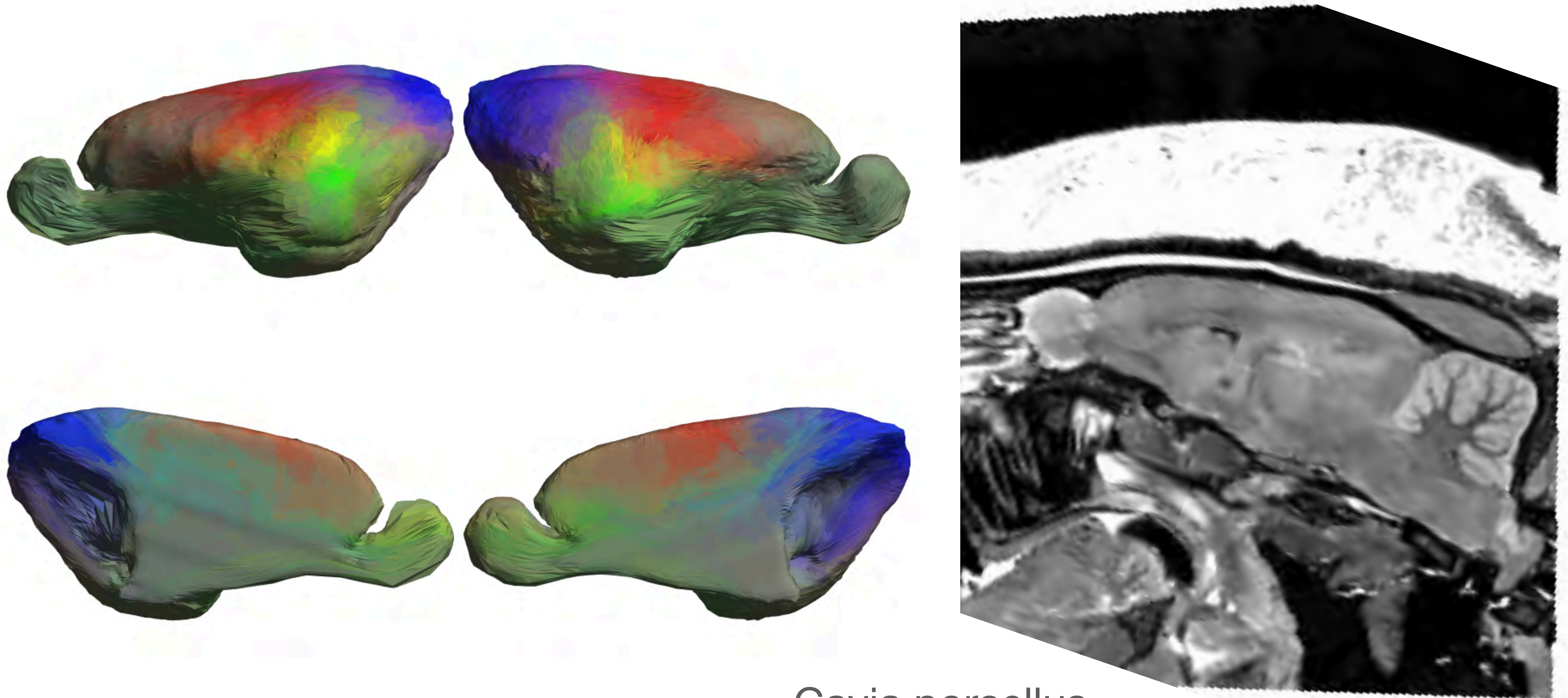

Cavia porcellus

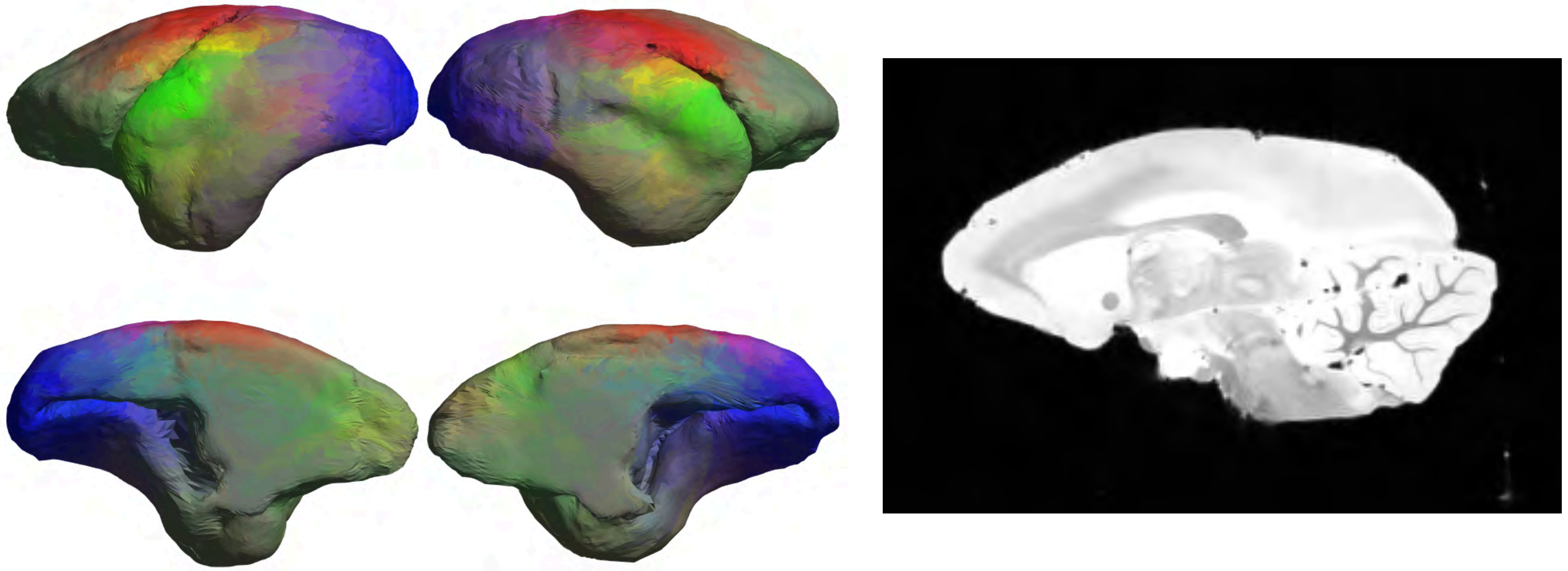

Cebuella pygmaea

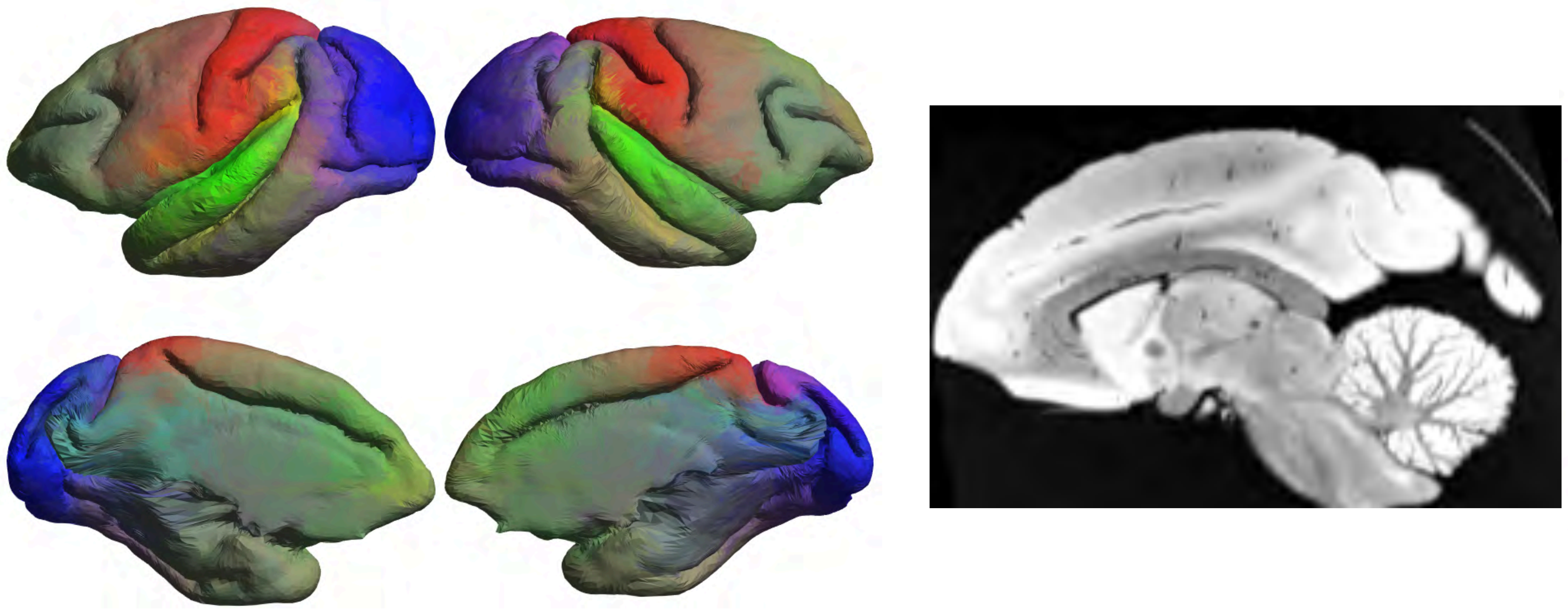

Cebus capucinus

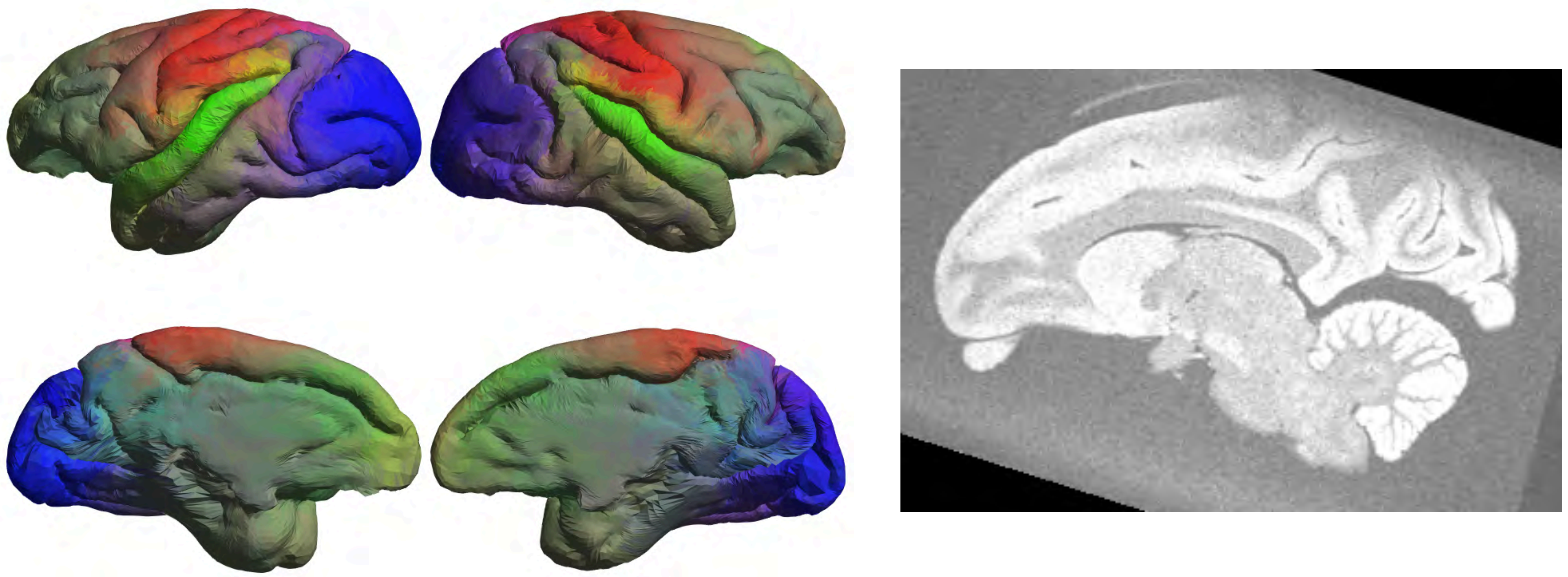

Cercocebus atys

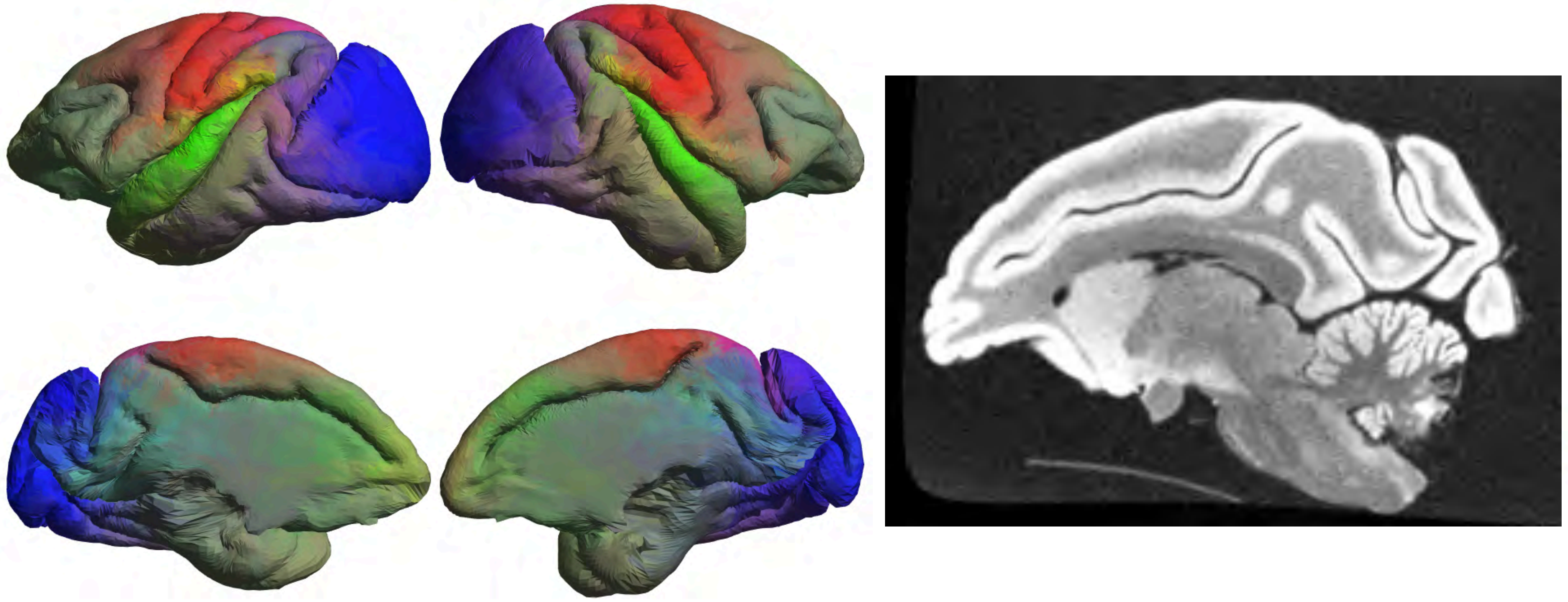

Cercopithecus cephus

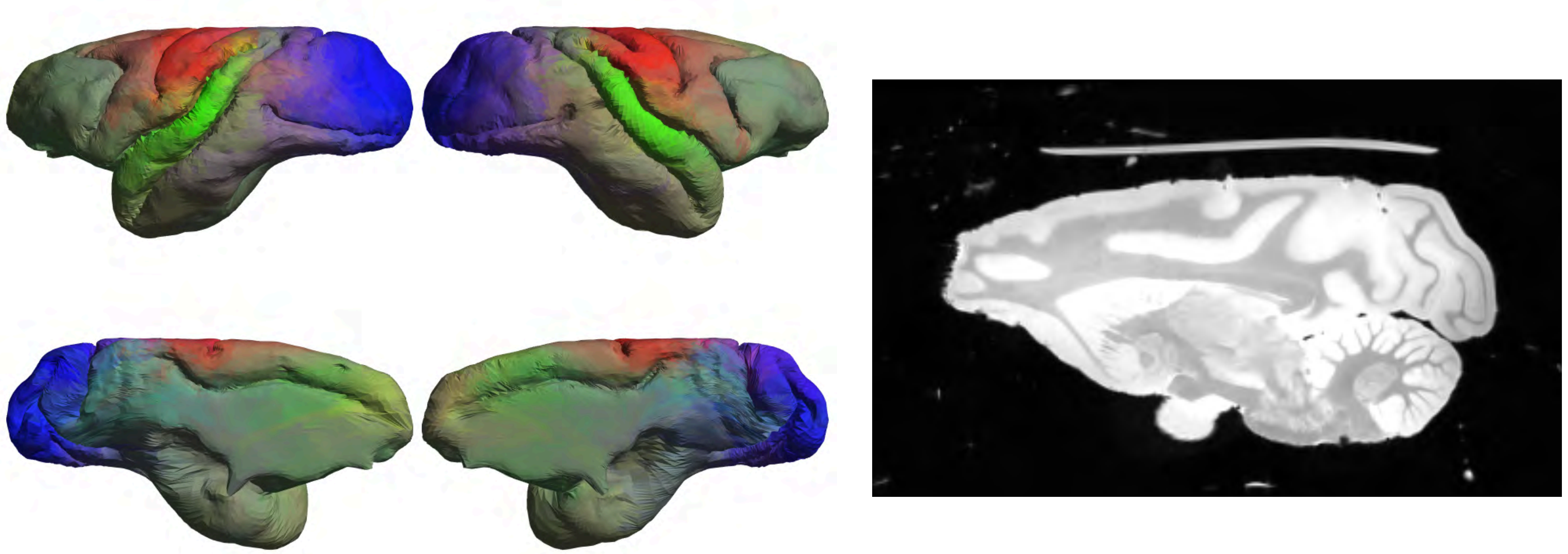

Cercopithecus mitis

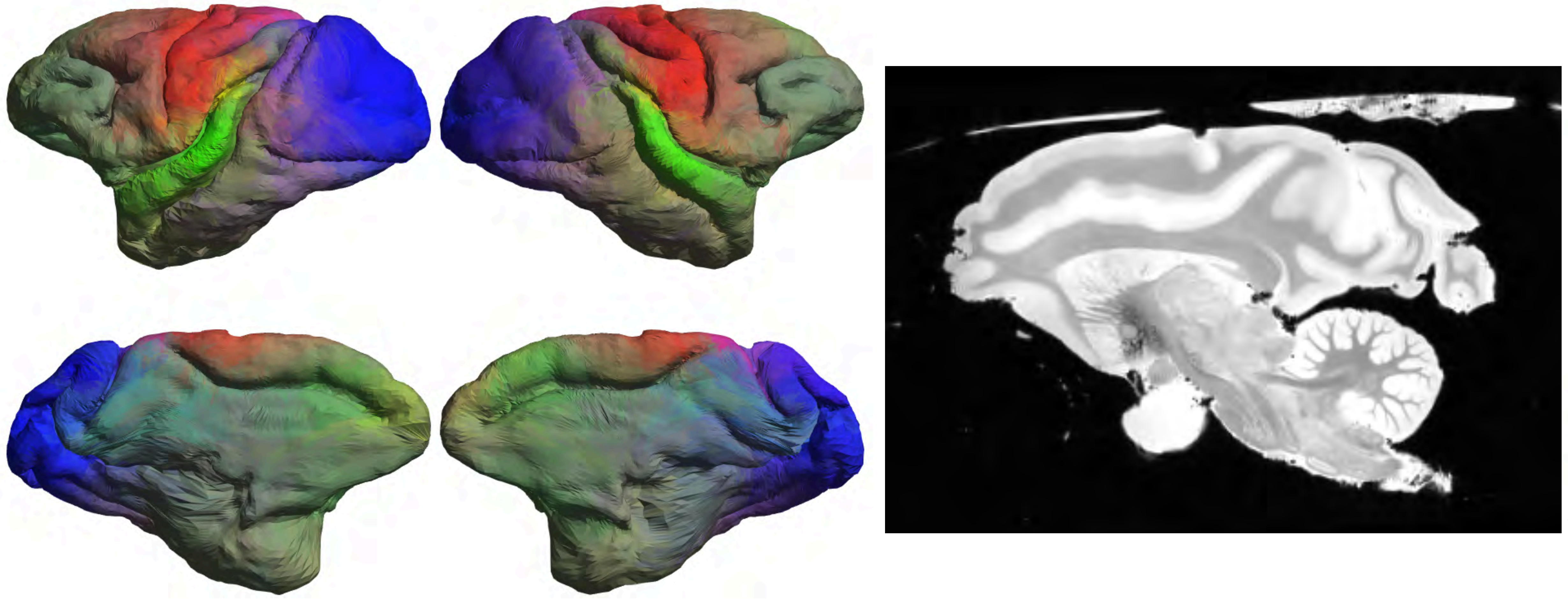

Cercopithecus nictitans

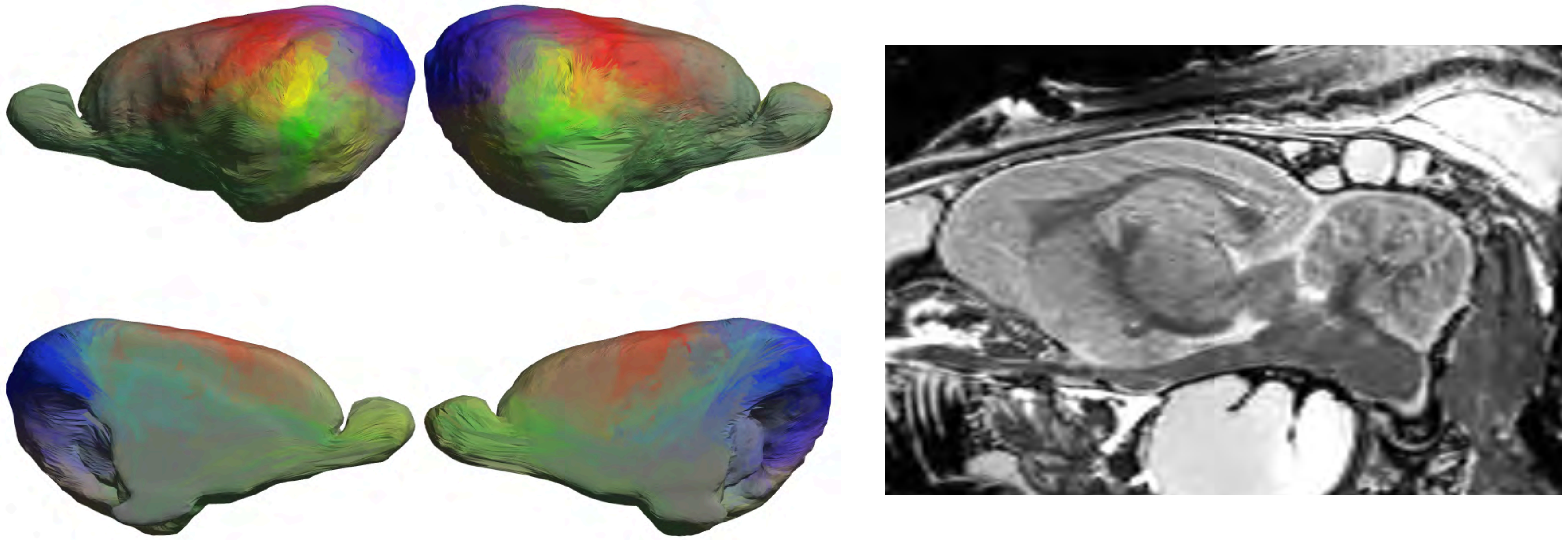

Chinchilla lanigera

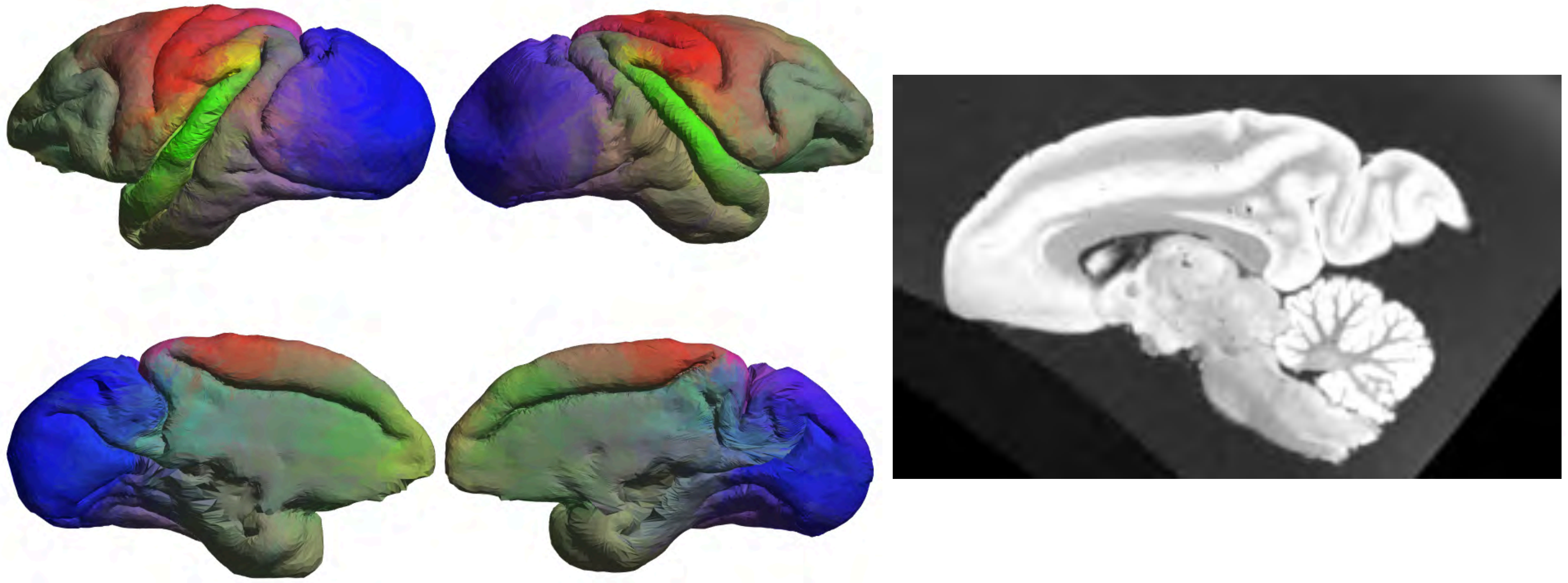

Chlorocebus sabaeus

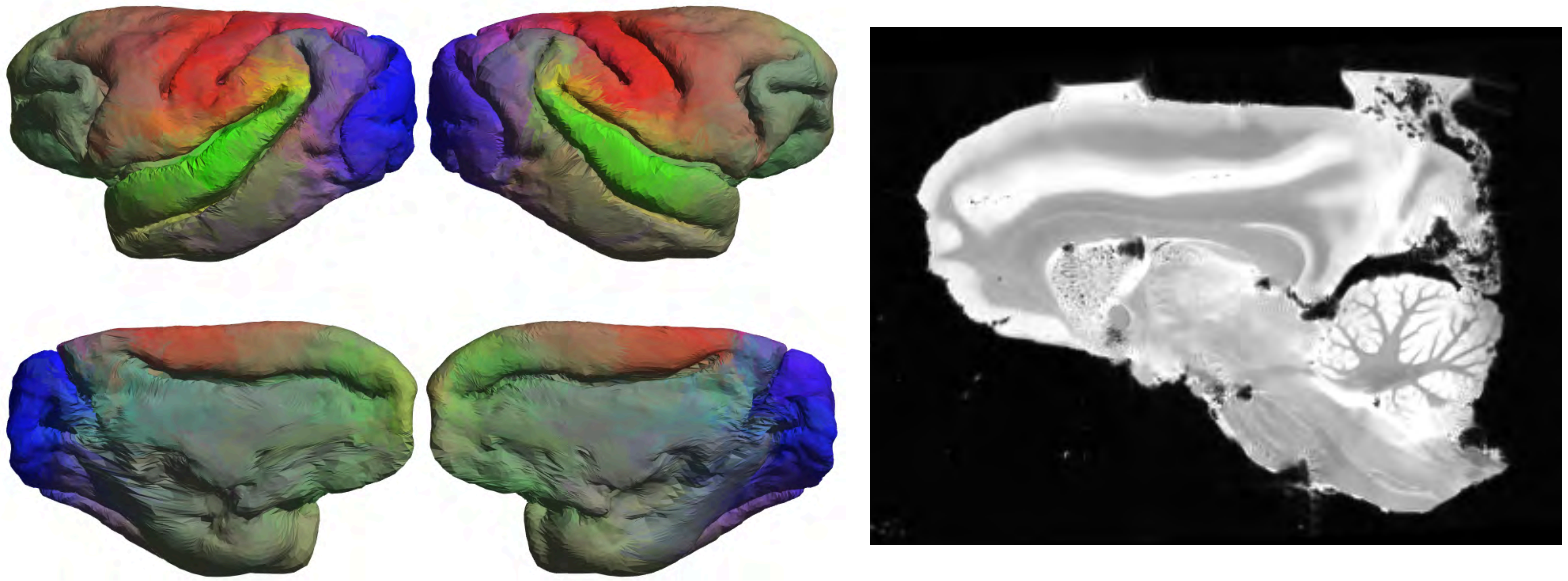

Colobus guereza

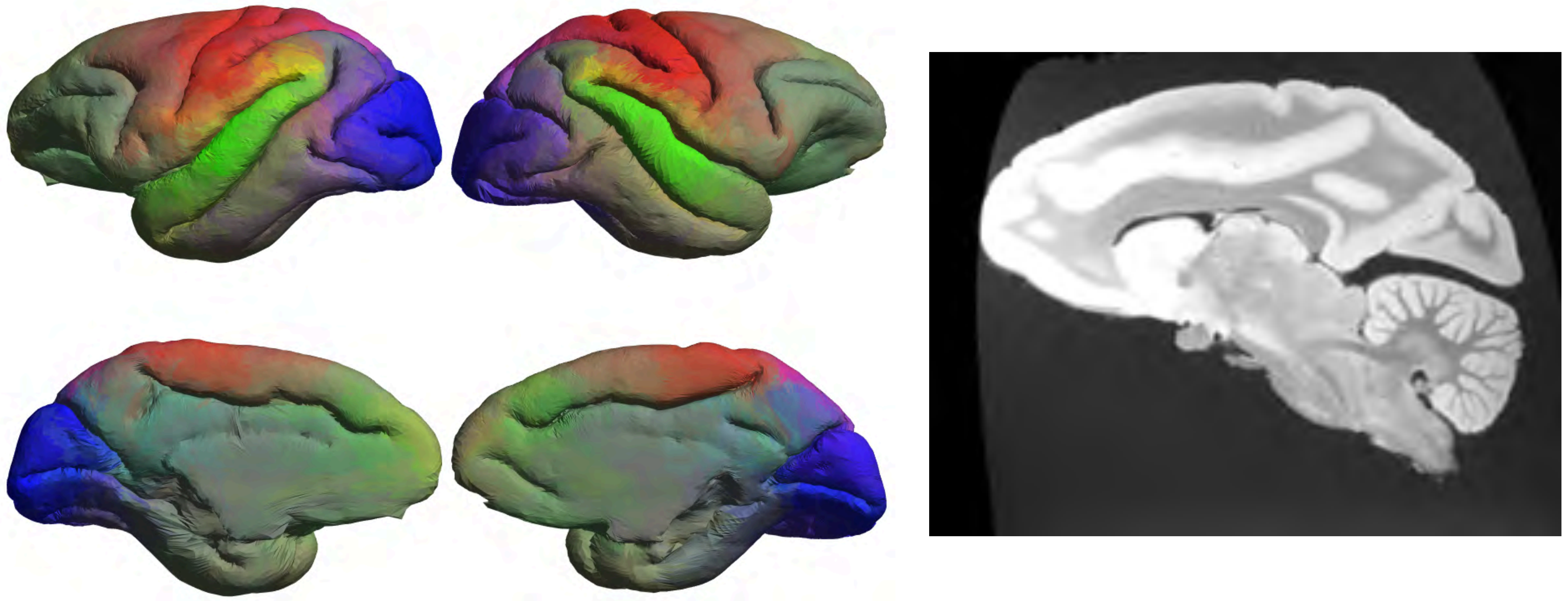

Colobus polykomos

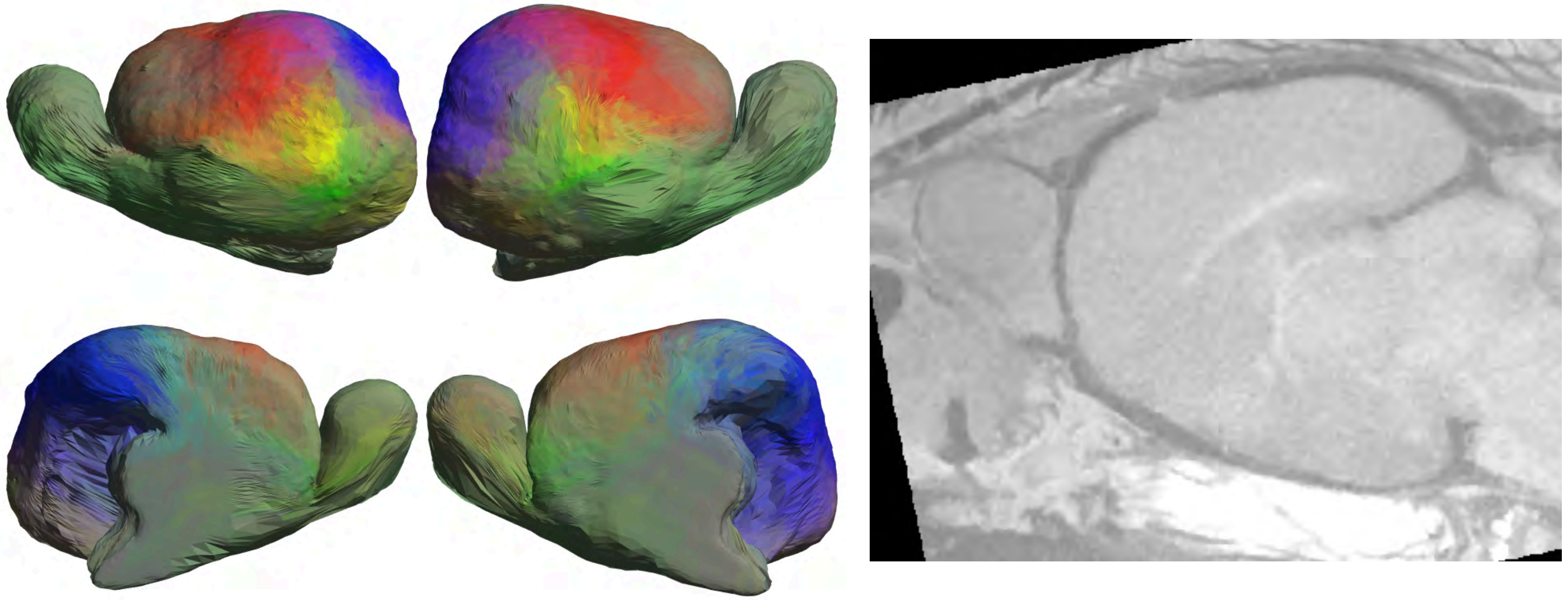

Cryptomys hottentotus

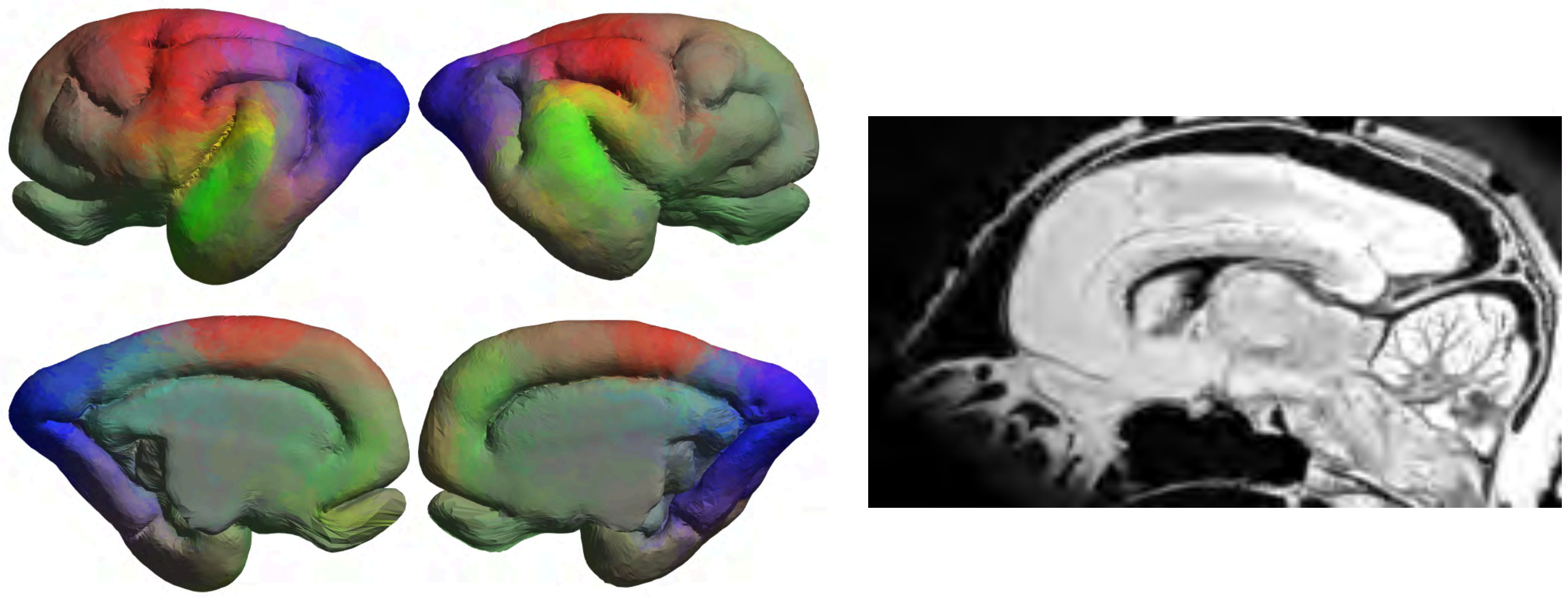

Daubentonia madagascariensis

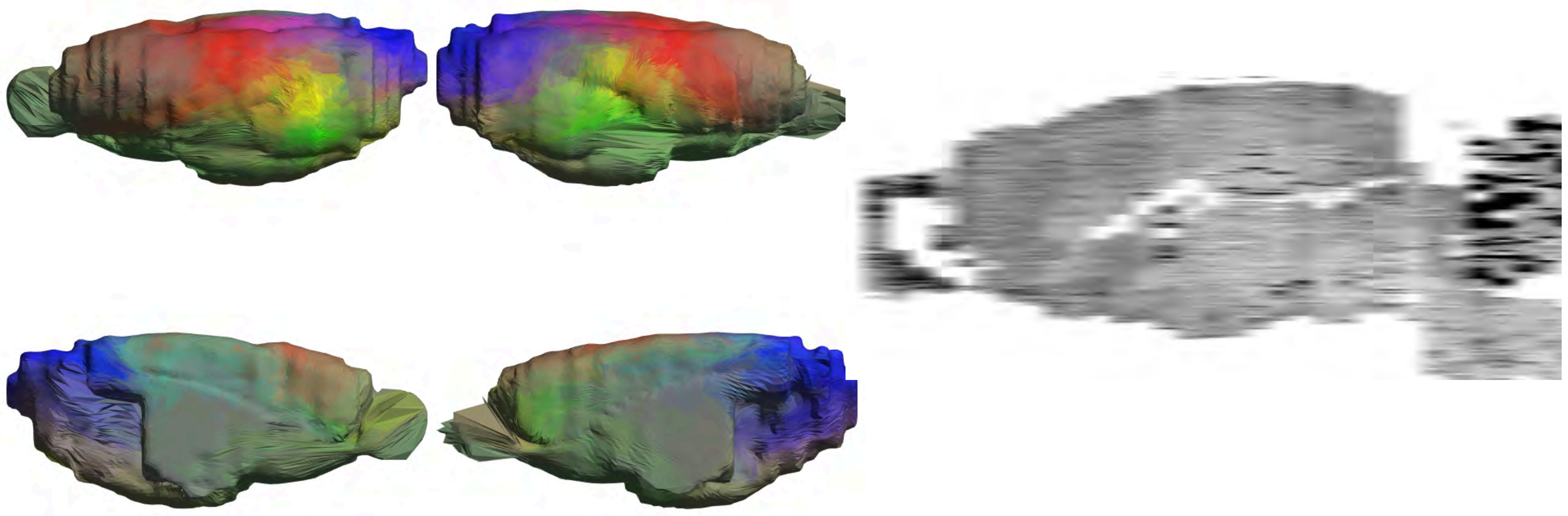

Erethizon dorsatum

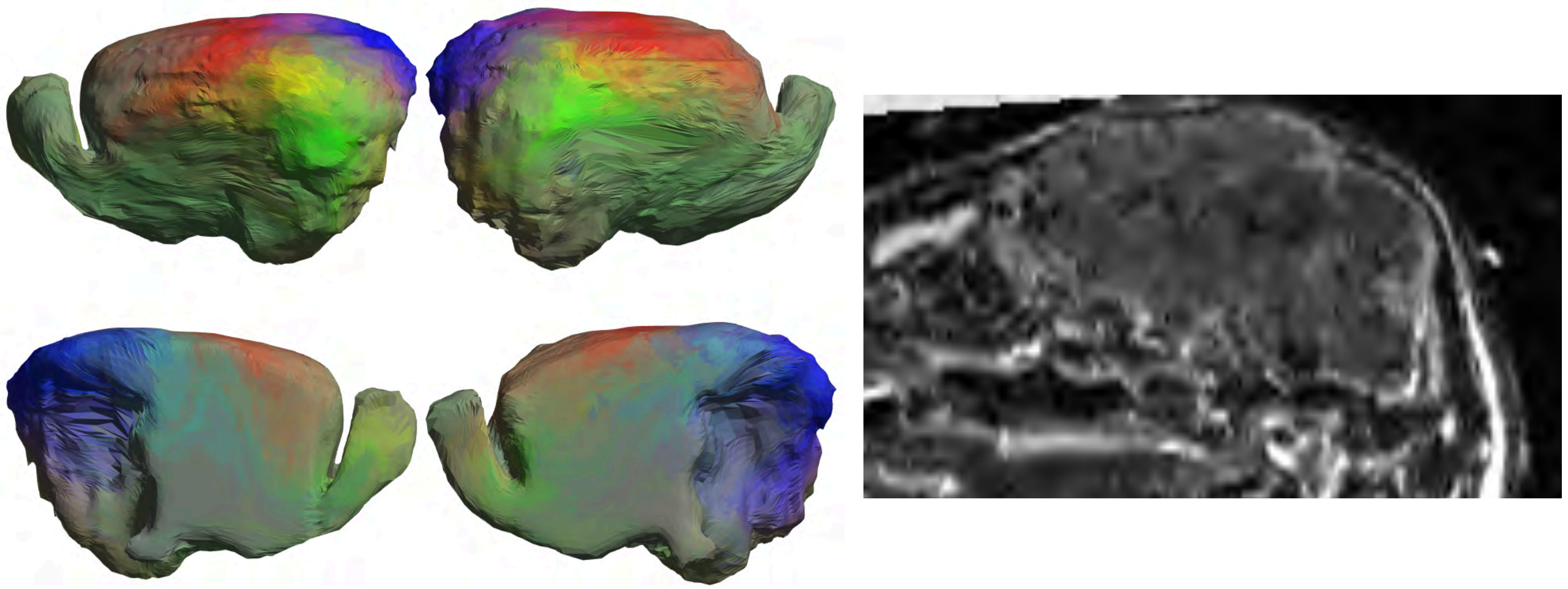

Euchoreutes naso

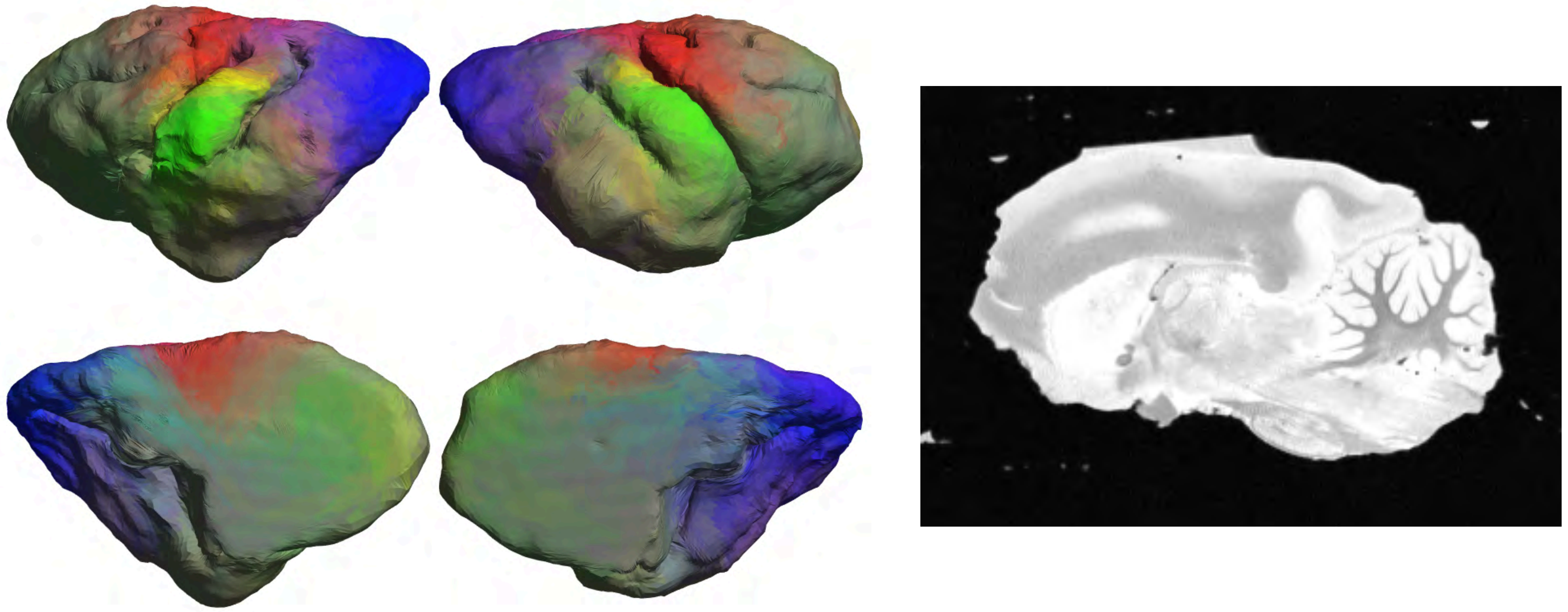

Eulemur coronatus

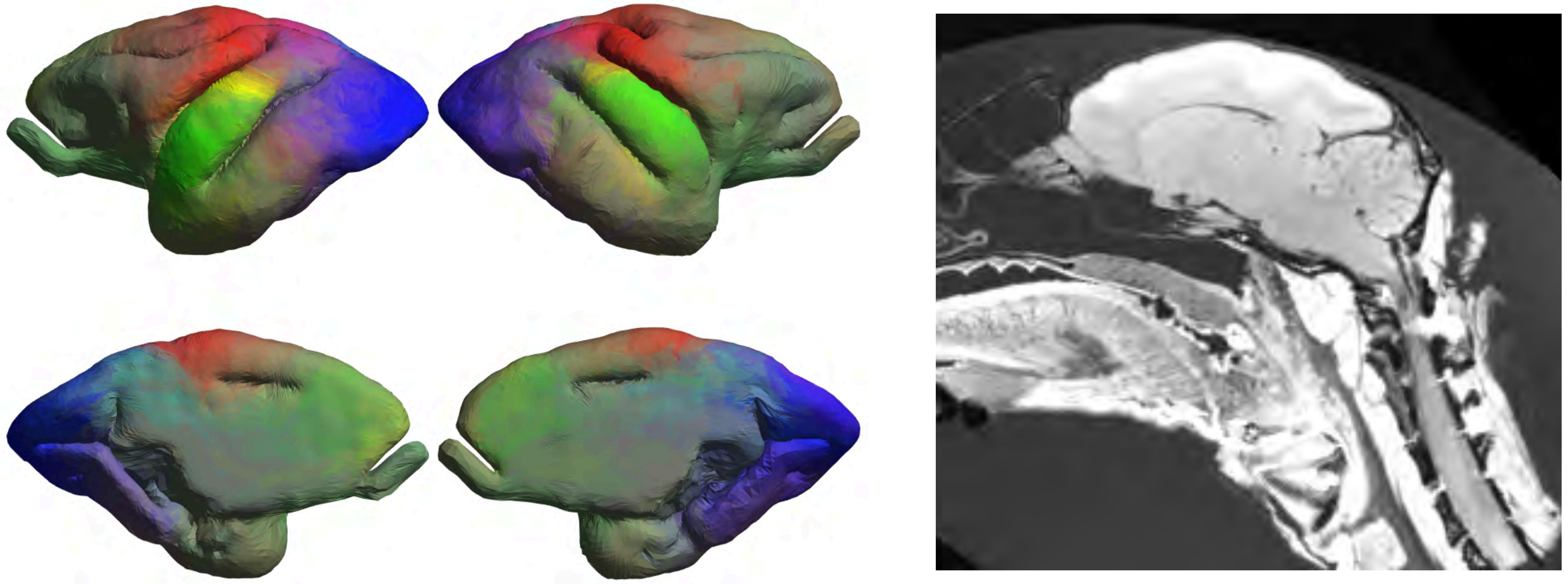

Eulemur mongoz

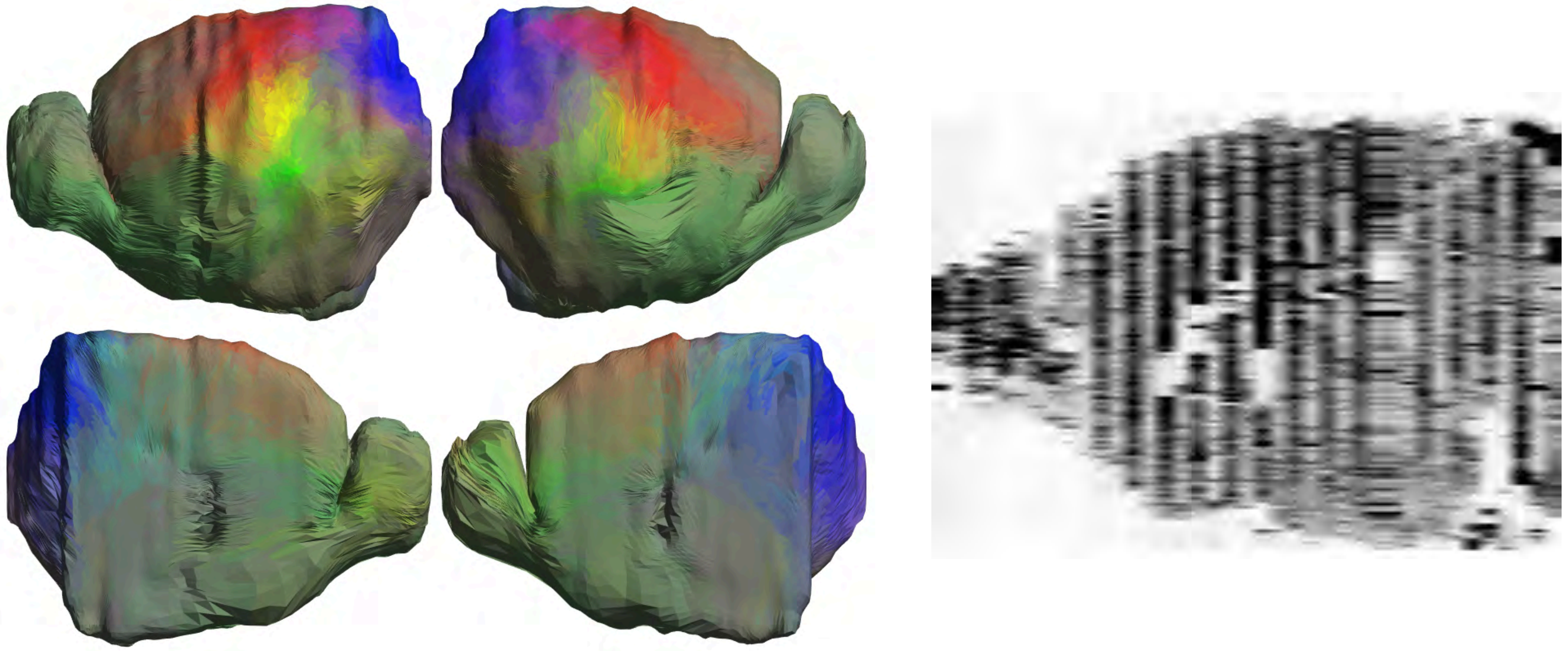

Fukomys anselli

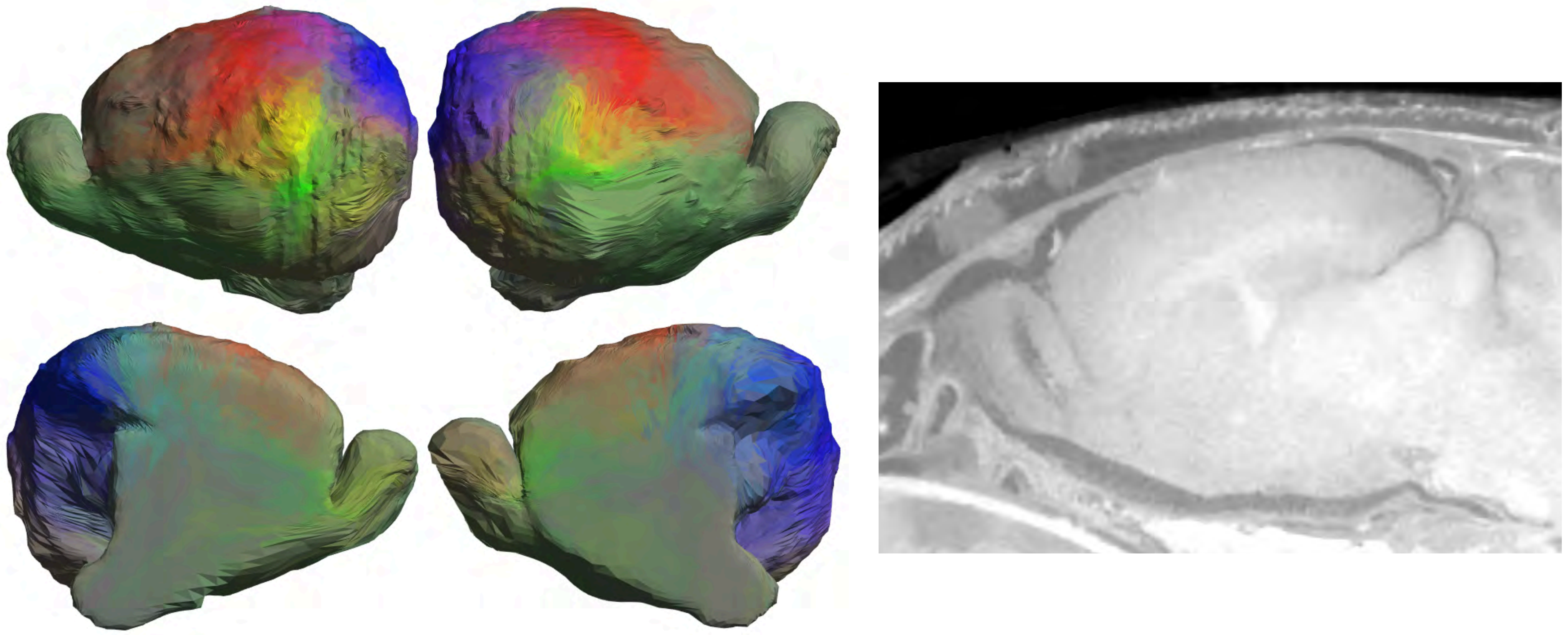

Fukomys mechowi

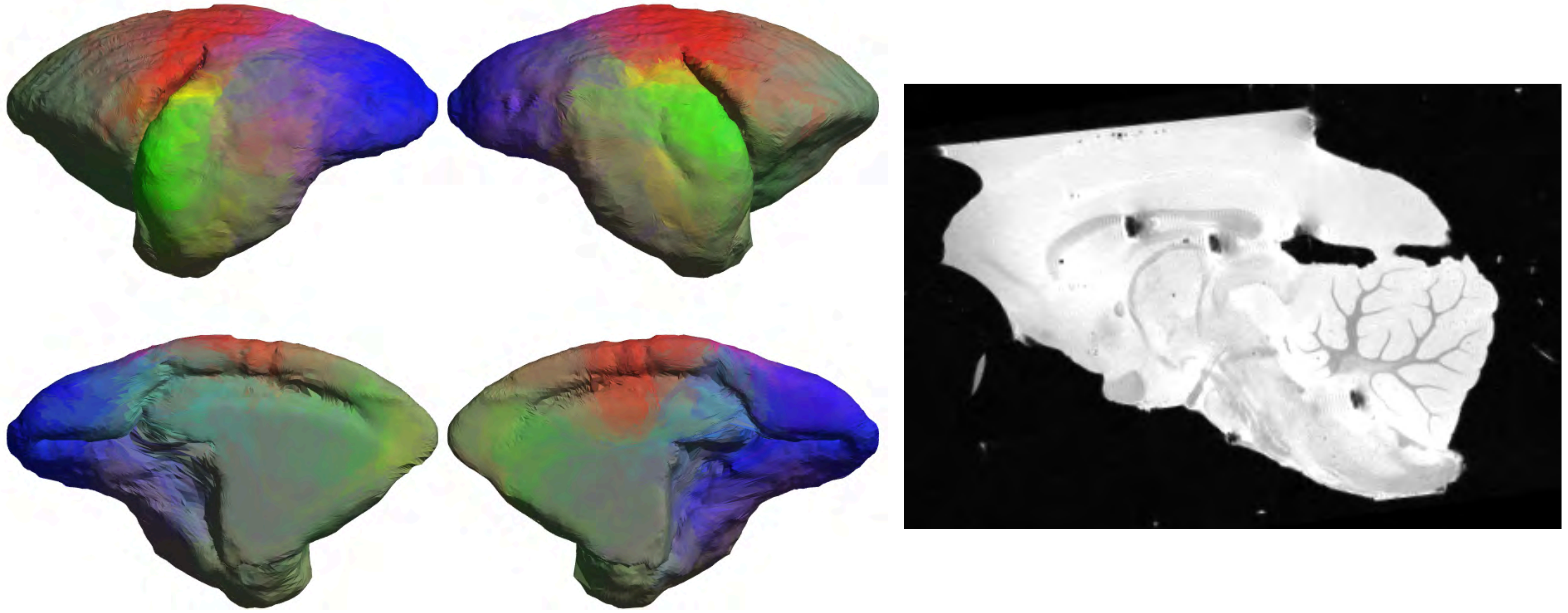

Galago senegalensis

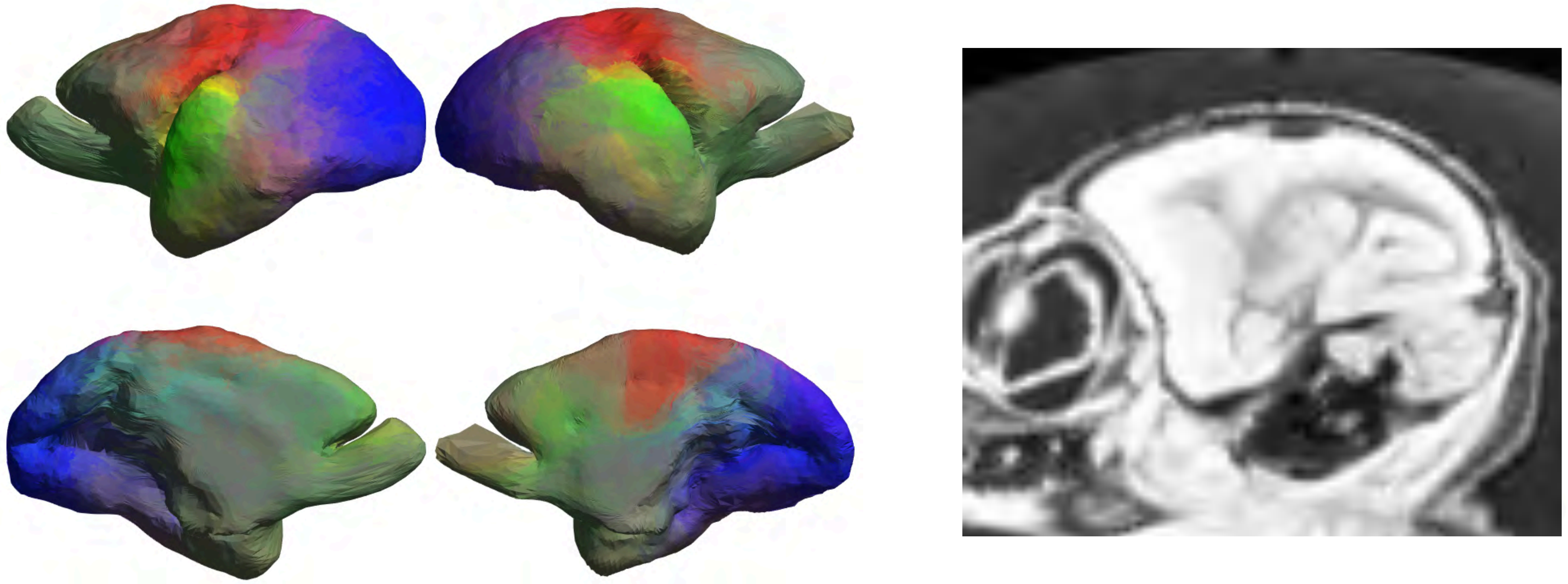

Galagoides demidovii

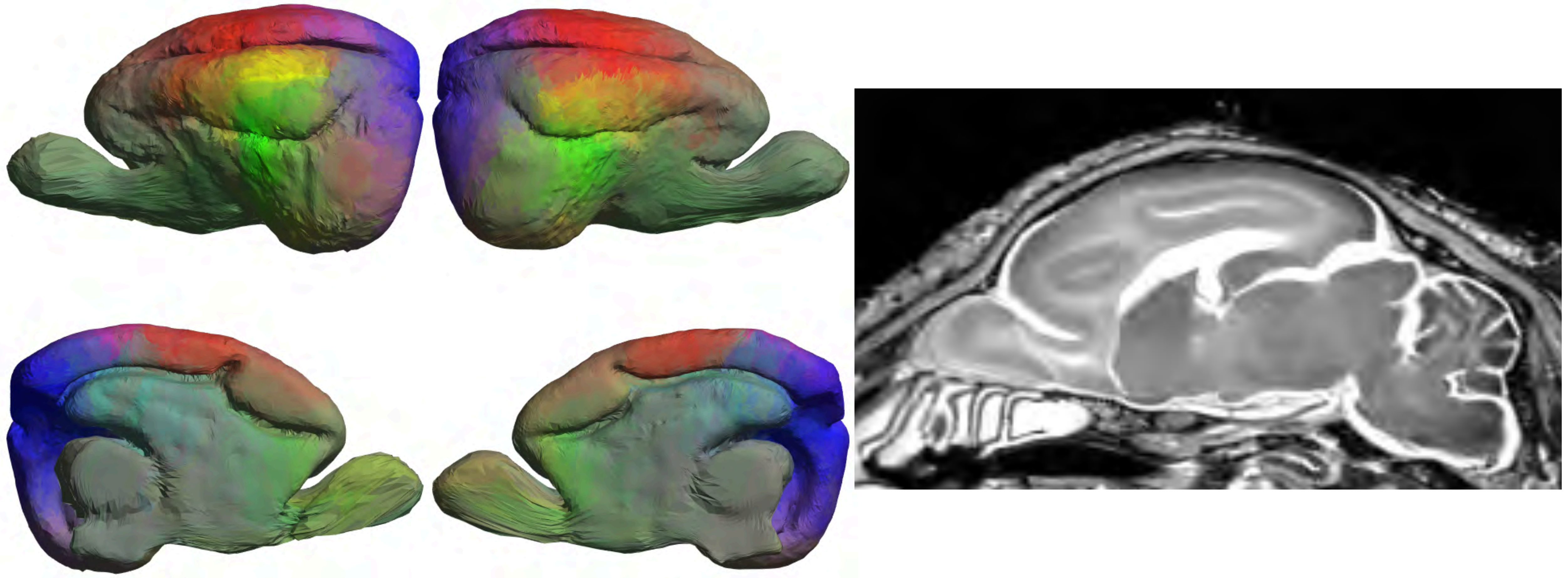

Galeopterus variegatus

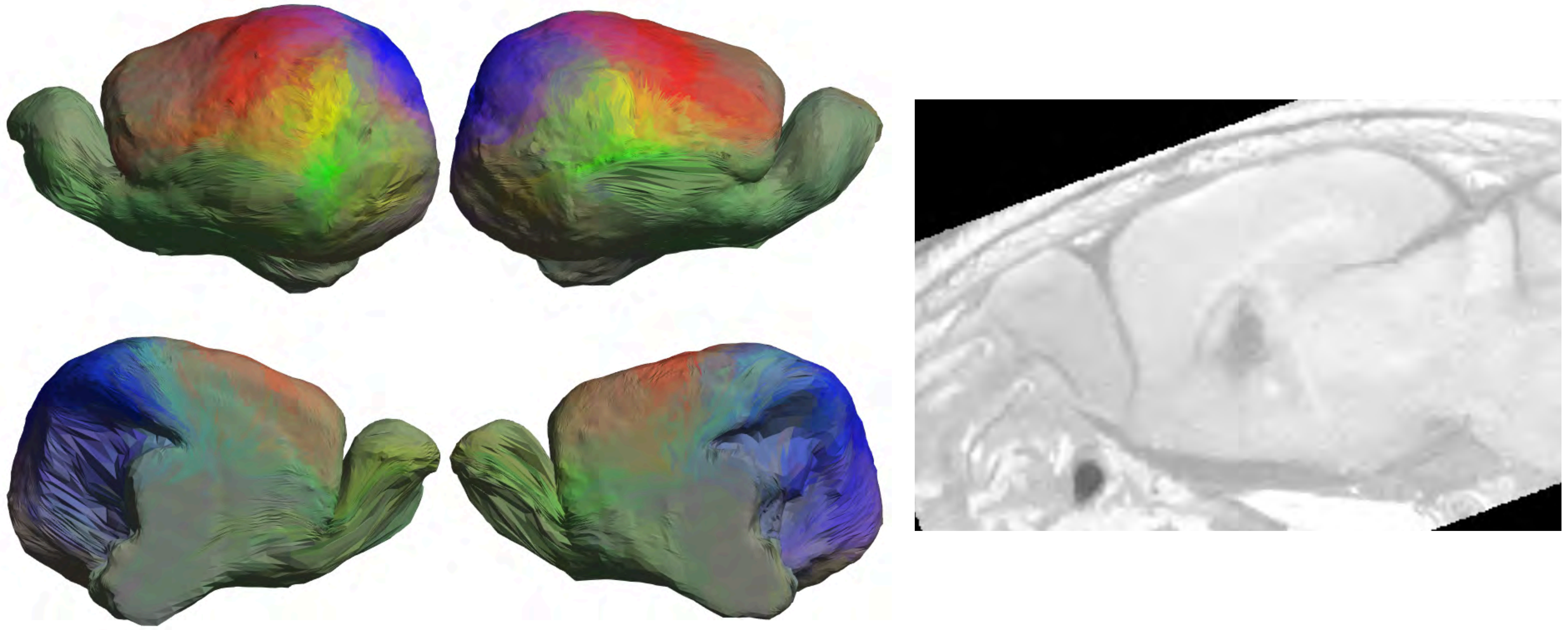

Georychus capensis

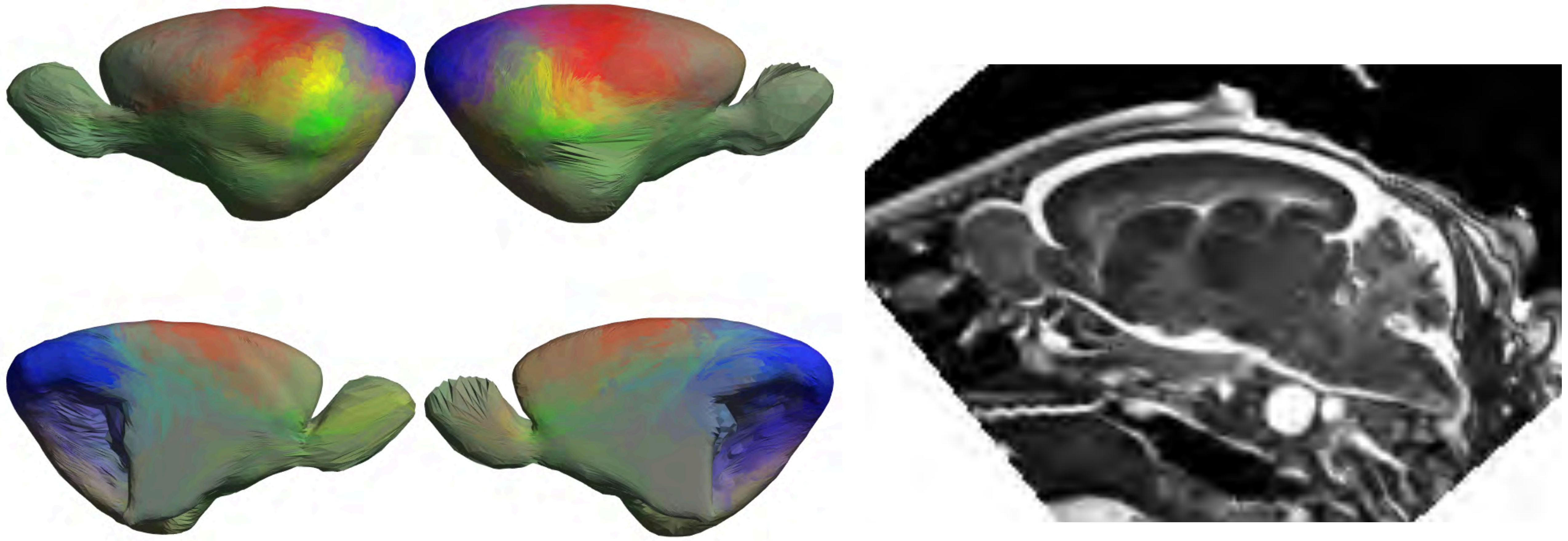

Glaucomys volans

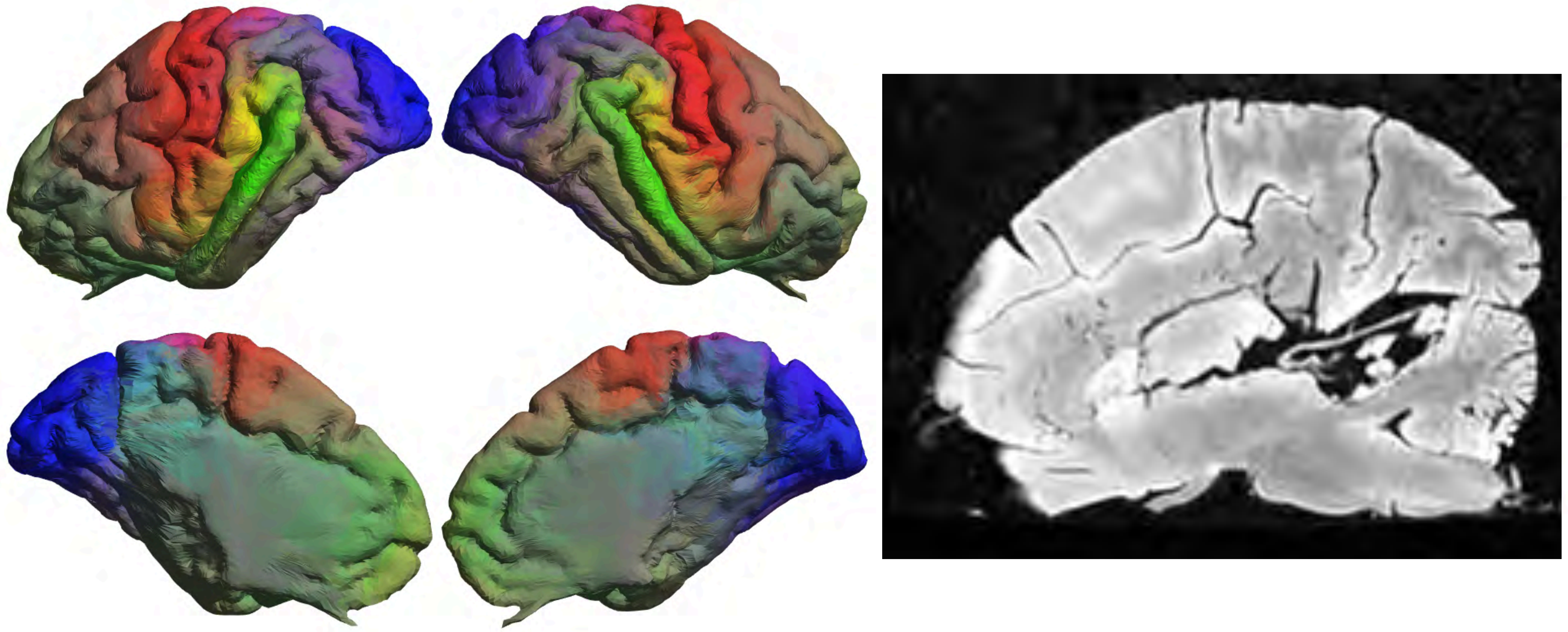

Gorilla beringei

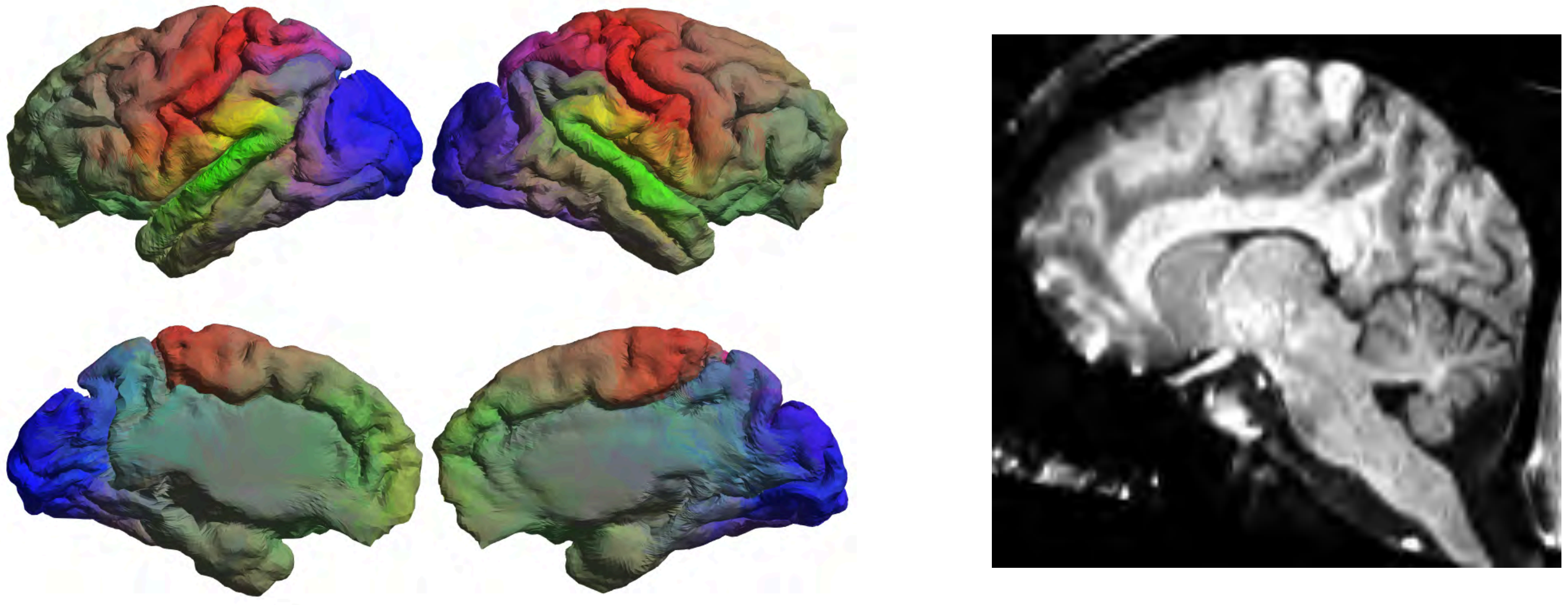

Gorilla gorilla

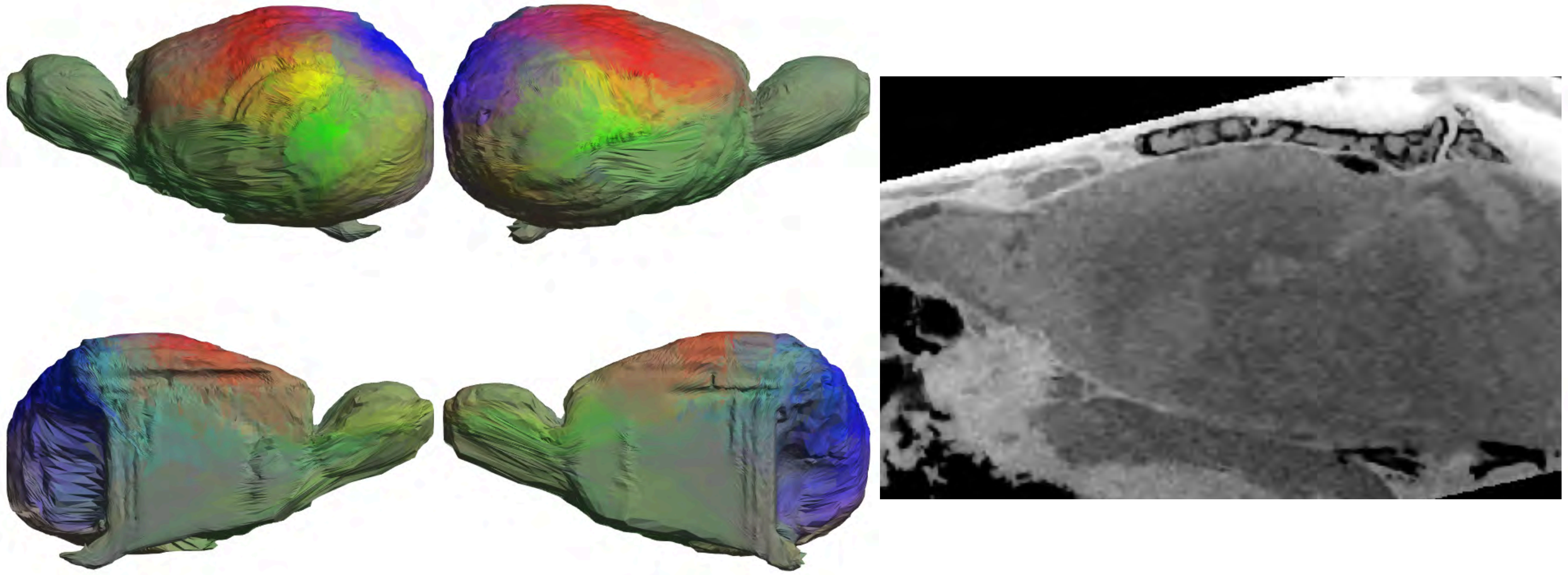

Heliophobius argenteocinereus

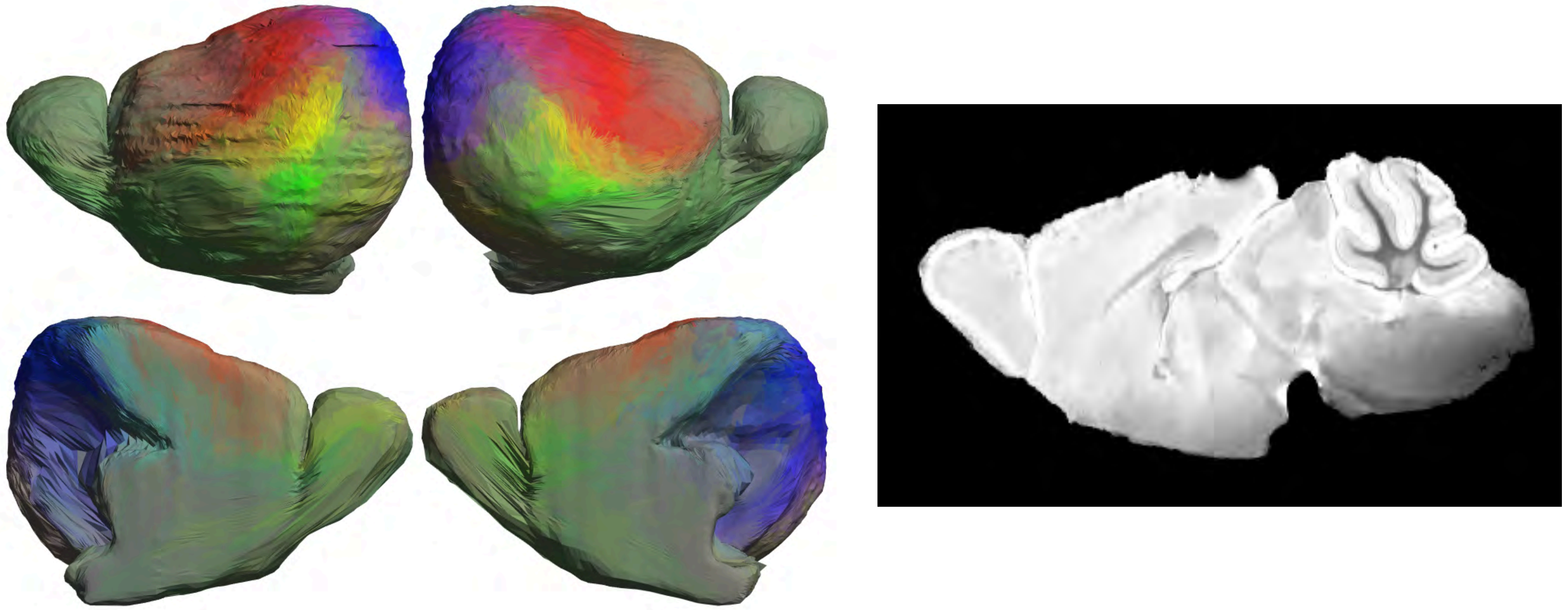

Heterocephalus glaber

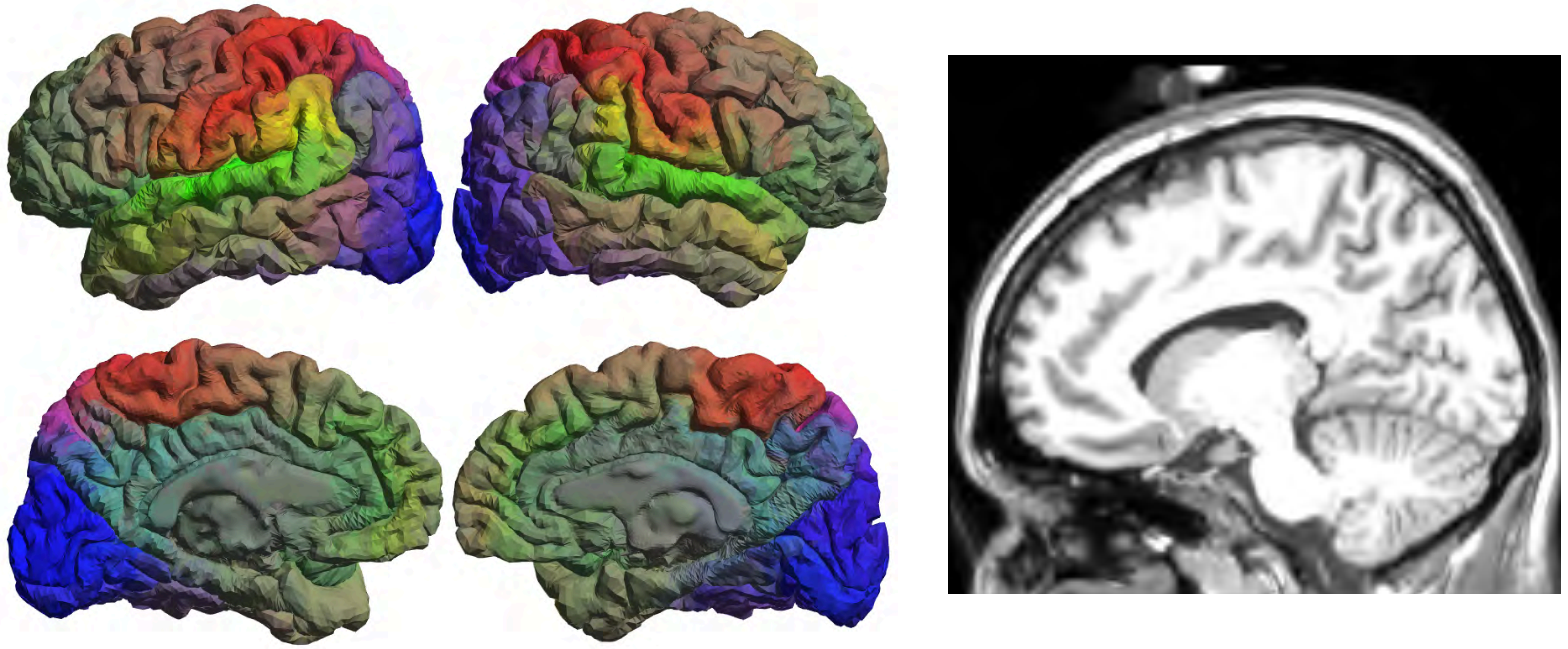

Homo sapiens

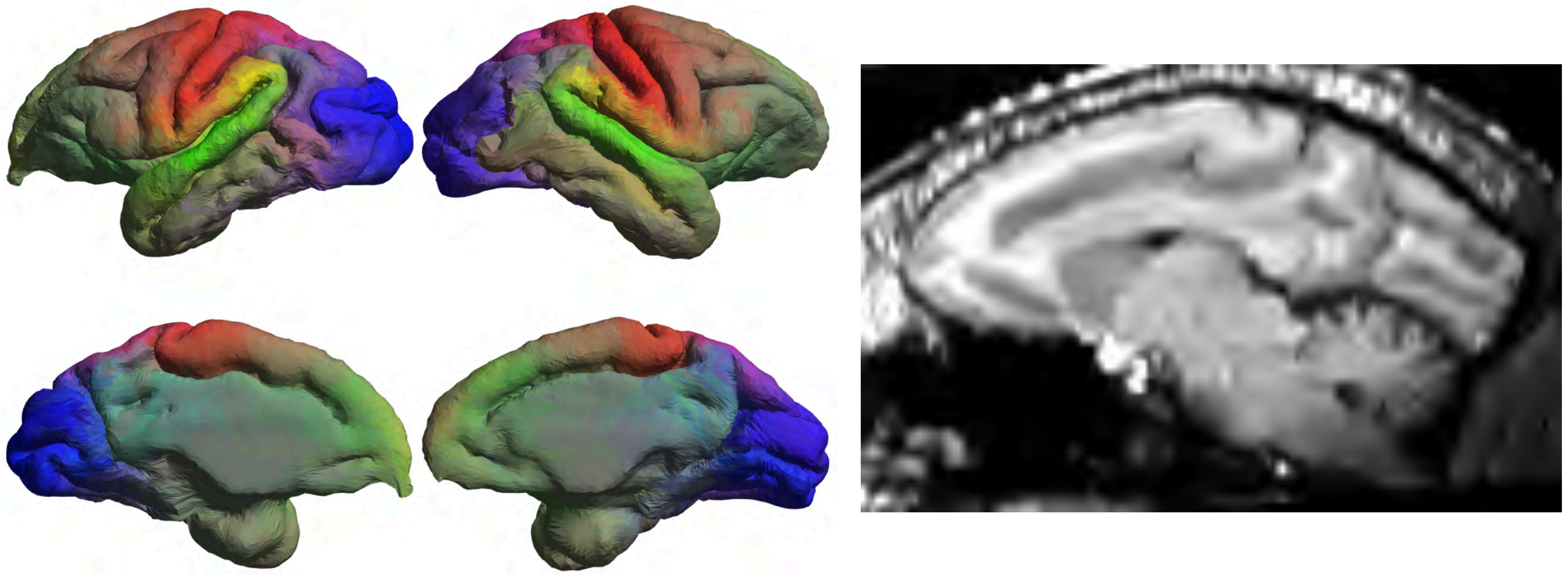

Hylobates lar

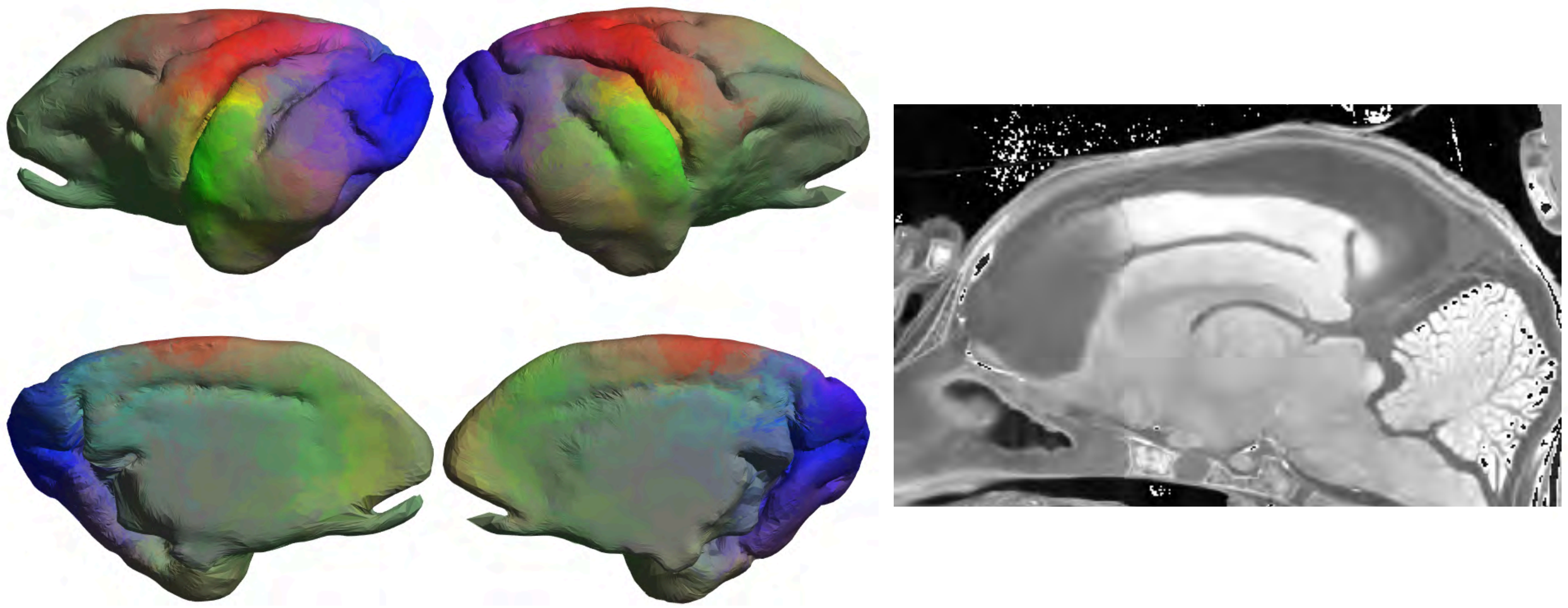

Indri indri

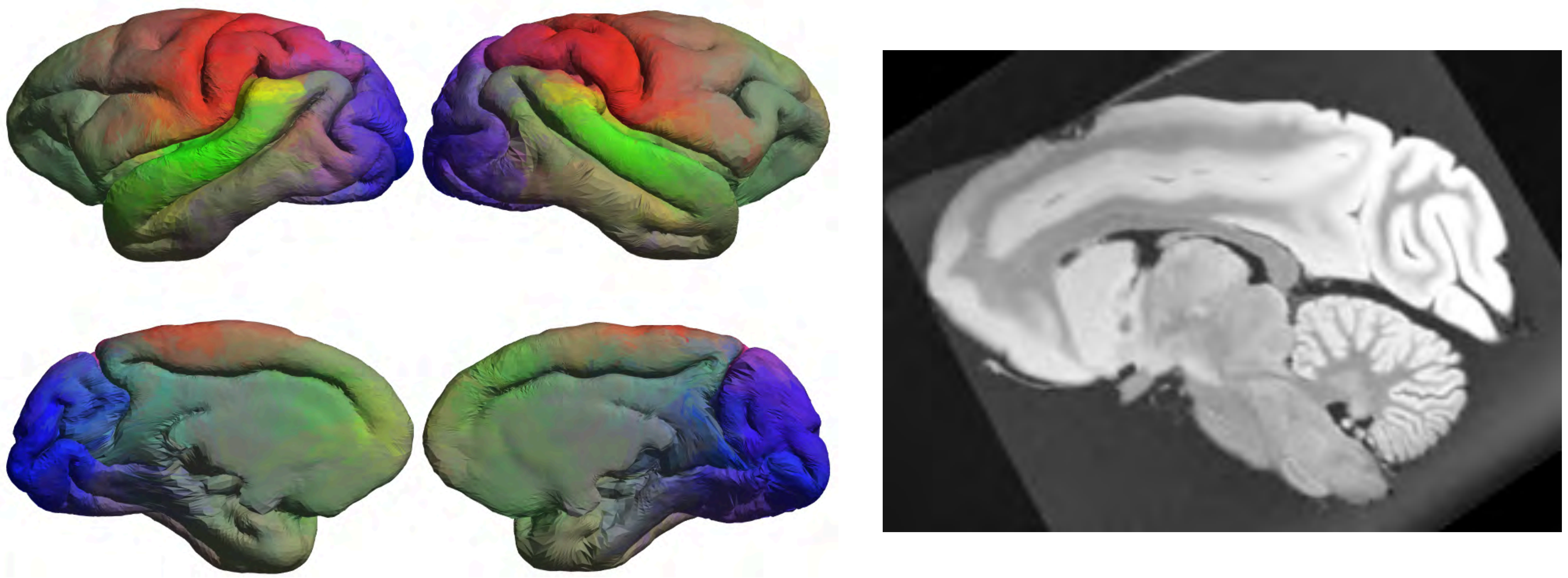

Lagothrix lagotricha

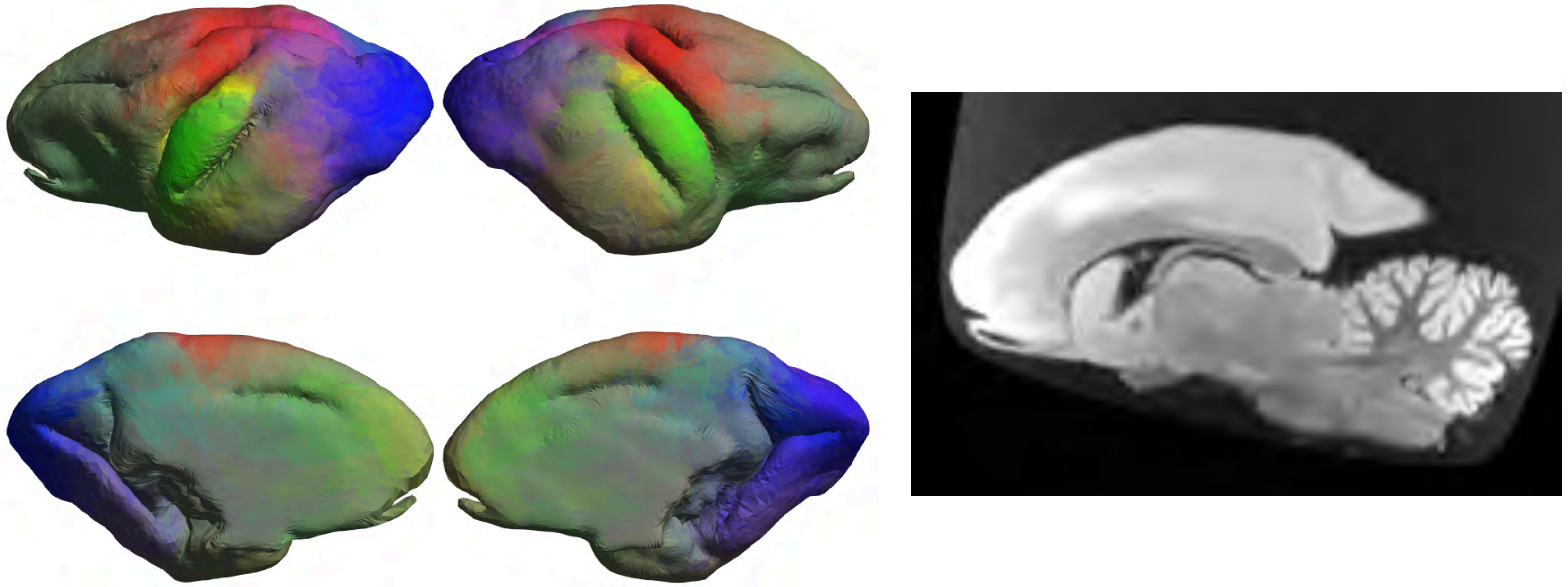

Lemur catta

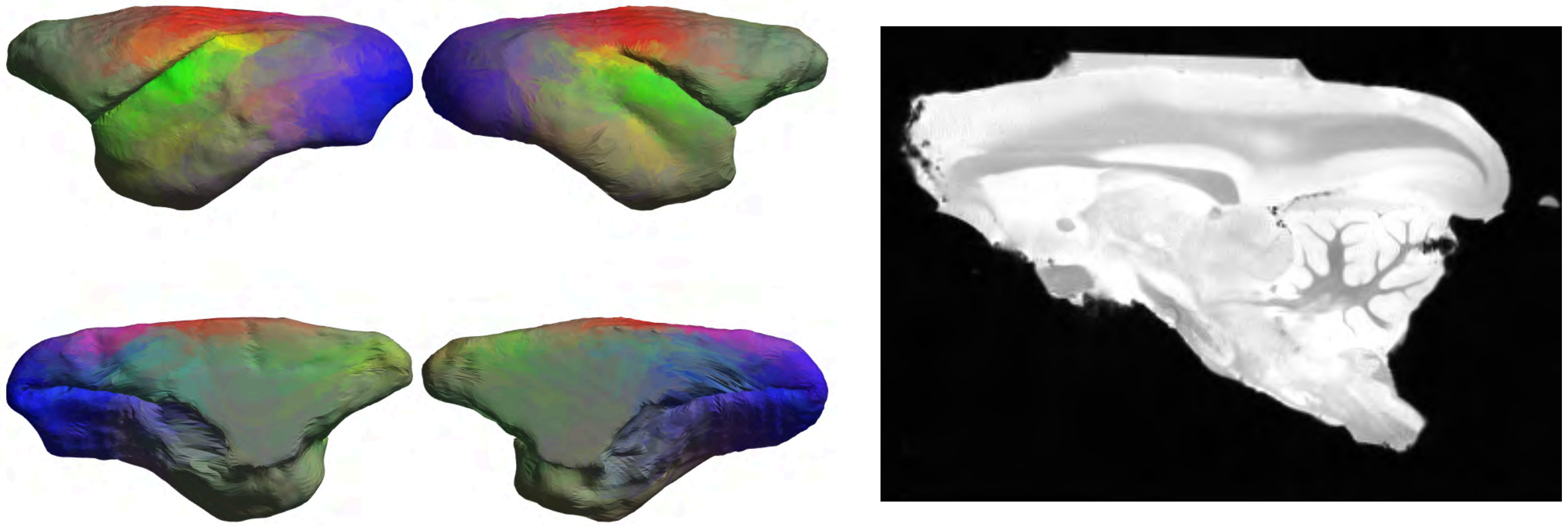

Leontocebus fuscicollis

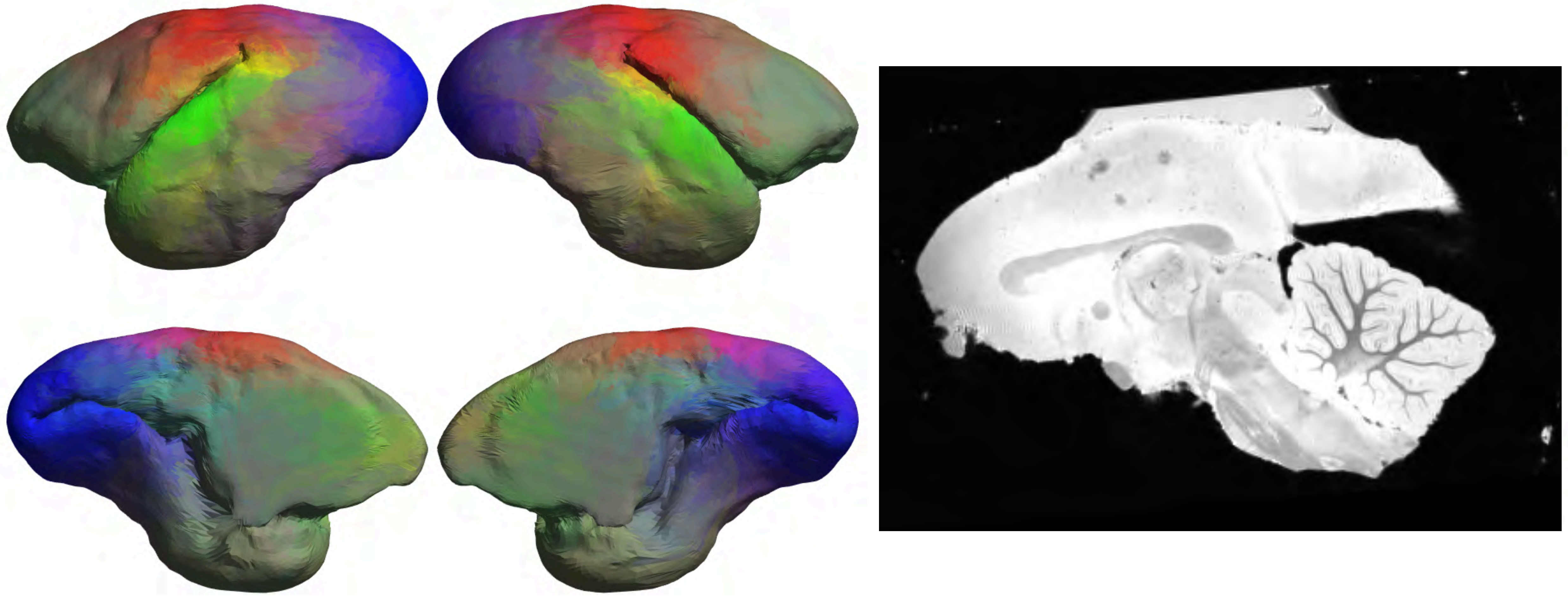

Leontopithecus rosalia

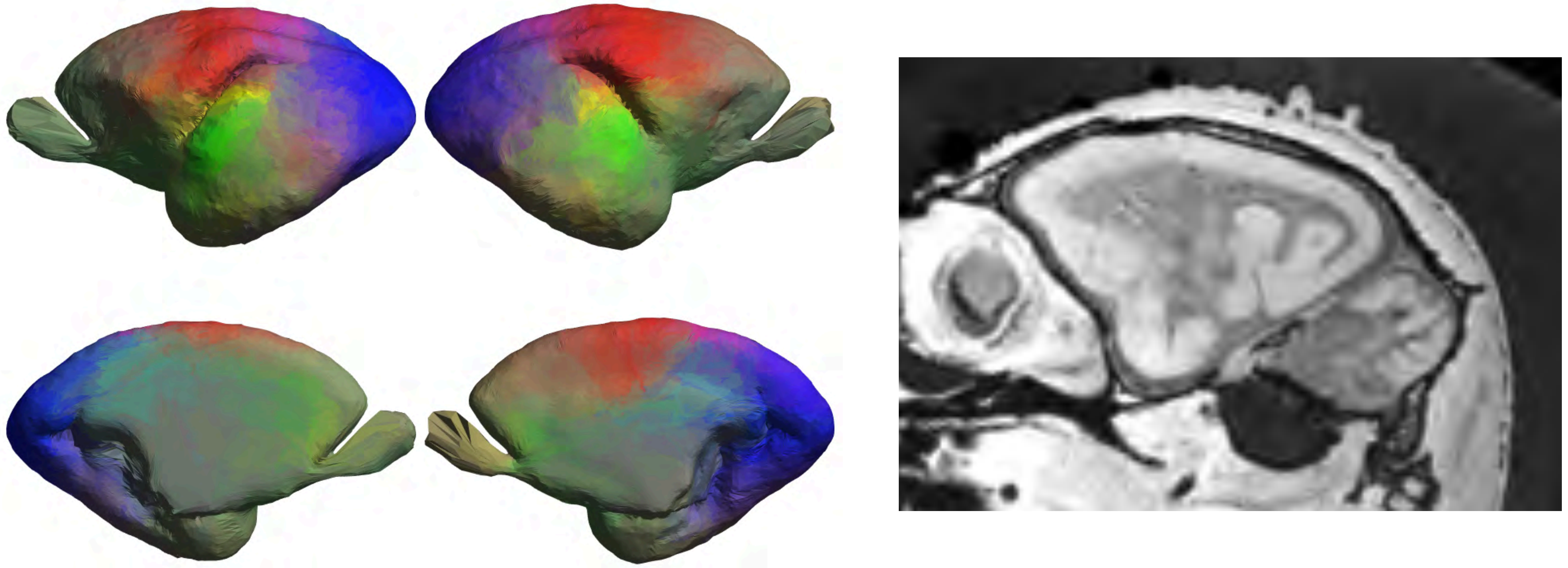

Lepilemur ruficaudatus

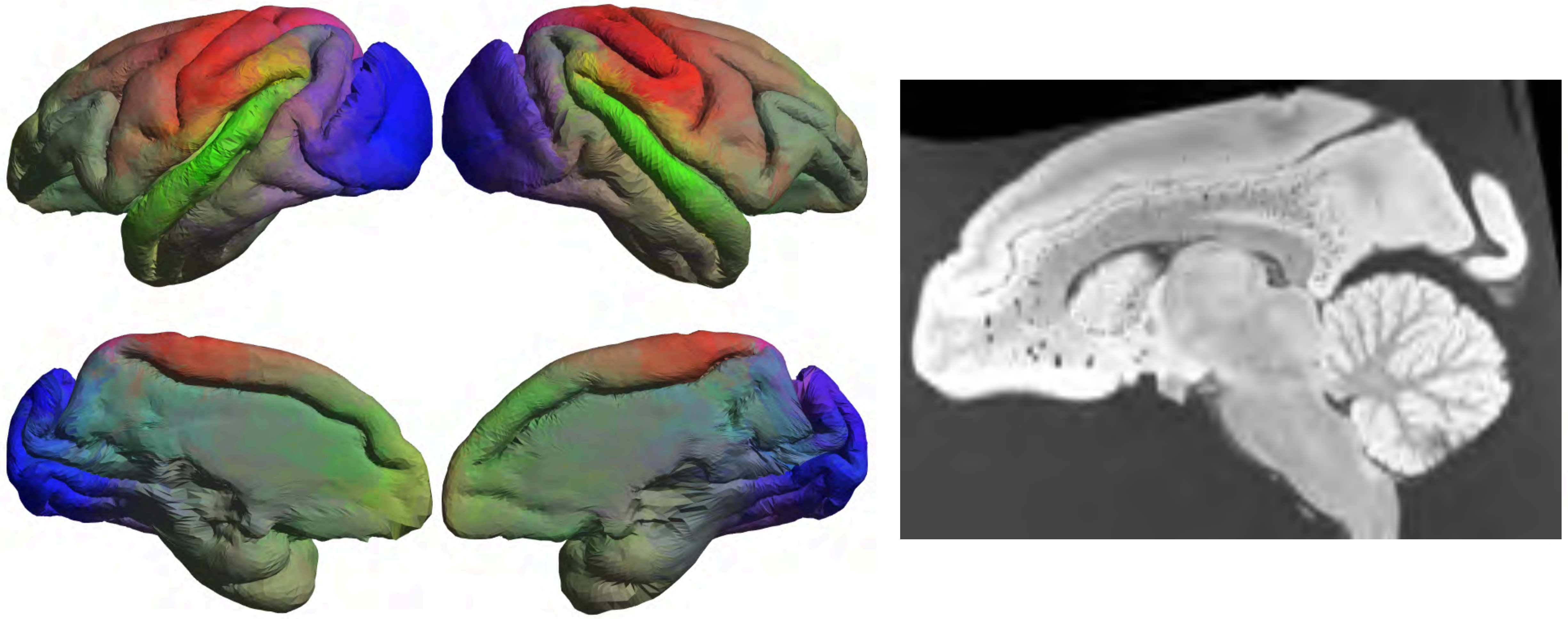

Lophocebus albigena

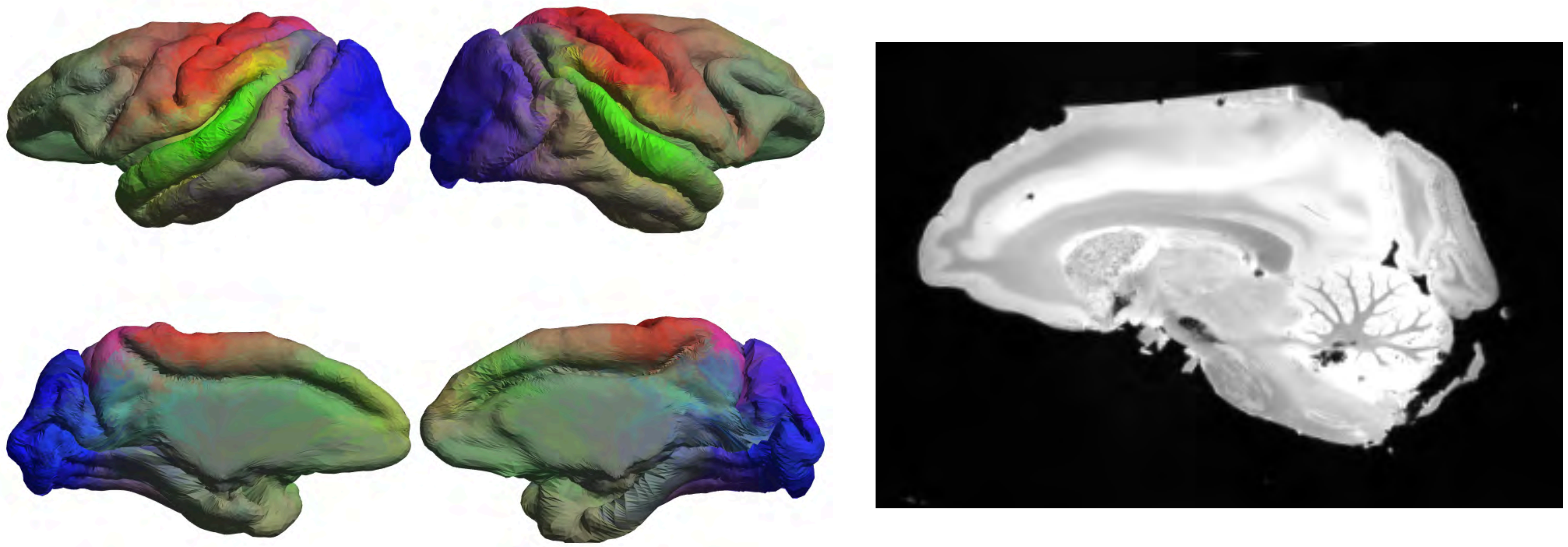

Macaca arctoides

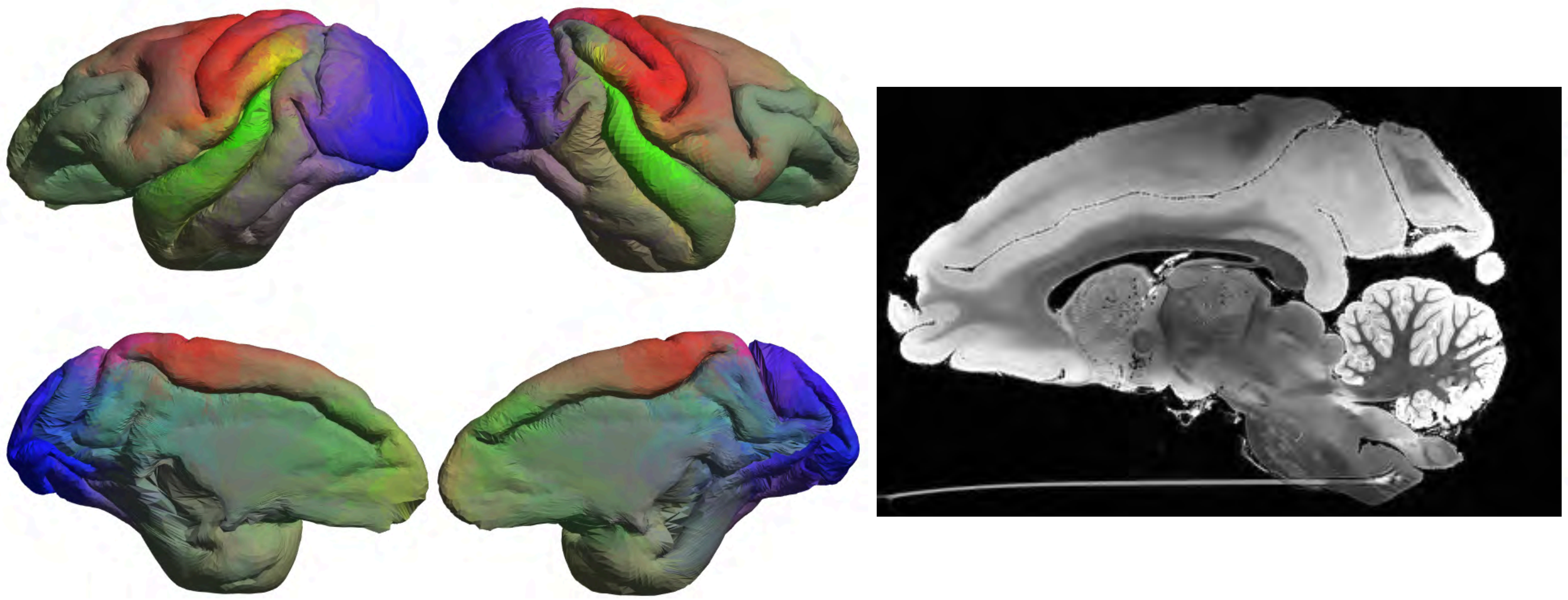

Macaca fascicularis

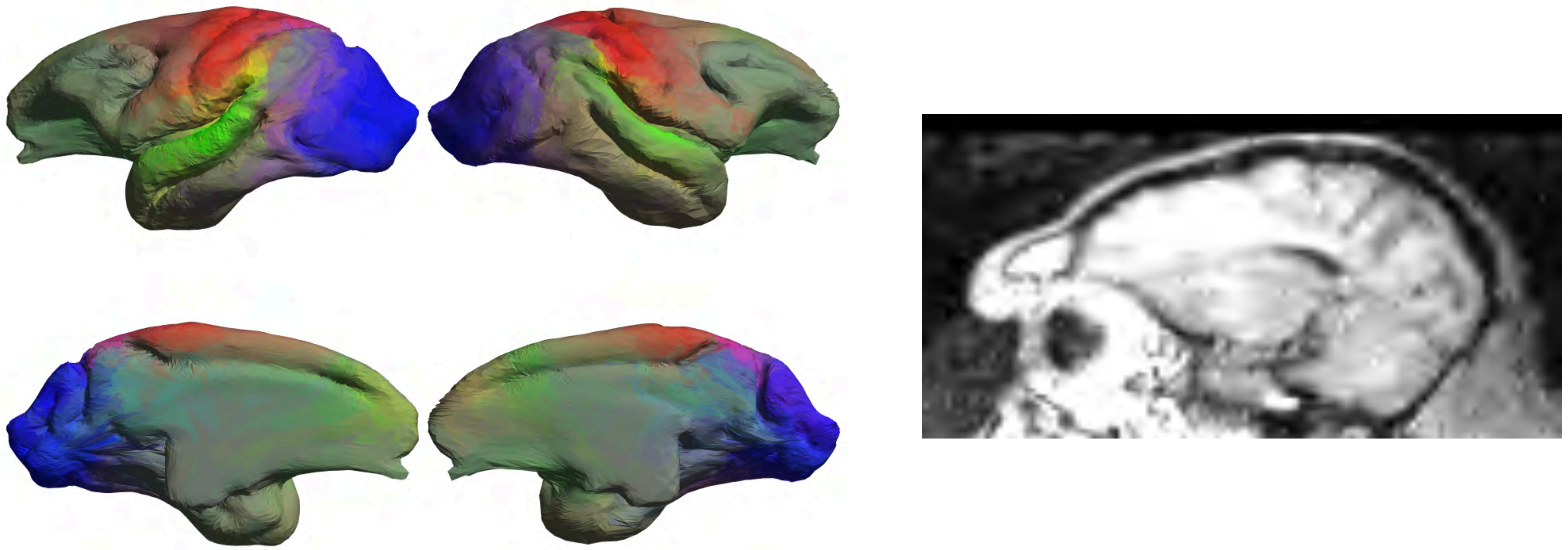

Macaca fuscata

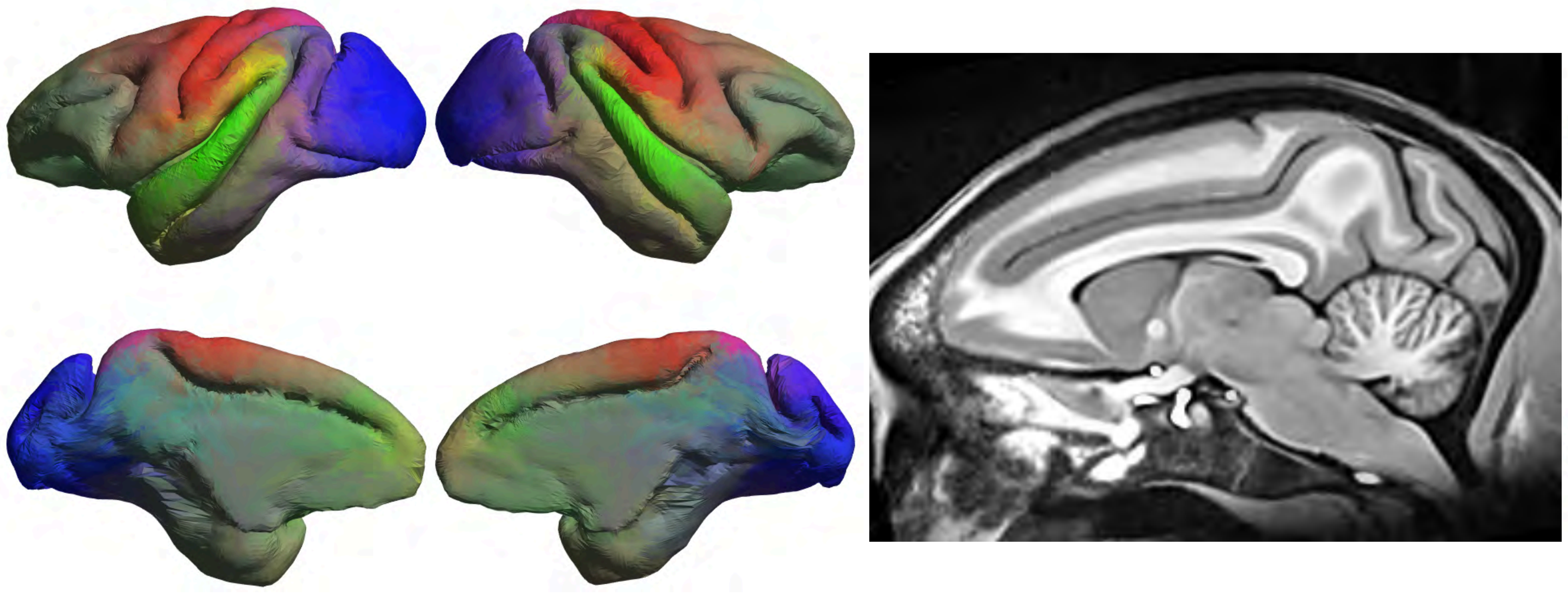

Macaca mulatta

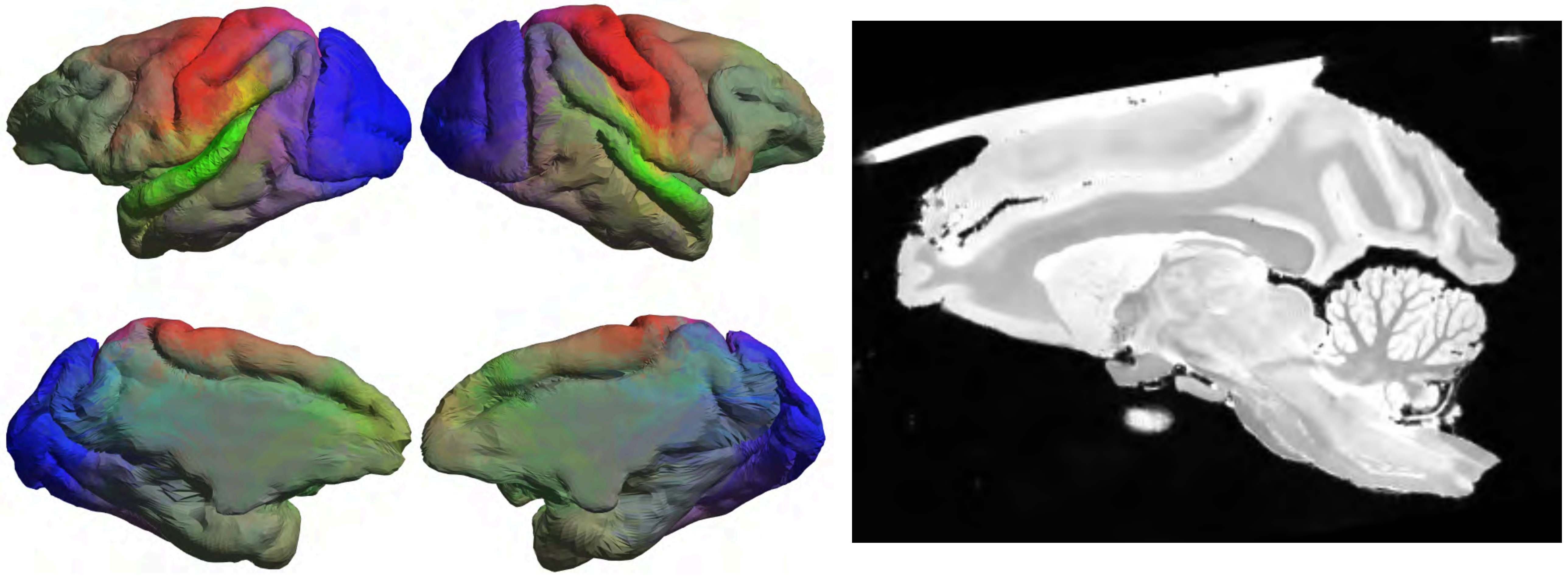

Macaca nigra

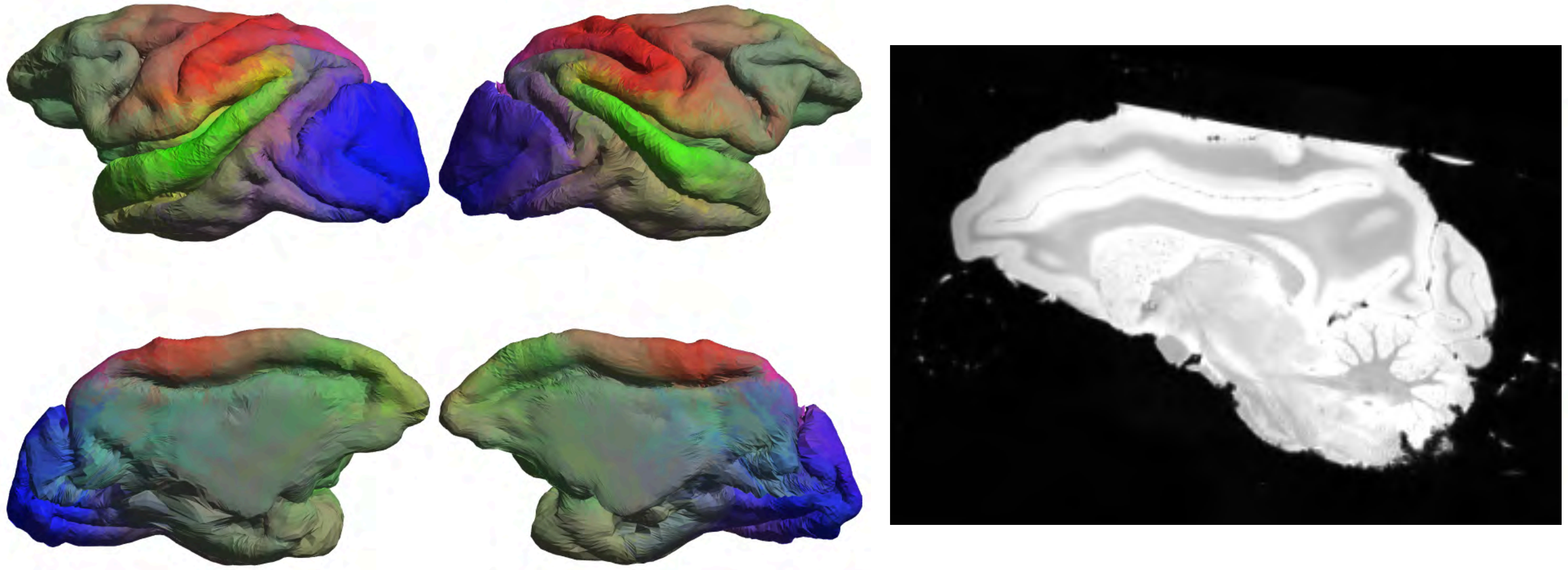

Macaca sylvanus

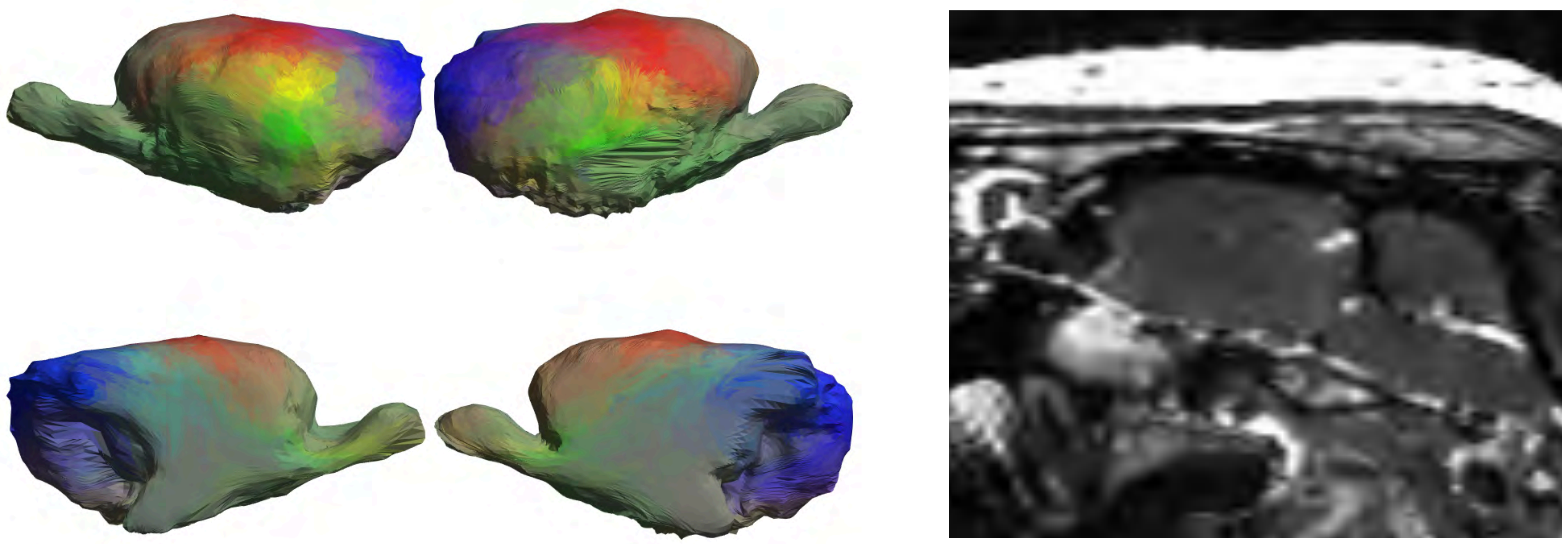

Marmota marmota

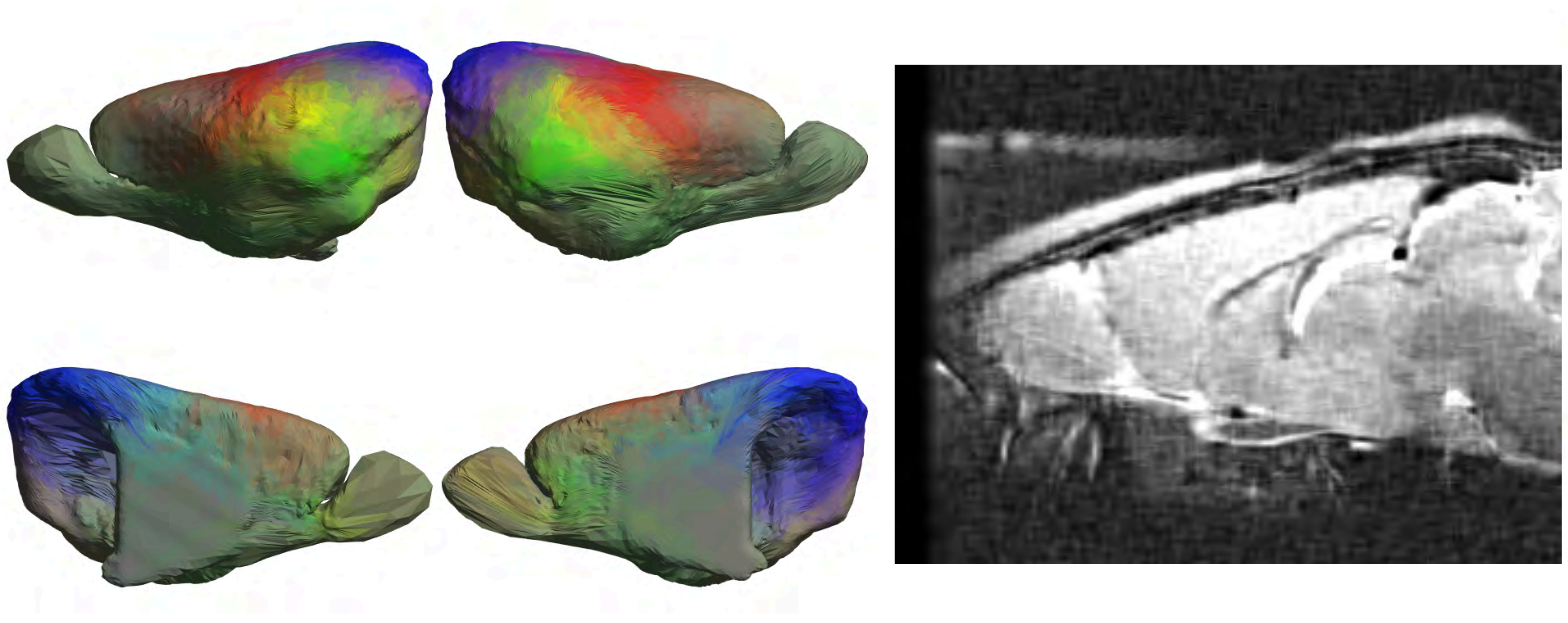

Meriones unguiculatus

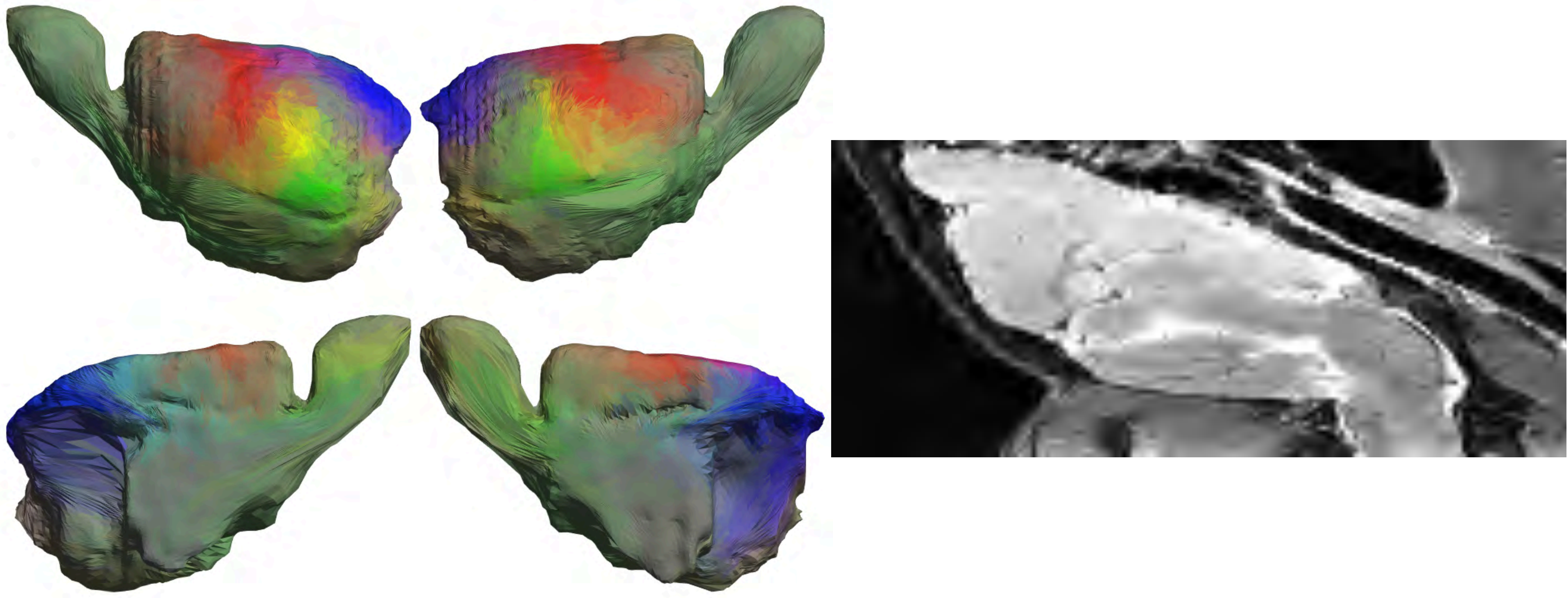

Mesocricetus auratus

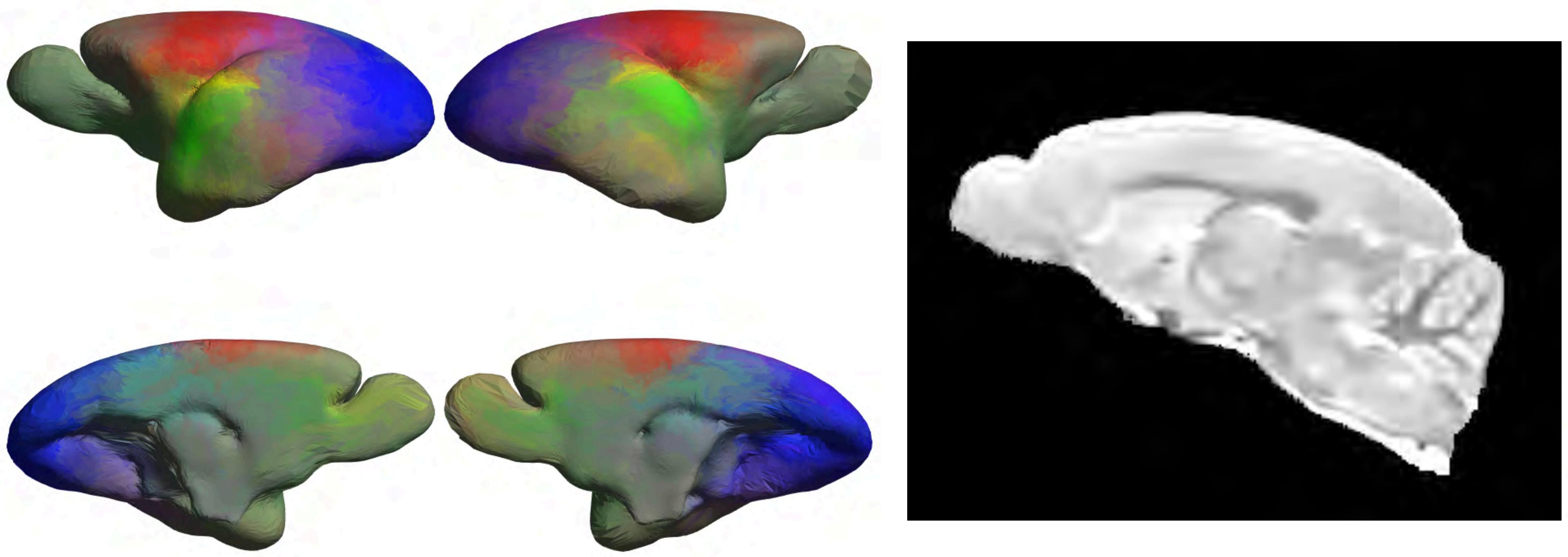

Microcebus murinus

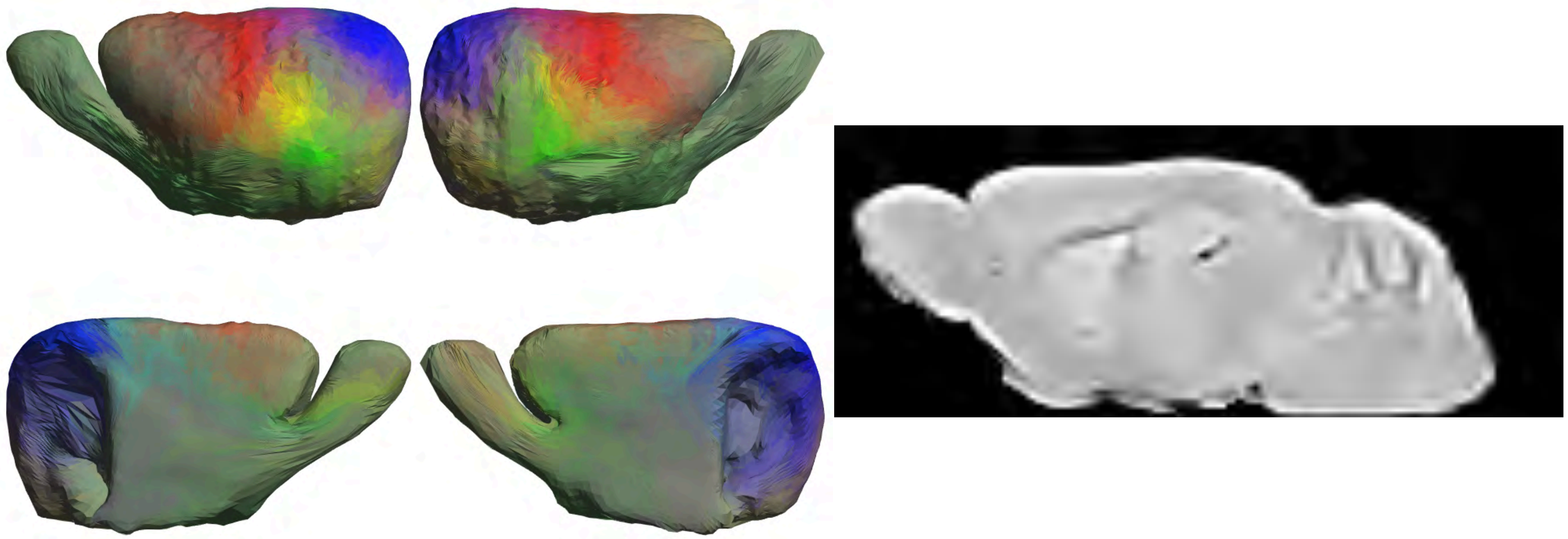

Microtus ochrogaster

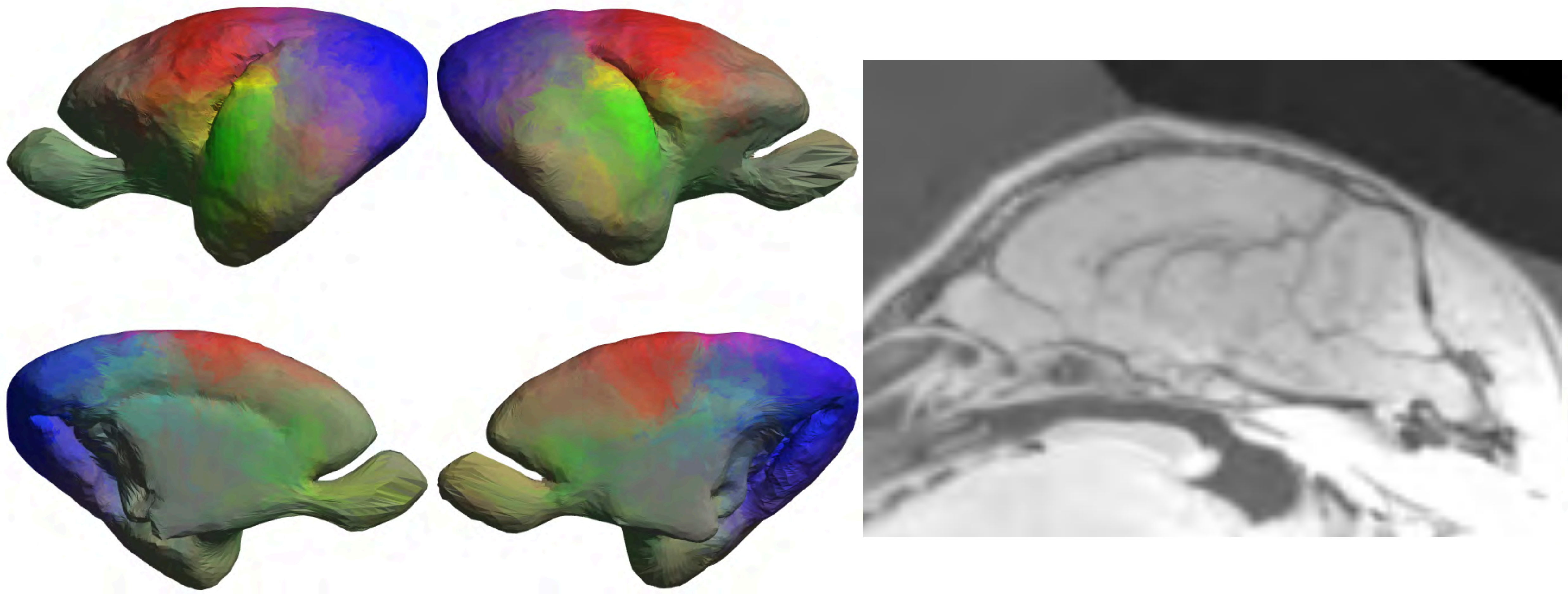

Mirza coquereli

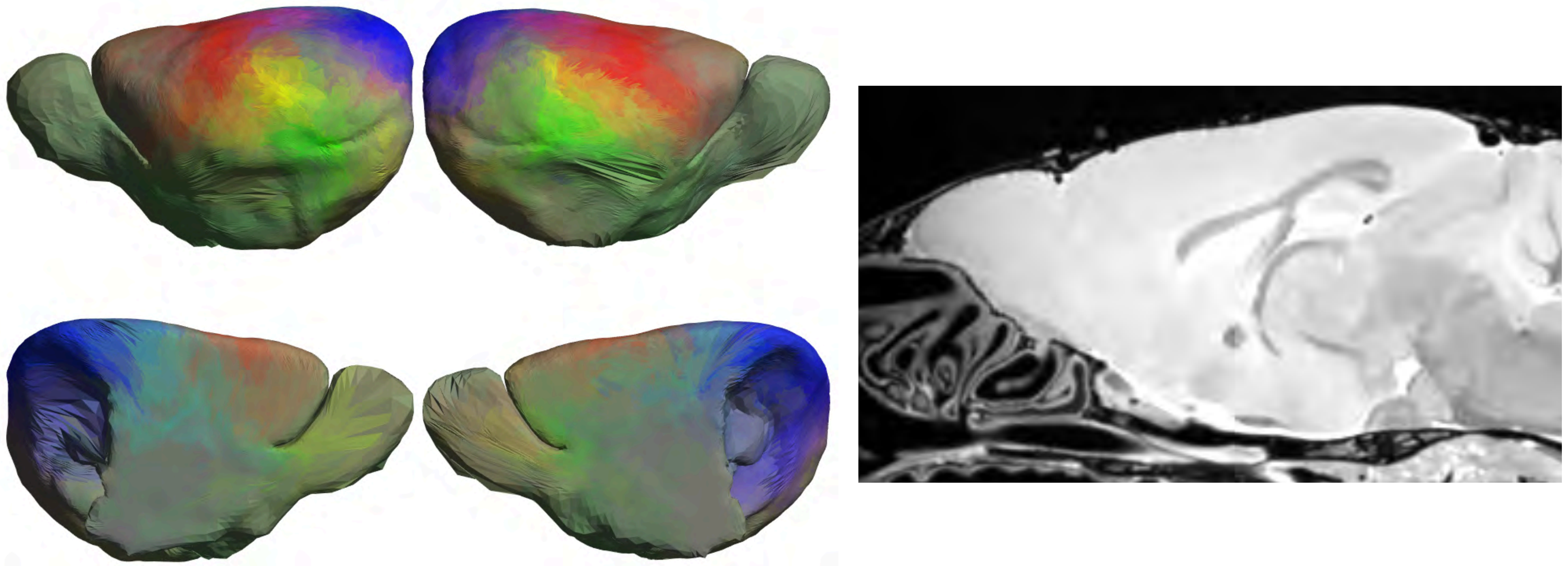

Mus musculus

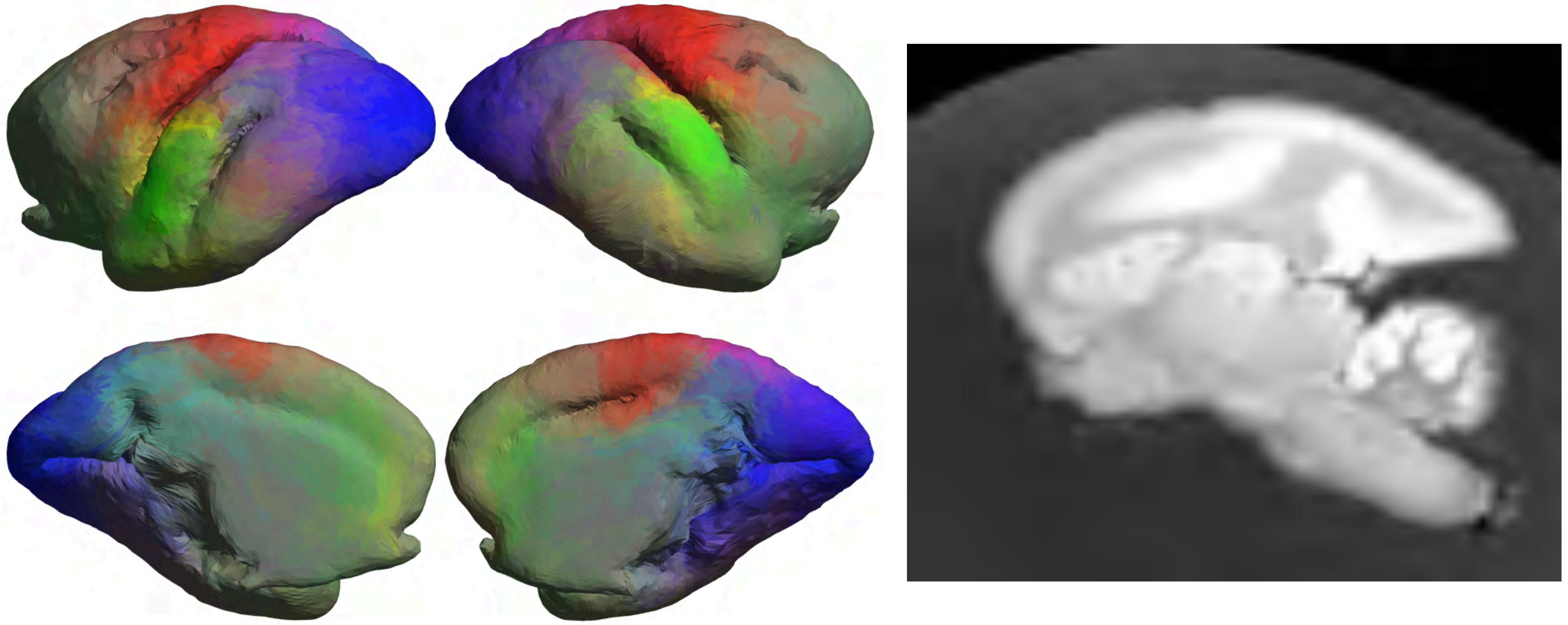

Nycticebus bengalensis

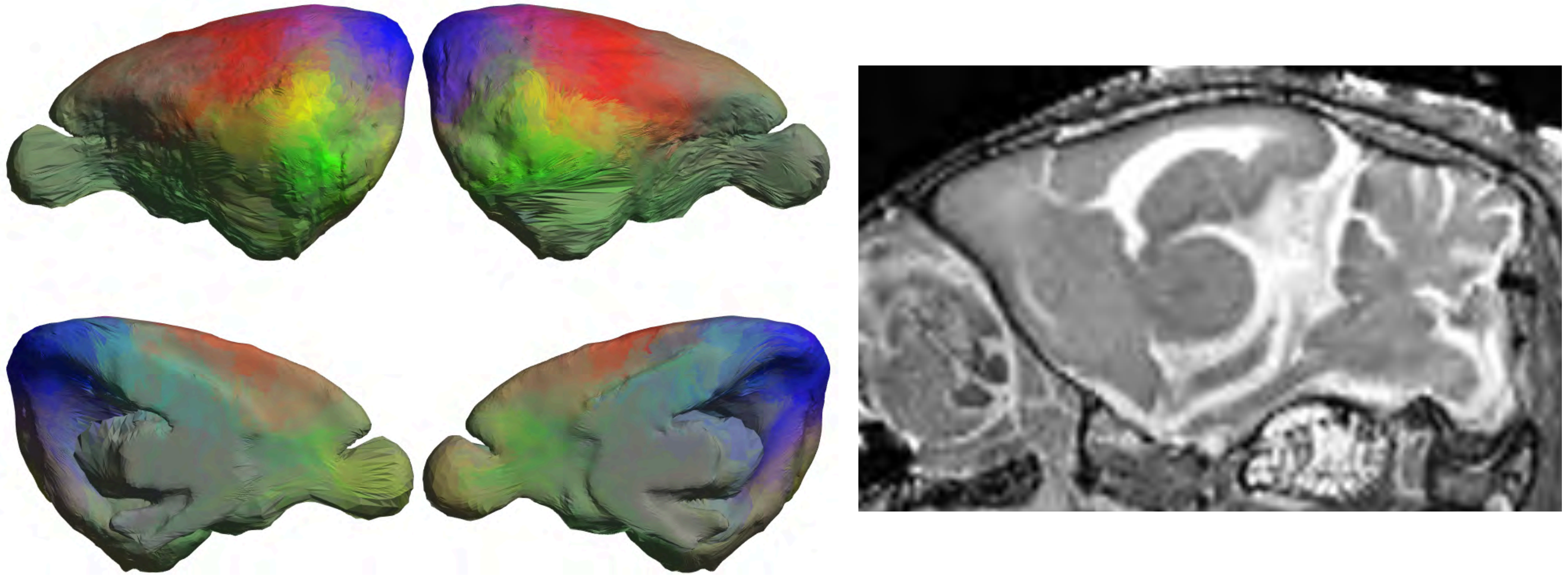

Ochotona macrotis

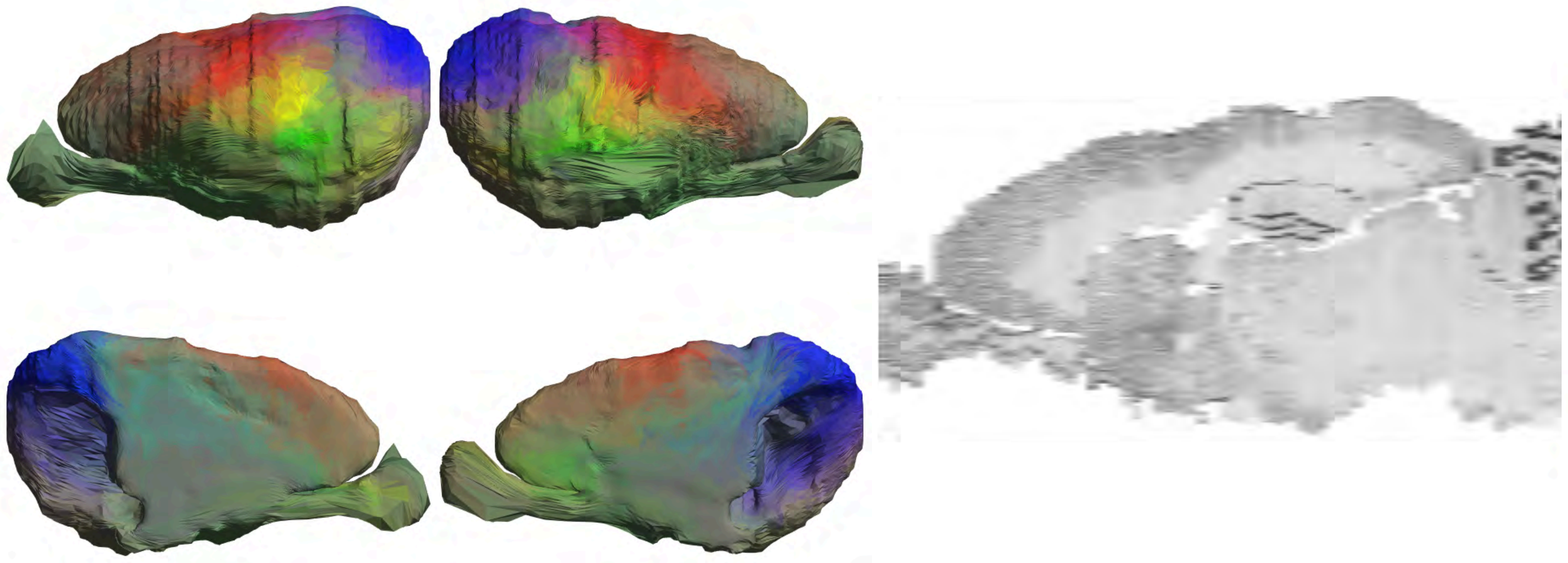

Octodon degus

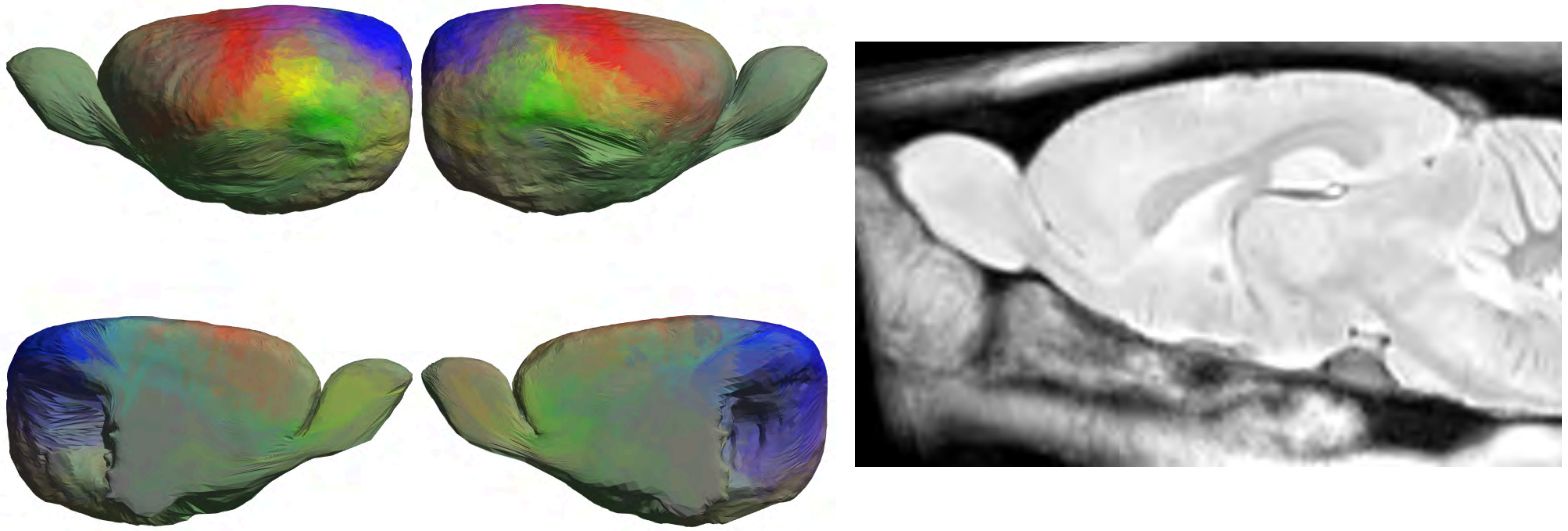

Ondatra zibethicus

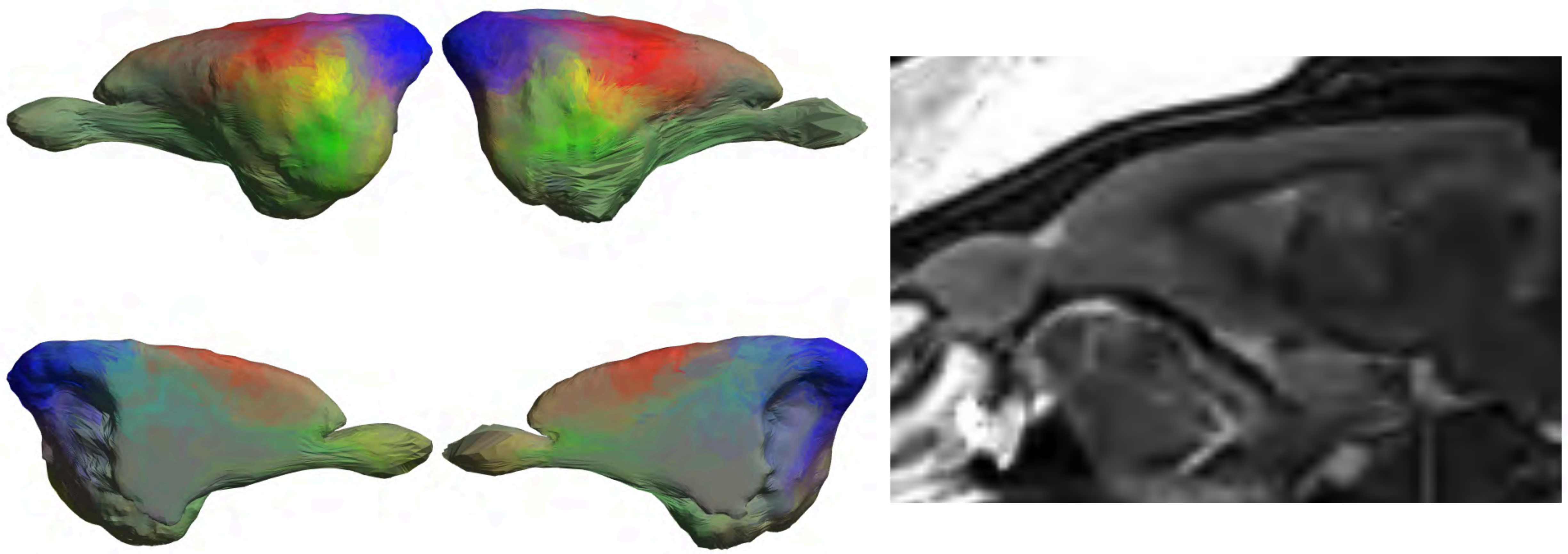

Oryctolagus cuniculus

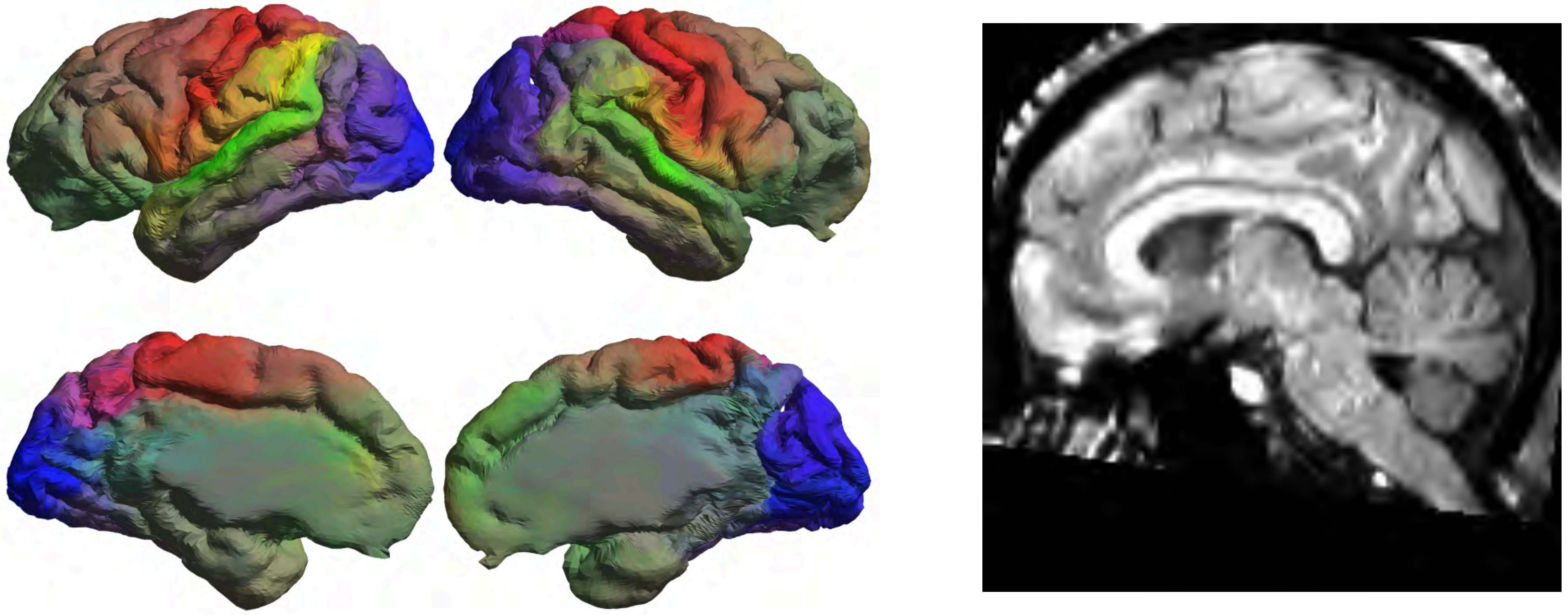

Pan paniscus

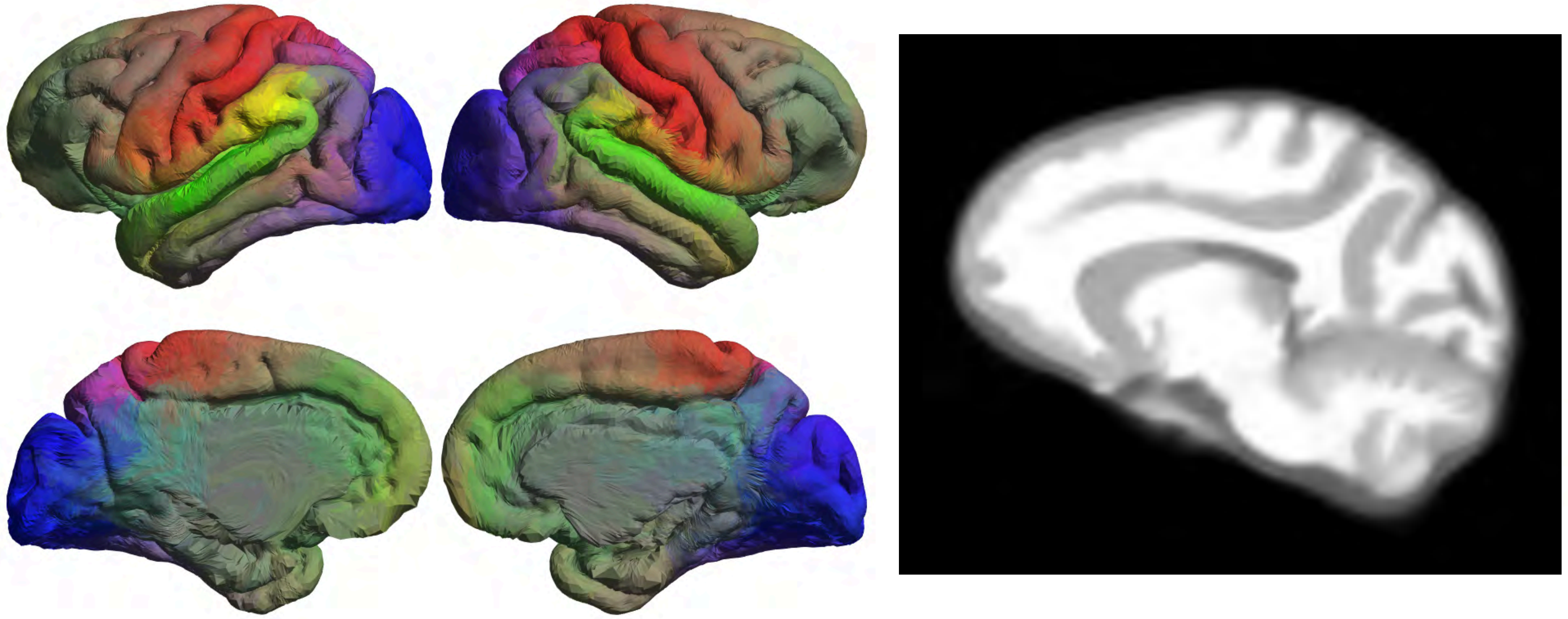

Pan troglodytes

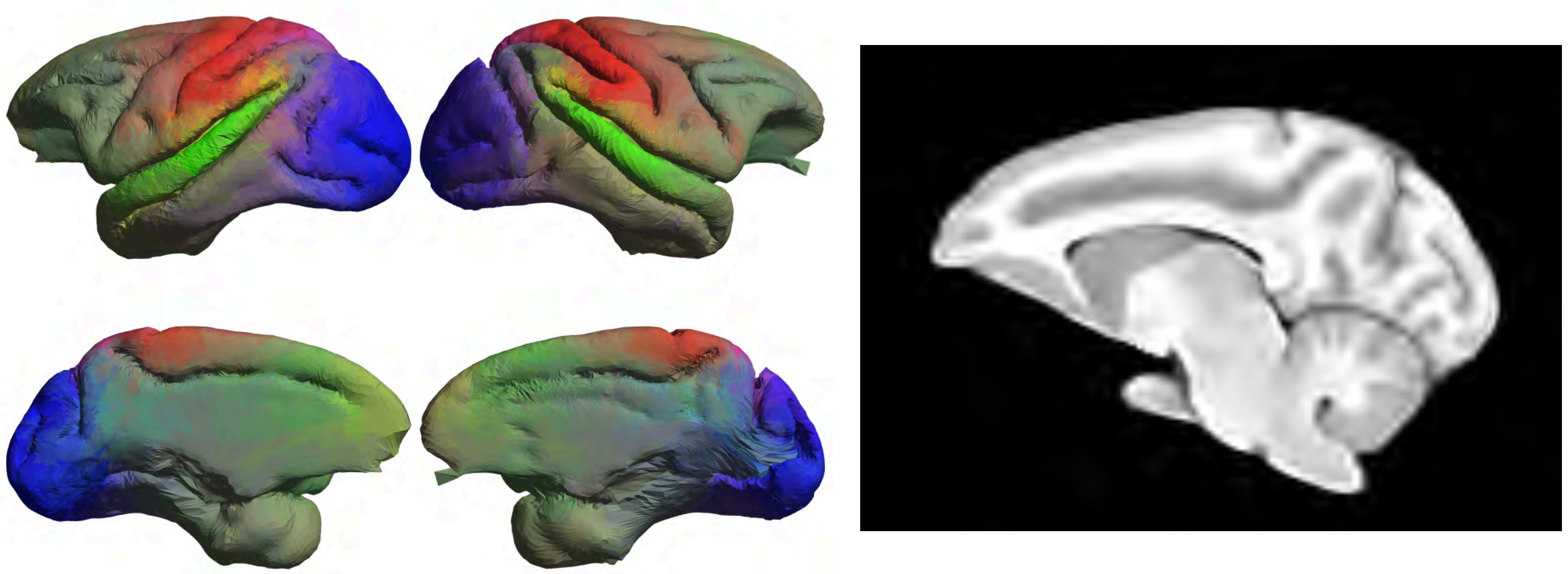

Papio anubis

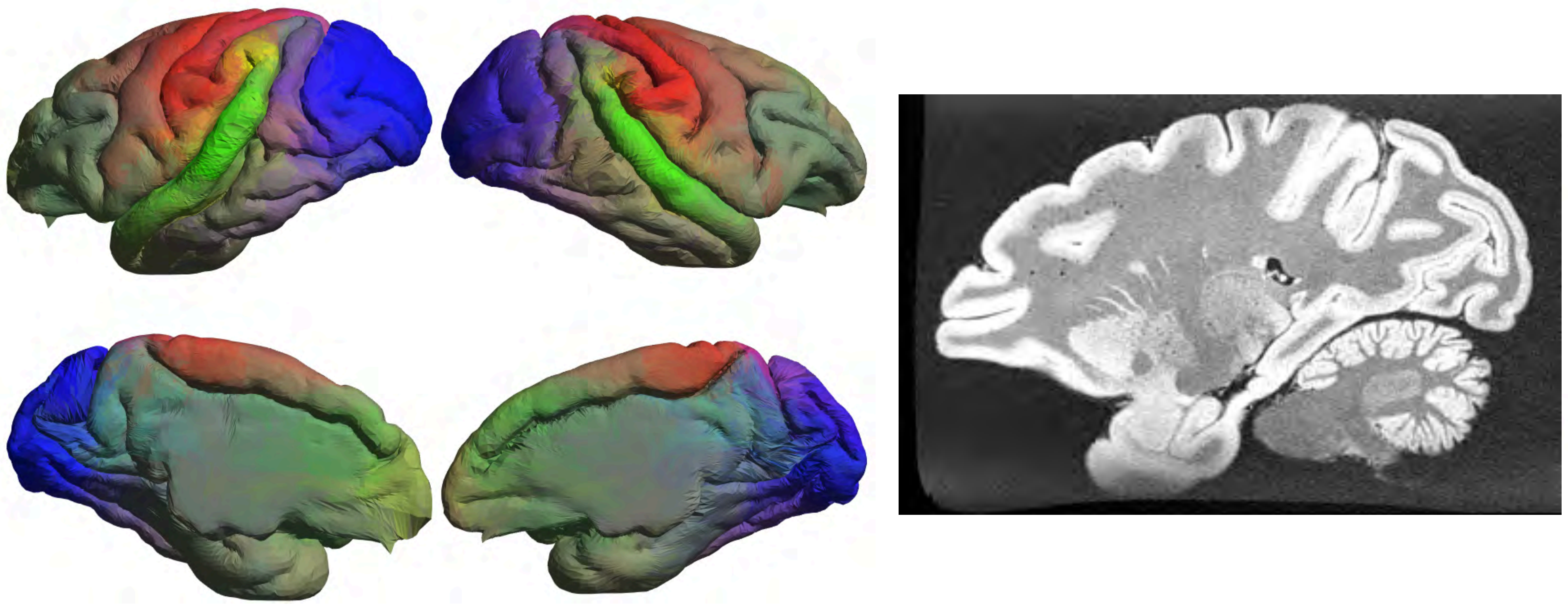

Papio hamadryas

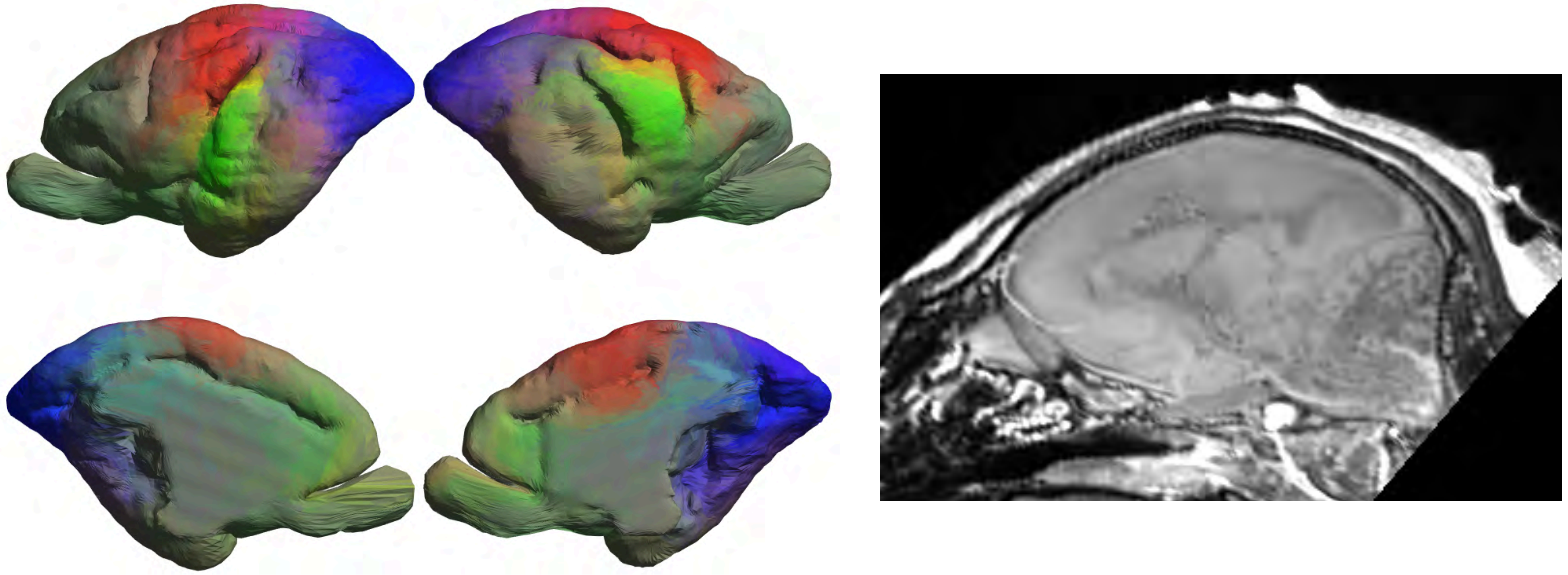

Perodicticus potto

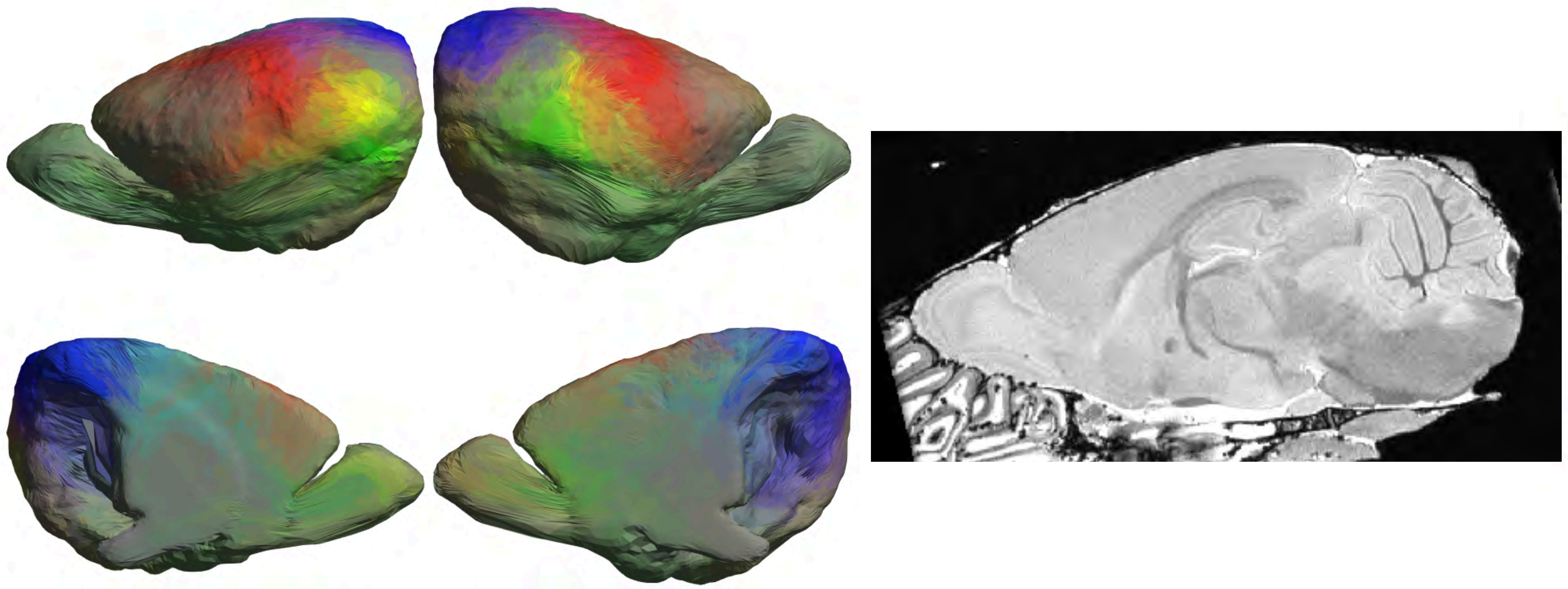

Peromyscus californicus

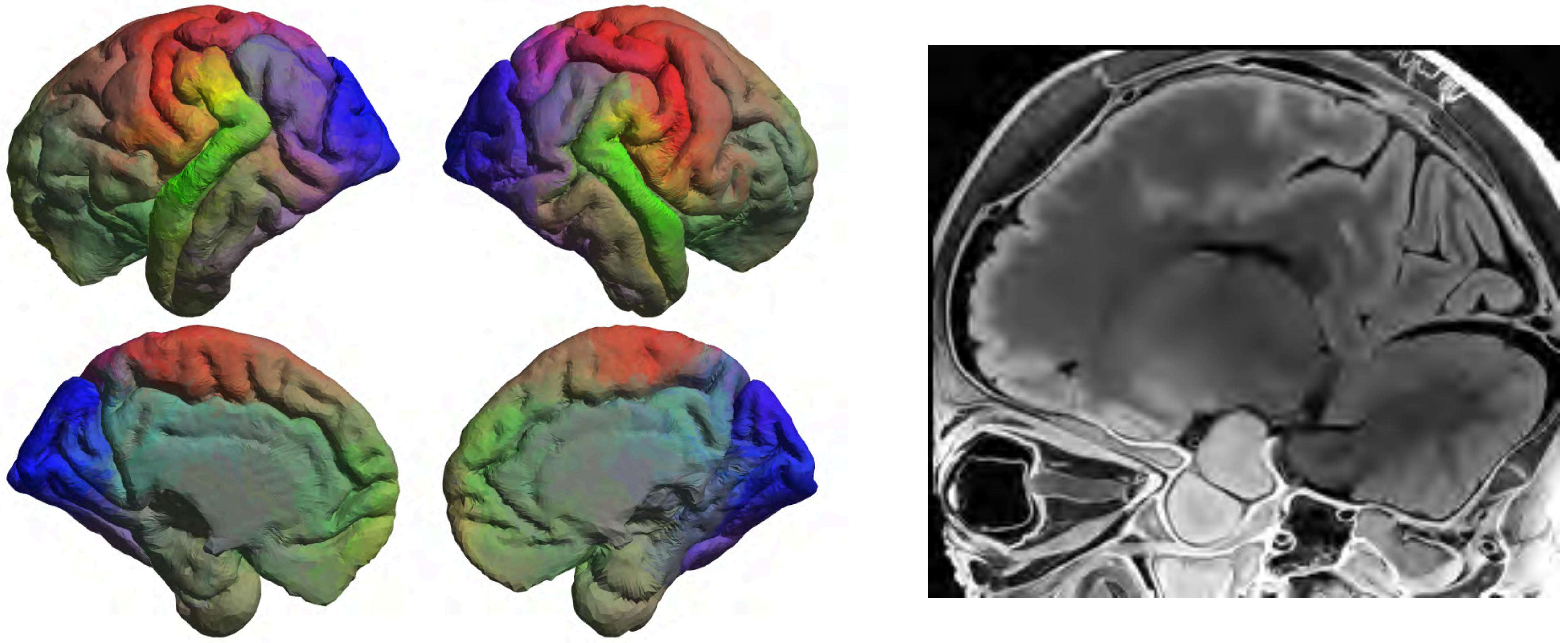

Pongo pygmaeus

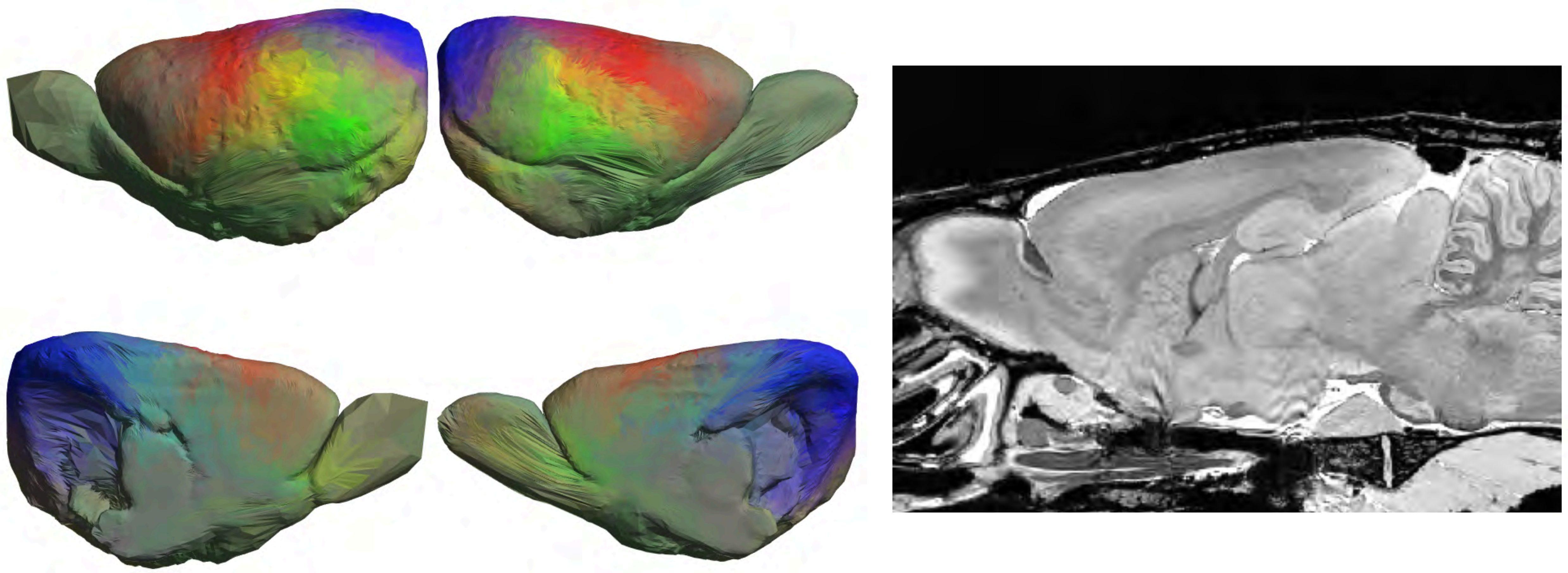

Rattus norvegicus

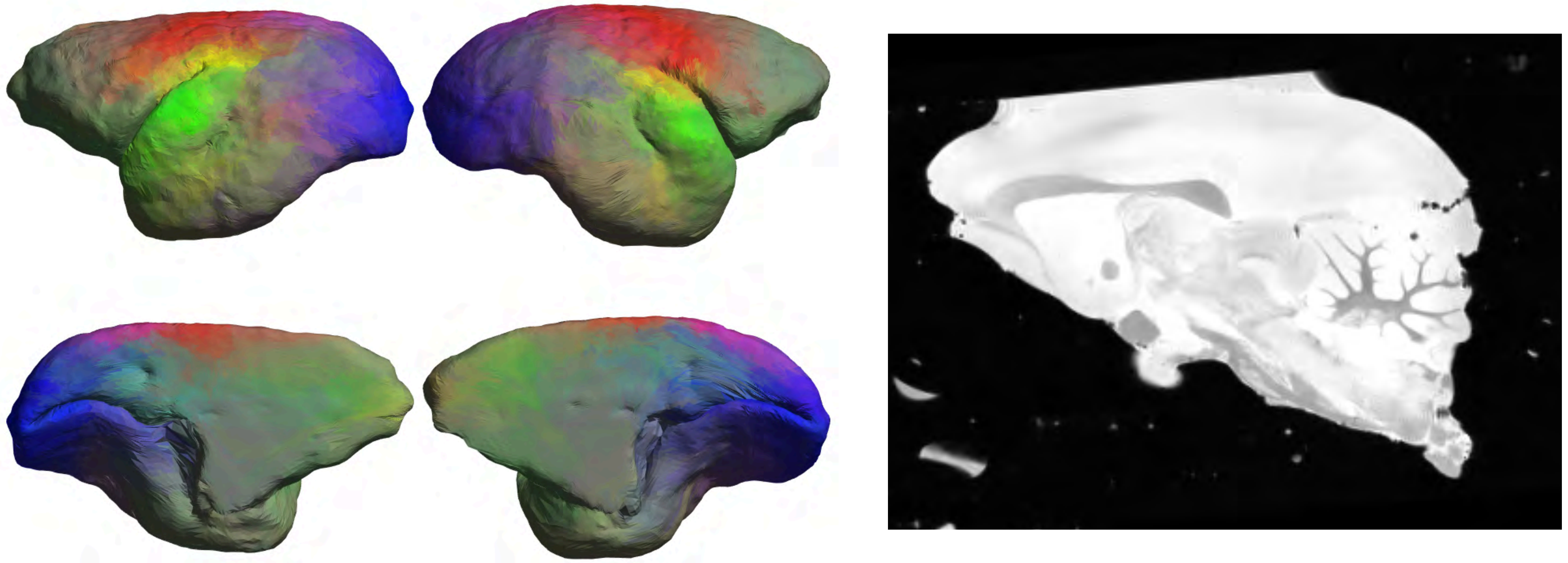

Saguinus imperator

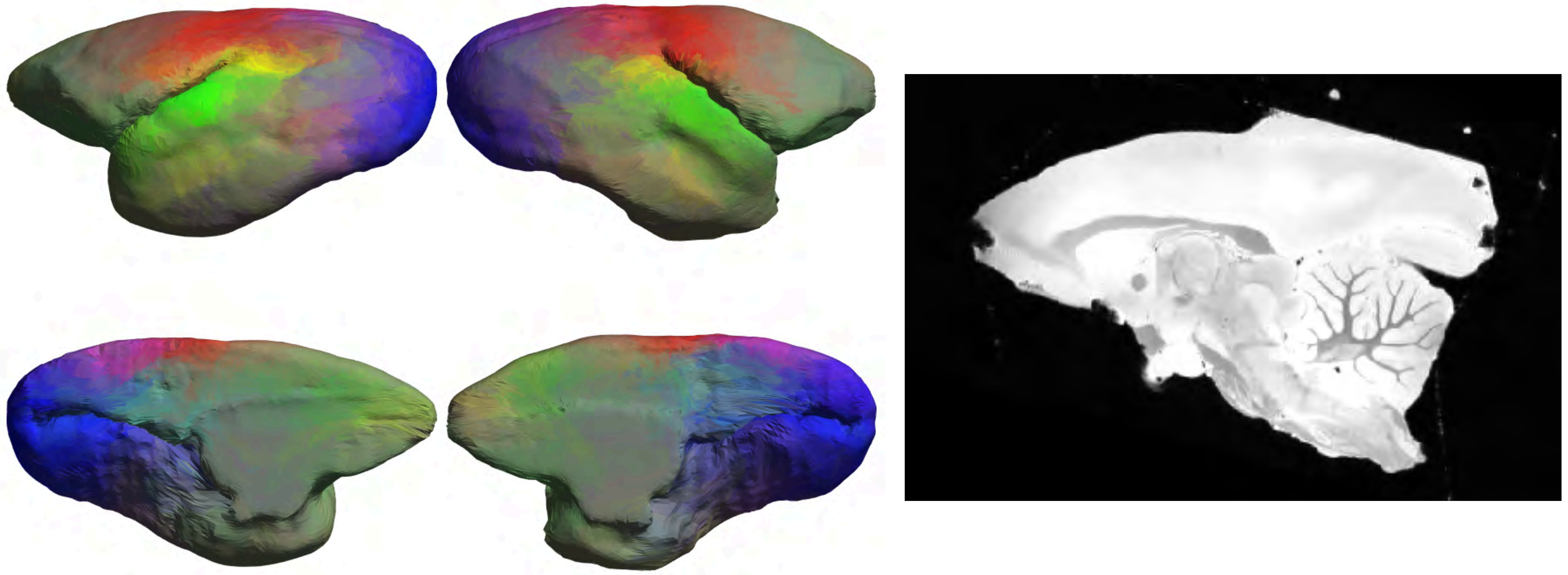

Saguinus midas

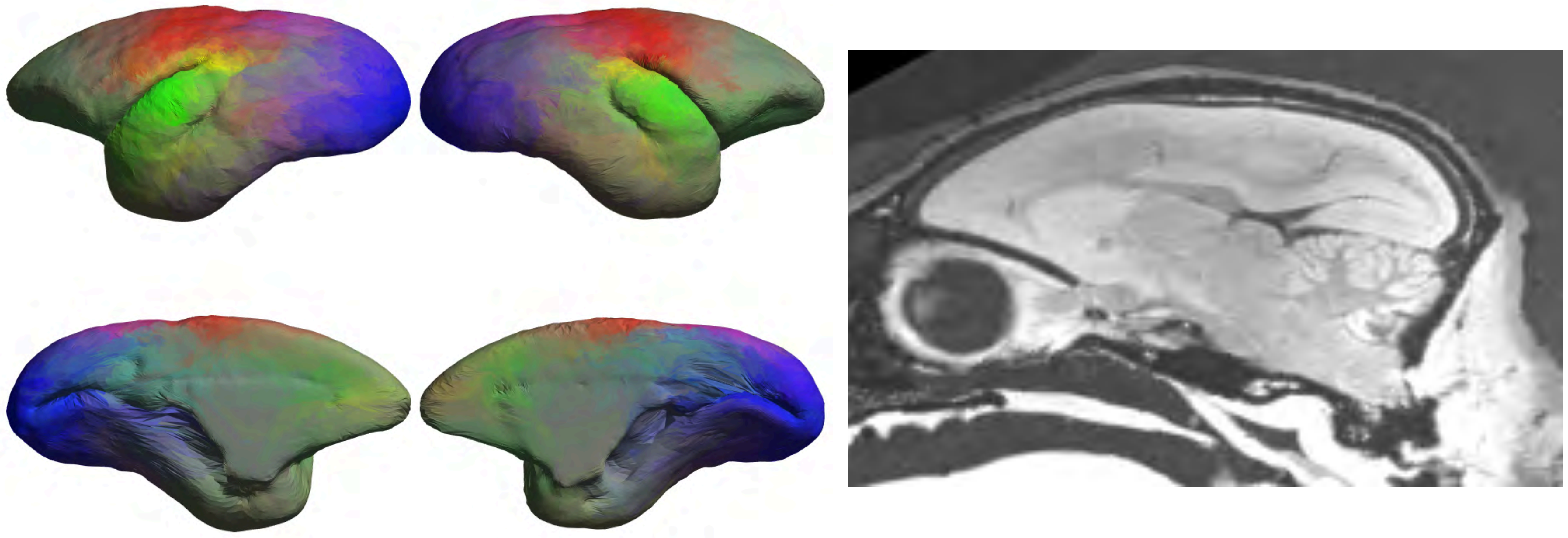

Saguinus oedipus

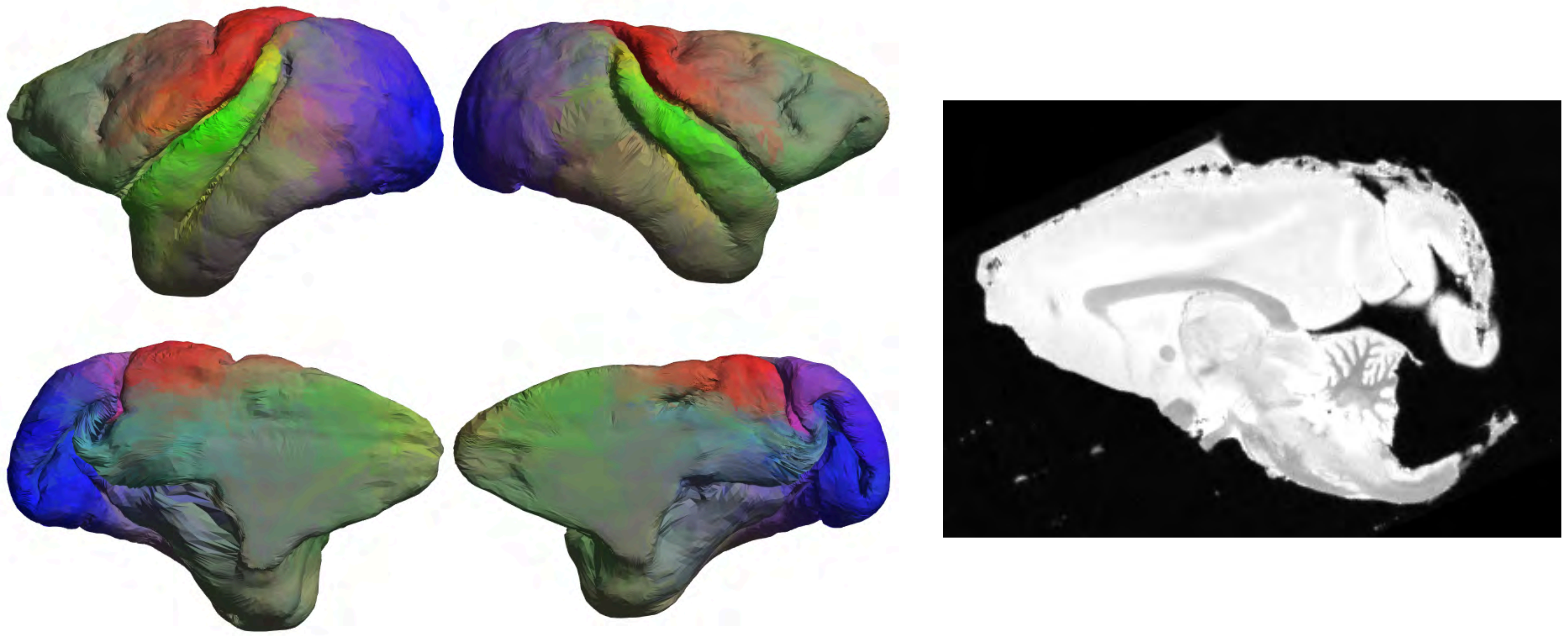

Saimiri boliviensis

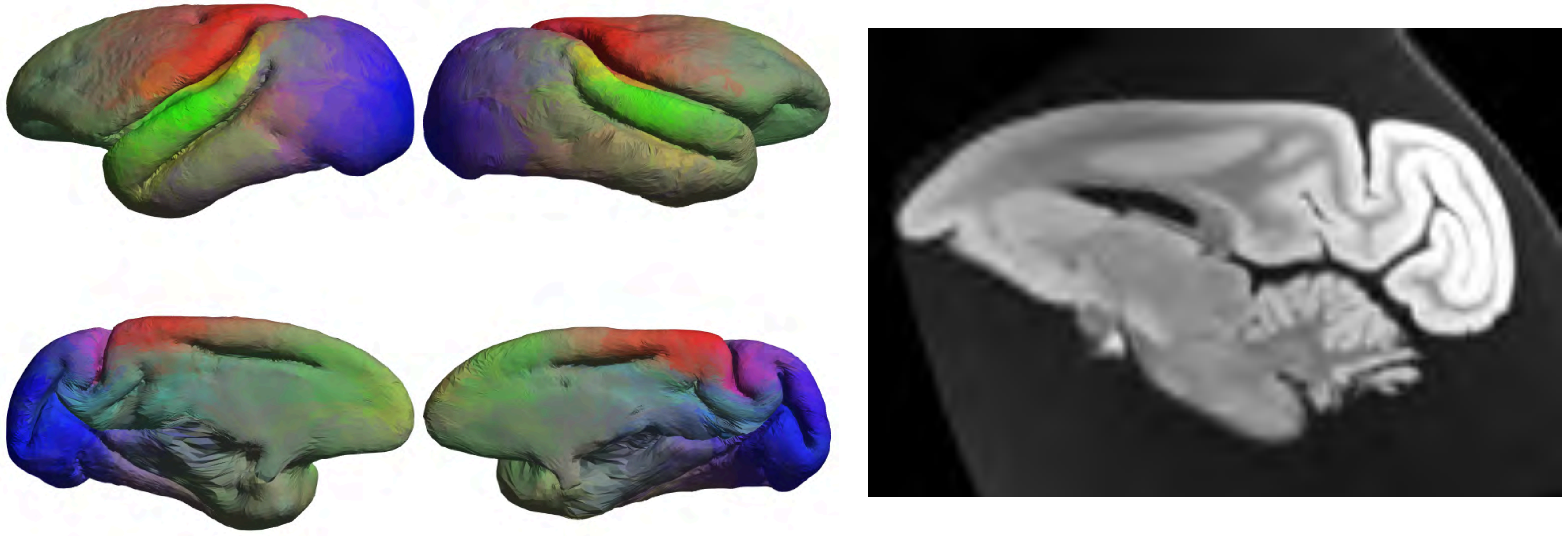

Saimiri sciureus

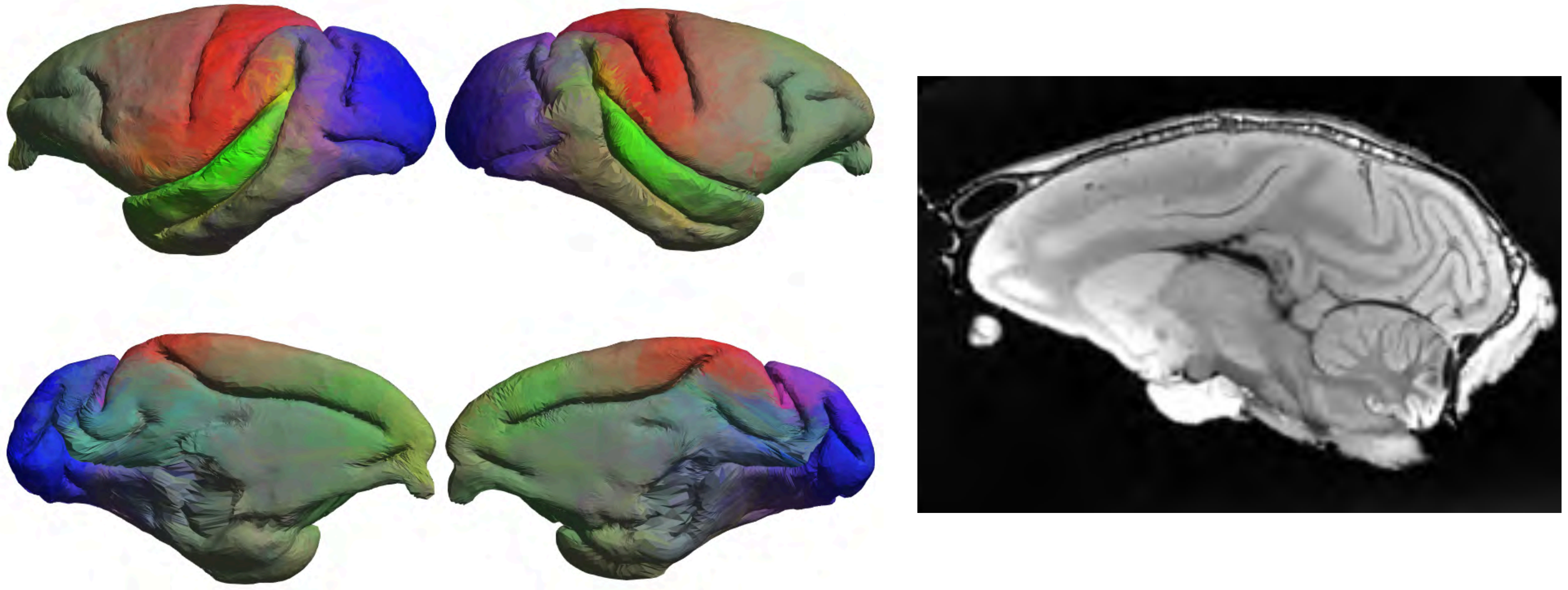

Sapajus apella

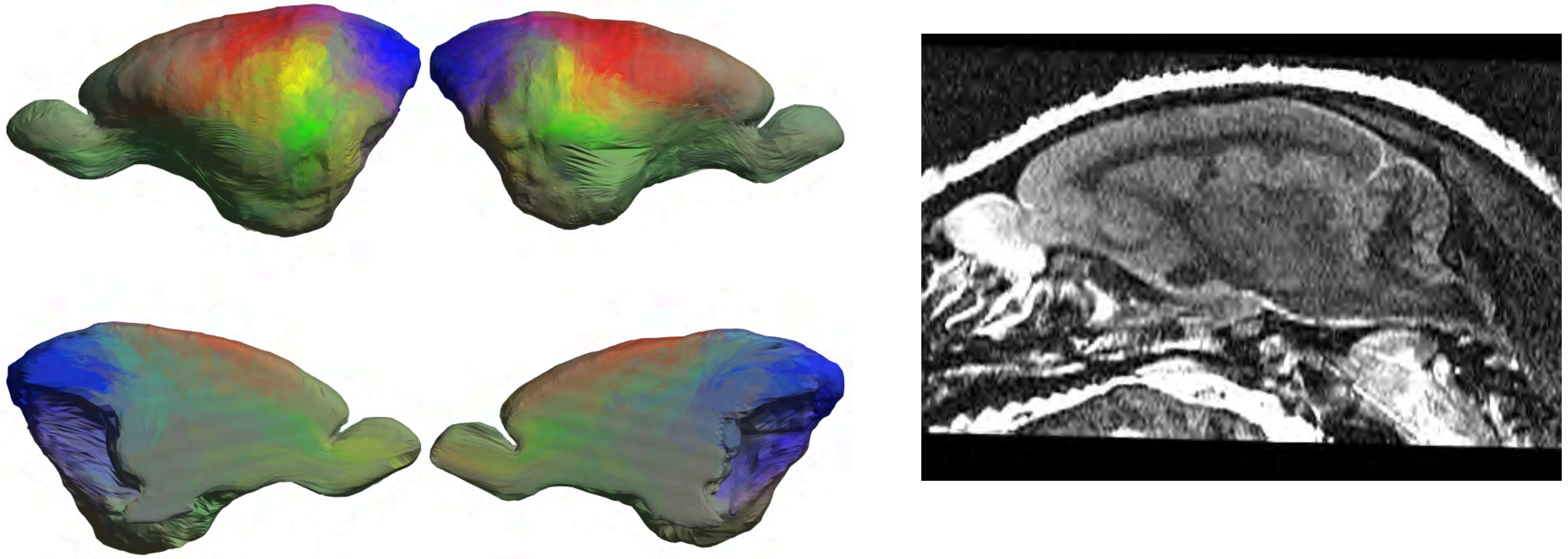

Sciurus carolinensis

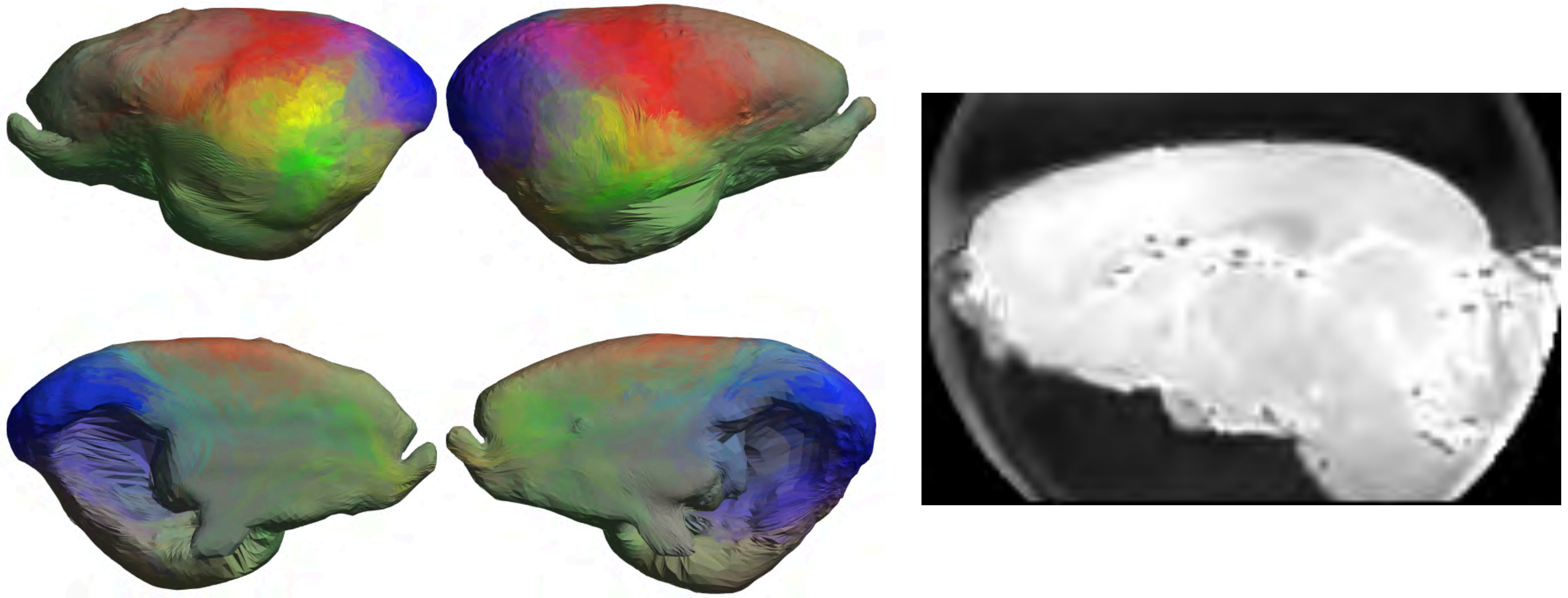

Sciurus vulgaris

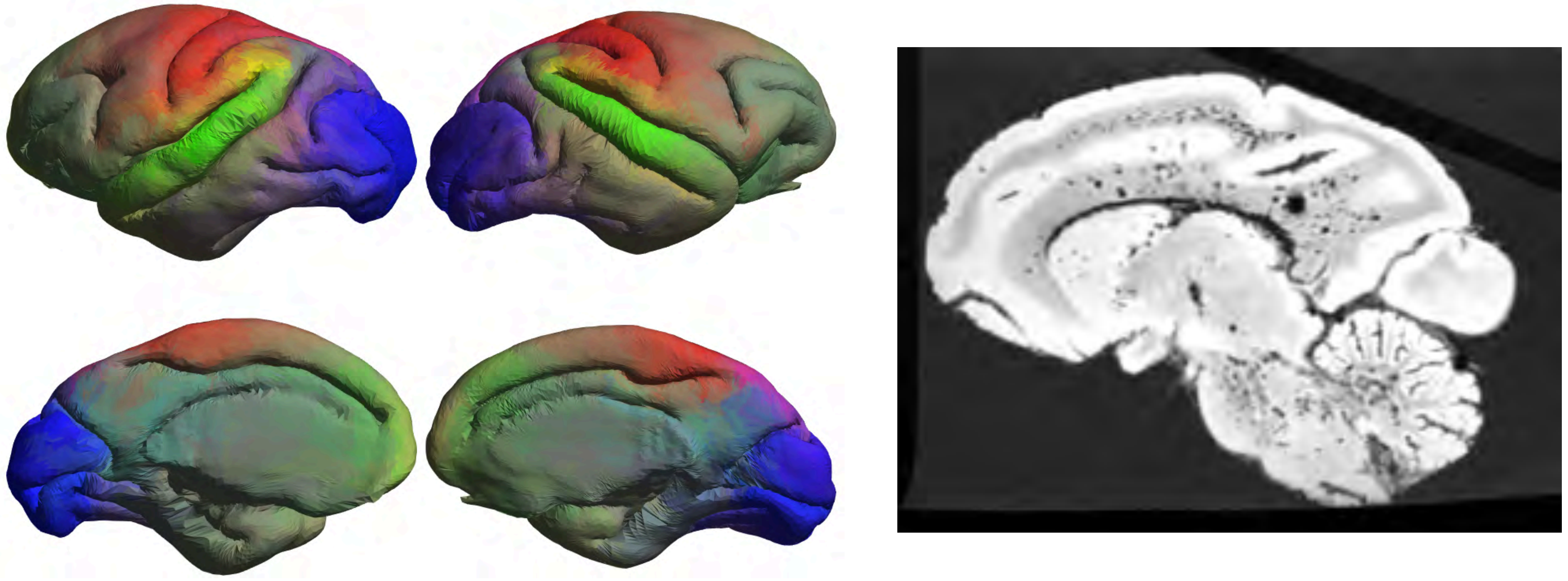

Semnopithecus entellus

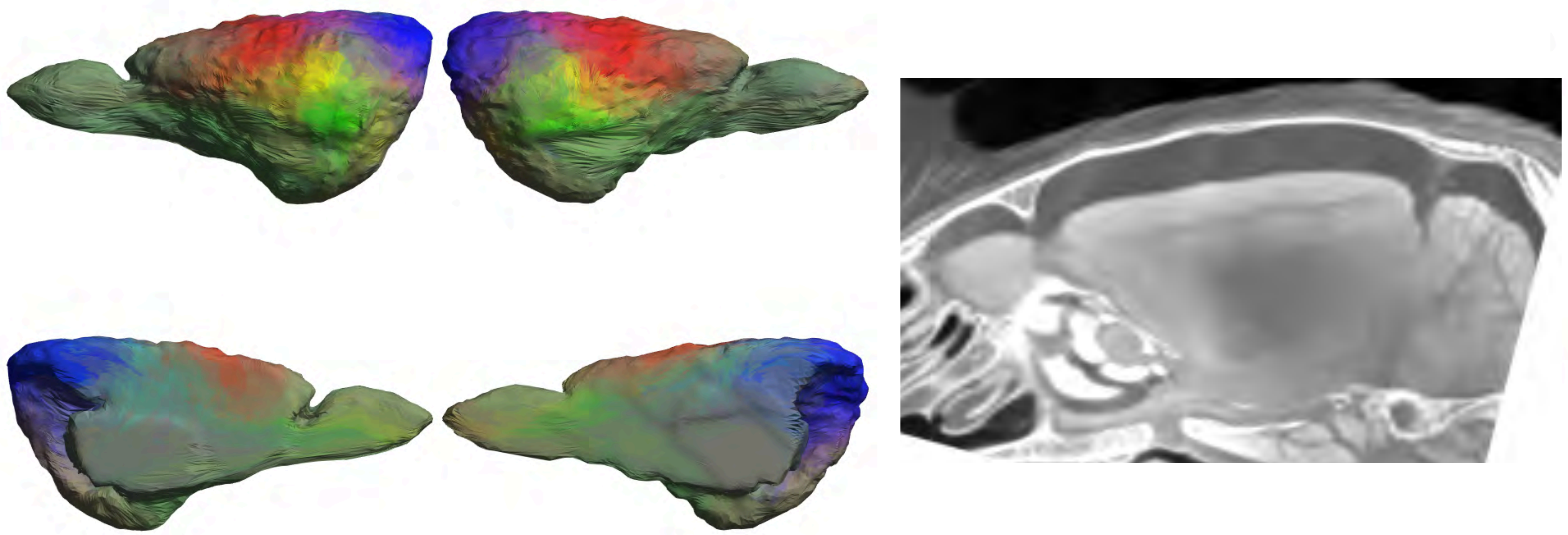

Tamias striatus

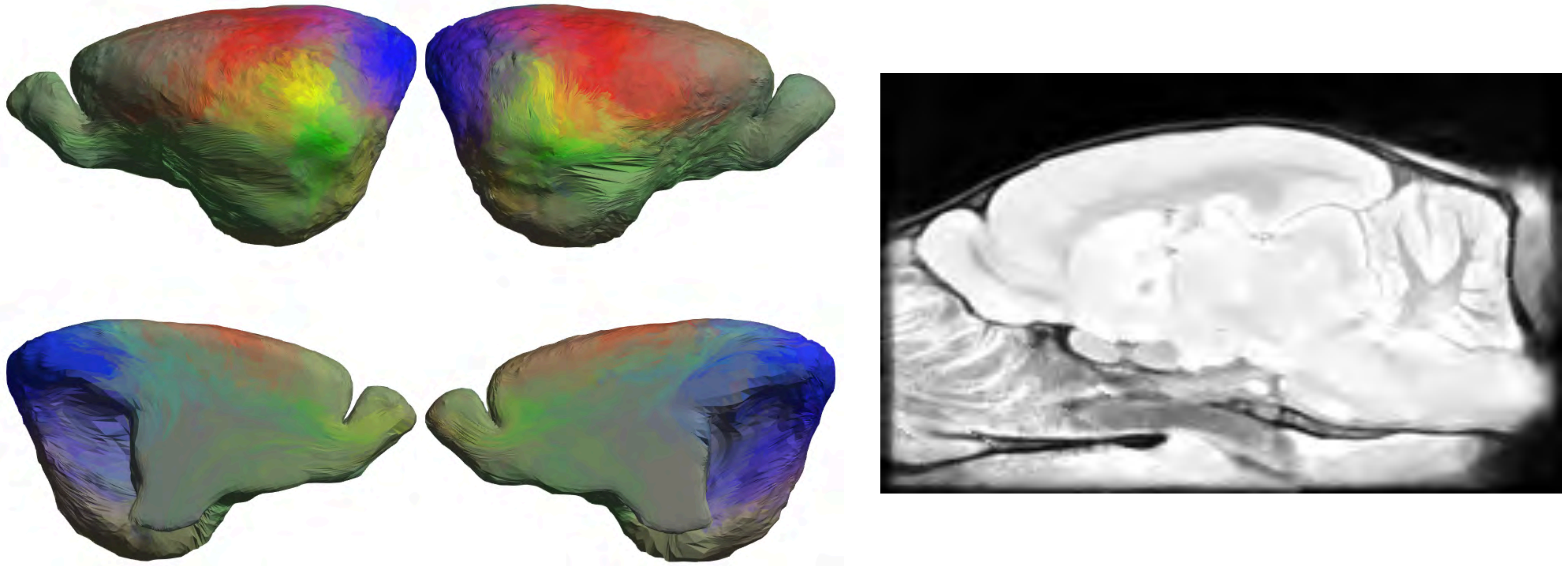

Tamiasciurus hudsonicus

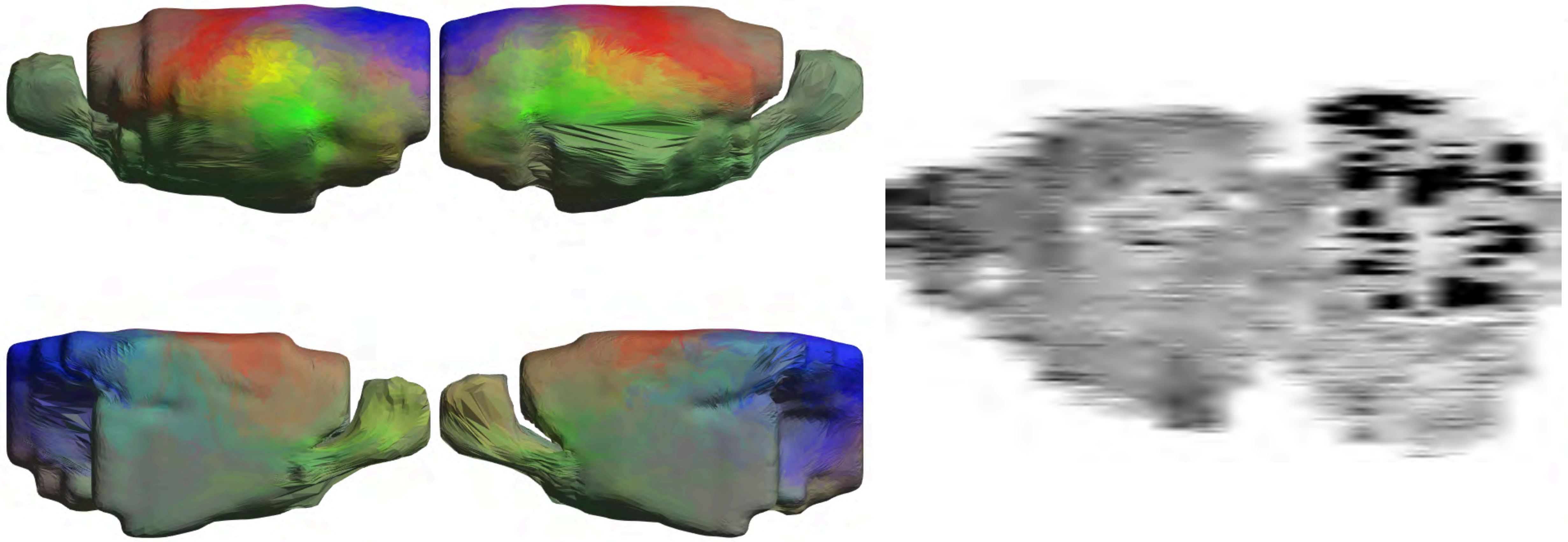

Thomomys talpoides

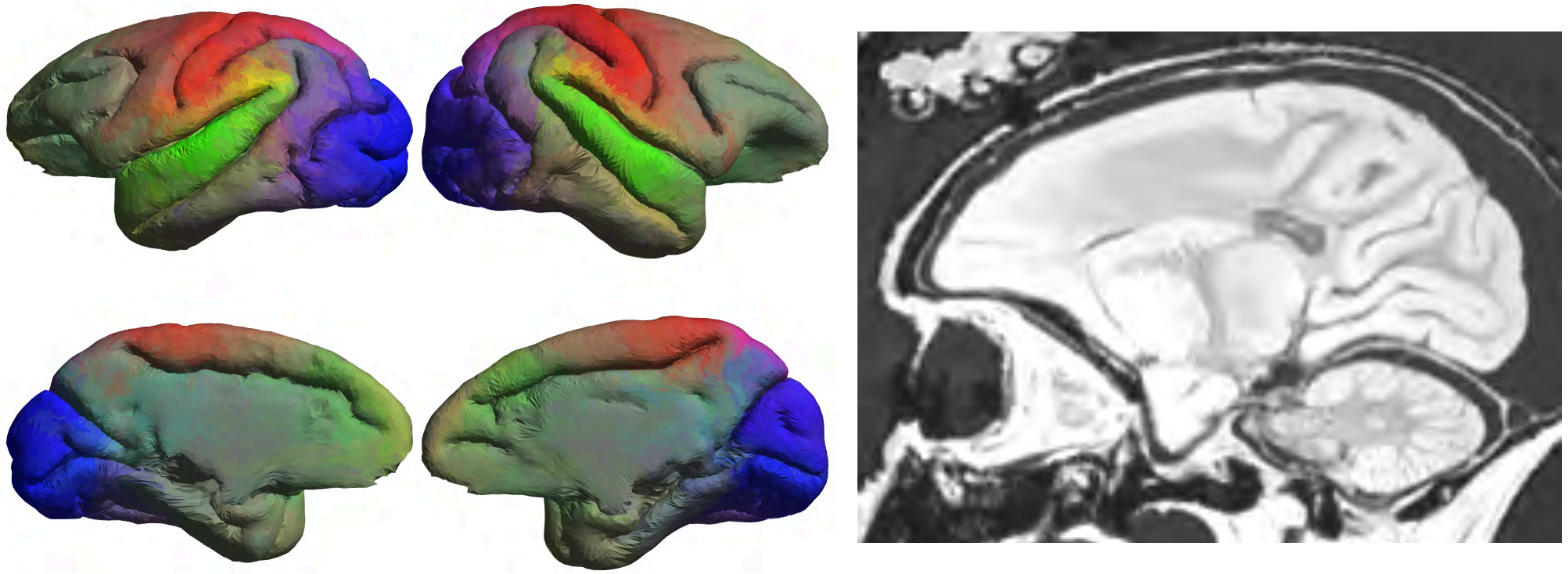

Trachypithecus germaini

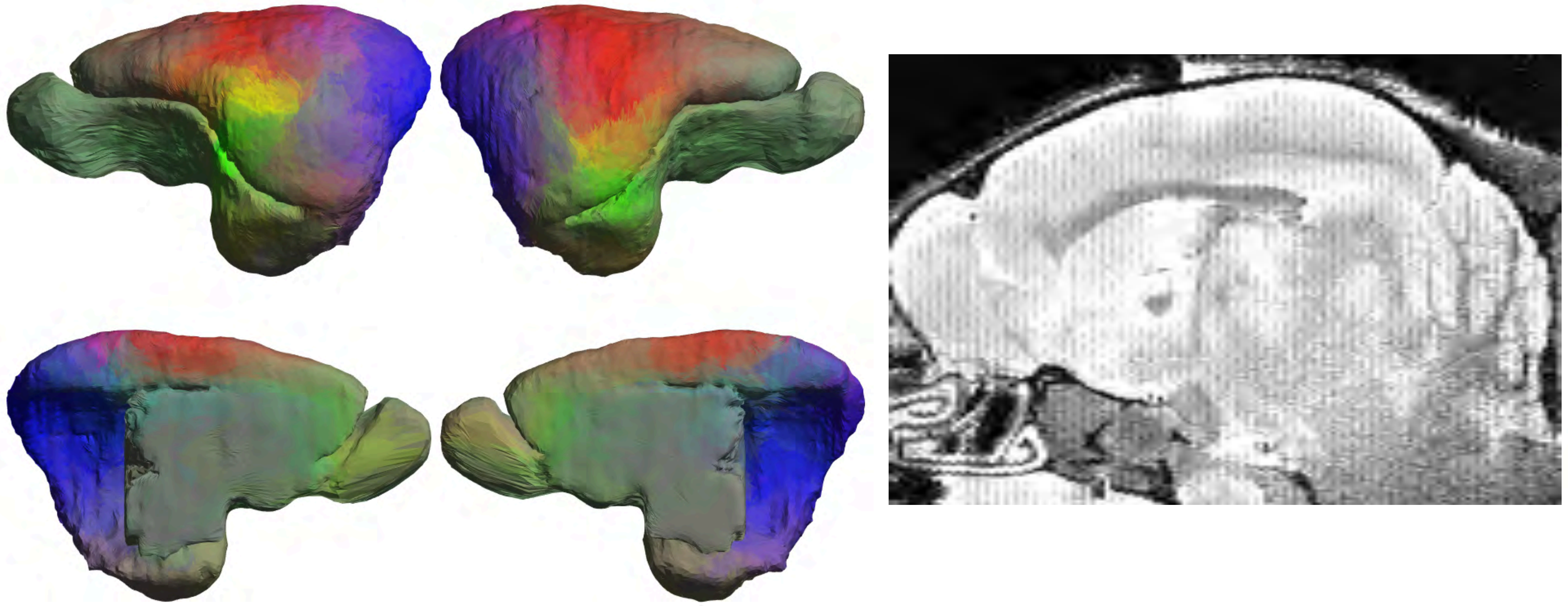

Tupaia belangeri

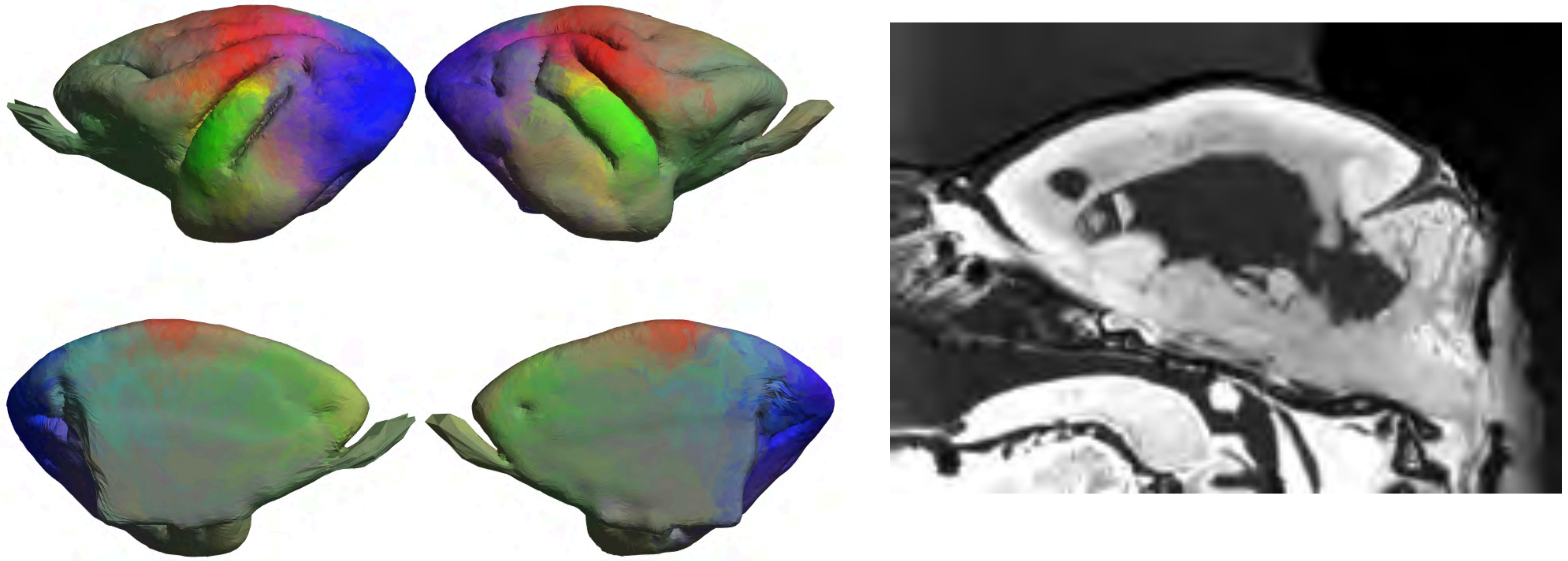

Varecia variegata
