# Supplementary material for: Evolution of cortical geometry and its link to function, behaviour and ecology
Source: Nat Commun. 2023 Apr 20;14:2252. doi: 10.1038/s41467-023-37574-x (PMC10119184; doi:10.1038/s41467-023-37574-x)
Supplement: Supplementary file 15 — Reporting Summary [file 41467_2023_37574_MOESM15_ESM.pdf]

nature portfolio

Corresponding author(s): Prof. Georg Langs

Last updated by author(s): Feb 21, 2023

Reporting Summary

Nature Portfolio wishes to improve the reproducibility of the work that we publish. This form provides structure for consistency and transparency in reporting. For further information on Nature Portfolio policies, see our Editorial Policies and the Editorial Policy Checklist.

Statistics

For all statistical analyses, confirm that the following items are present in the figure legend, table legend, main text, or Methods section.

n/a	Confirmed	
☐	☒	The exact sample size (*n*) for each experimental group/condition, given as a discrete number and unit of measurement
☐	☒	A statement on whether measurements were taken from distinct samples or whether the same sample was measured repeatedly
☐	☒	The statistical test(s) used AND whether they are one- or two-sided<br>*Only common tests should be described solely by name; describe more complex techniques in the Methods section.*
☐	☒	A description of all covariates tested
☐	☒	A description of any assumptions or corrections, such as tests of normality and adjustment for multiple comparisons
☐	☒	A full description of the statistical parameters including central tendency (e.g. means) or other basic estimates (e.g. regression coefficient) AND variation (e.g. standard deviation) or associated estimates of uncertainty (e.g. confidence intervals)
☐	☒	For null hypothesis testing, the test statistic (e.g. *F*, *t*, *r*) with confidence intervals, effect sizes, degrees of freedom and *P* value noted<br>*Give P values as exact values whenever suitable.*
☒	☐	For Bayesian analysis, information on the choice of priors and Markov chain Monte Carlo settings
☒	☐	For hierarchical and complex designs, identification of the appropriate level for tests and full reporting of outcomes
☐	☒	Estimates of effect sizes (e.g. Cohen's *d*, Pearson's *r*), indicating how they were calculated

Our web collection on statistics for biologists contains articles on many of the points above.

Software and code

Policy information about availability of computer code

Data collection	No software was used for data collection.
Data analysis	All analysis was implemented in Matlab R2014a and R2019a, Python 2.7.13 and 3.7.3 as well as R 3.6.0 and 4.0.3. Additional processing was performed using Convert 3D 1.1.0, FSL 6.0.4, FreeSurfer 6.0.0 and 7.1.1, ANTs 2.3.4 and ImageJ 1.49u. Computer codes for individual processing and/or evaluation steps are available from the corresponding author upon reasonable request.

For manuscripts utilizing custom algorithms or software that are central to the research but not yet described in published literature, software must be made available to editors and reviewers. We strongly encourage code deposition in a community repository (e.g. GitHub). See the Nature Portfolio guidelines for submitting code & software for further information.

Data

Policy information about availability of data

All manuscripts must include a data availability statement. This statement should provide the following information, where applicable:

- Accession codes, unique identifiers, or web links for publicly available datasets
- A description of any restrictions on data availability
- For clinical datasets or third party data, please ensure that the statement adheres to our policy

All quantitative data supporting the findings of this study are provided as supplementary information to the article. Sources of all imaging data used in the study as well as reference publications are available in Supplementary Data 1. Aligned surface models used to define the proposed common reference frame, as well as ancestral state estimates obtained from it are publicly available at https://github.com/cirmuw/EvolutionOfCorticalShape. Expansion maps used for meta-analytic

decoding are additionally made publicly available at https://neurovault.org/collections/IHFSXSES/.

Human research participants

Policy information about studies involving human research participants and Sex and Gender in Research.

Reporting on sex and gender	*Use the terms sex (biological attribute) and gender (shaped by social and cultural circumstances) carefully in order to avoid confusing both terms. Indicate if findings apply to only one sex or gender; describe whether sex and gender were considered in study design whether sex and/or gender was determined based on self-reporting or assigned and methods used. Provide in the source data disaggregated sex and gender data where this information has been collected, and consent has been obtained for sharing of individual-level data; provide overall numbers in this Reporting Summary. Please state if this information has not been collected. Report sex- and gender-based analyses where performed, justify reasons for lack of sex- and gender-based analysis.*
Population characteristics	*Describe the covariate-relevant population characteristics of the human research participants (e.g. age, genotypic information, past and current diagnosis and treatment categories). If you filled out the behavioural & social sciences study design questions and have nothing to add here, write "See above."*
Recruitment	*Describe how participants were recruited. Outline any potential self-selection bias or other biases that may be present and how these are likely to impact results.*
Ethics oversight	*Identify the organization(s) that approved the study protocol.*

Note that full information on the approval of the study protocol must also be provided in the manuscript.

Field-specific reporting

Please select the one below that is the best fit for your research. If you are not sure, read the appropriate sections before making your selection.

☒ Life sciences ☐ Behavioural & social sciences ☐ Ecological, evolutionary & environmental sciences

For a reference copy of the document with all sections, see nature.com/documents/nr-reporting-summary-flat.pdf

Life sciences study design

All studies must disclose on these points even when the disclosure is negative.

Sample size	The results of this study are based on volumetric imaging data obtained via various modalities (detailed in the supplementary materials) from 90 individual species of Primates, Rodents, Lagomorphs, Dermoptera and Scandentia. They represent the maximum number of species that the authors could assemble from third parties. The sample size was not determined a priori
Data exclusions	Data of the following species was collected but excluded due to strong distortions in the morphology of the brains (deterioration due to long preservation, inadequate container sizes): Ateles fusciceps, Cercopithecus hamlyni, Erythrocebus patas, Macaca nemestrina, Mandrillus sphinx, Pithecia pithecia (all from the "Primate Brain Bank" dataset doi:10.1159/000488136)
Replication	Unfortunately, no data for the replication of the study is available at the moment, as no collection of imaging data from a comparable number of species in the same phylogeny has been collected to date.
Randomization	Organisms were grouped based on information on preferred habitat, activity time and group size. Unfortunately potential confounds (for example age, sex, image quality) could not be accounted for due to unavailabilty of that information.
Blinding	all evaluations were performed algorithmically, no blinding was necessary to avoid bias

Reporting for specific materials, systems and methods

We require information from authors about some types of materials, experimental systems and methods used in many studies. Here, indicate whether each material, system or method listed is relevant to your study. If you are not sure if a list item applies to your research, read the appropriate section before selecting a response.

Materials & experimental systems

n/a	Involved in the study
☒	☐ Antibodies
☒	☐ Eukaryotic cell lines
☒	☐ Palaeontology and archaeology
☐	☒ Animals and other organisms
☒	☐ Clinical data
☒	☐ Dual use research of concern

Methods

n/a	Involved in the study
☒	☐ ChIP-seq
☒	☐ Flow cytometry
☐	☒ MRI-based neuroimaging

Animals and other research organisms

Policy information about studies involving animals; ARRIVE guidelines recommended for reporting animal research, and Sex and Gender in Research

Laboratory animals	imaging data obtained from laboratory animals were obtained from published sources and/or third parties only
Wild animals	no animals were caught specifically for this study
Reporting on sex	sex was not considered in this study
Field-collected samples	no field-collected samples were obtained specifically for this study
Ethics oversight	all data used in this study were obtained from published sources with appropriate ethics oversight

Note that full information on the approval of the study protocol must also be provided in the manuscript.

Magnetic resonance imaging

Experimental design

Design type	Only structural MRI was used in this study
Design specifications	Of the 90 species used in the study, 75 were imaged using MRI. As only third party data was used in this study, design specifications varied and are described in detail in the corresponding publications listed in Supplementary Table 1
Behavioral performance measures	no behavioral performance was assessed in this study

Acquisition

Imaging type(s)	structural
Field strength	various field strengths specified in Supplementary Table 1
Sequence & imaging parameters	various imaging parameters specified in Supplementary Table 1
Area of acquisition	Whole brain scans only

Diffusion MRI ☐ Used ☒ Not used

Preprocessing

Preprocessing software	no preprocessing was performed
Normalization	Whole brain volumes were manually rotated to approximately position the AC/PC line in the axial plane.
Normalization template	the data were not normalized
Noise and artifact removal	no denoising was performed
Volume censoring	no volume censoring was performed

Statistical modeling & inference

Model type and settings	Correlation between surface expansion maps and relevance maps corresponding to individual neuroscientific terms were computed as in Yarkoni, T., Poldrack, R. A., Nichols, T. E., Van Essen, D. C. & Wager, T. D. Large-scale automated synthesis of human functional neuroimaging data. Nat. Methods 8, 665–670 (2011). (Supplementary Tables 3a, 5)<br>Repeated Measurements ANOVA was used to analyse a sequence of expansion-term correlations.

Either Pearson or Spearman correlation (depending on normality, determined via Kolmogorov-Smirnov tests) was used depending on the normality of the data when assessing relationship between any two variables that are spatially independent.
Surrogate-based statistics were used to assess the significance of results in specific cortical parcellations (eg. Burt, J. B., Helmer, M., Shinn, M., Anticevic, A. & Murray, J. D. Generative modeling of brain maps with spatial autocorrelation. Neuroimage 220, 117038 (2020) and Weinstein, S. M. et al. A simple permutation-based test of intermodal correspondence. Hum. Brain Mapp. 42, 5175–5187 (2021).
Linear Modeling was used to assess the influence of species and time on the relative size of individual cortical parcels (as per surrogate model distribution, see above) in the evolution of mice and men (Supplementary Table 4b)
Kruskall-Wallis under surrogate-based statistics followed by post-hoc pairwise Wilcoxon-Rank-Sum/Mann-Whitney U-Test (also surrogates-based) was used to determine the significance of effects of cortical expansions in specific cortical regions (eg. Yeo, B. T. T. et al. The organization of the human cerebral cortex estimated by intrinsic functional connectivity. J. Neurophysiol. 106, 1125–1165 (2011).) (Supplementary Table 6)
Friedman tests followed by Nemenyi post-hoc tests were used to assess the effect of specific habitat on relative expansion of individual cortical areas (Supplementary Table 7b)
Repeated Measurements Correlation (RMCORR, Bakdash, J. Z. & Marusich, L. R. Repeated Measures Correlation. Front. Psychol. 8, 456 (2017).) was used to assess the stability of species habitat on the relative expansion of individual cortical areas (Supplementary Table 7c)
Depending on value distribution (assessed via Kolmogorov-Smirnov tests), Wilcoxon-Rank-Sum/Mann-Whitney U-Test or two-sample t-tests were used to assess the significance of differences in range parameter of spatial statistical models of modal specificity (see Supplementary Methods, Supplementary Table 8a)
Linear modelling was used to assess the relationship between evolutionary change in this range parameter and deep time in the human lineag (Supplementary Table 8b)
Pearson correlation was used to assess the relationship between progression of meta-analytical term decodings of evolutionary cortical surface expansion and estimates of ancestral likelihoods of socio-ecological factors (Supplementary Table 9a). Partial correlation analysis of these values (controlling for diurnality) was performed with confidence intervals determined by 10000 bootstrap iterations (Supplementary Table 9b)

Effect(s) tested: no task or stimulus conditions were tested in this study

Specify type of analysis: ☒ Whole brain ☐ ROI-based ☐ Both

Statistic type for inference (See Eklund et al. 2016): no statistical inference was performed in this study

Correction: FDR correction was performed to correct for multiple comparisons. Phylogeny was accounted for in all computations.

Models & analysis

n/a	Involved in the study
☒	☐ Functional and/or effective connectivity
☒	☐ Graph analysis
☒	☐ Multivariate modeling or predictive analysis
